# 3D-Printable, Honeycomb-Inspired Tissue-Like Bioelectrodes for Patient-Specific Neural Interface

**DOI:** 10.1002/adma.202516291

**Published:** 2026-03-14

**Authors:** Marzia Momin, Luyi Feng, Xiaoai Chen, Salahuddin Ahmed, Basma AlMahmood, Li-Pang Huang, Jiashu Ren, Xinyi Wang, Hyunjin Lee, Samuel R. Cramer, Nanyin Zhang, Sulin Zhang, Tao Zhou

**Affiliations:** 1 Department of Engineering Science and Mechanics, The Pennsylvania State University, University Park, Pennsylvania, USA; 2 Department of Biomedical Engineering, The Pennsylvania State University, University Park, Pennsylvania, USA; 3 Department of Physics, The Pennsylvania State University, University Park, Pennsylvania, USA; 4 Department of Biology, The Pennsylvania State University, University Park, Pennsylvania, USA; 5 Department of Mechanical Engineering, The Pennsylvania State University, University Park, Pennsylvania, USA; 6 Center For Neural Engineering, The Pennsylvania State University, University Park, Pennsylvania, USA; 7 Huck Institutes of the Life Sciences, The Pennsylvania State University, University Park, Pennsylvania, USA; 8 The Neuroscience Graduate Program, The Pennsylvania State University, University Park, Pennsylvania, USA; 9 Department of Materials Science and Engineering, The Pennsylvania State University, University Park, Pennsylvania, USA; 10 Materials Research Institute, The Pennsylvania State University, University Park, Pennsylvania, USA

**Keywords:** 3D printing, bioinspired, brain-computer interface, ECoG, neural interface, patient-specific, soft electrodes

## Abstract

The unique gyral patterns of the human brain demand patient-specific neural interfaces to achieve precise neuromodulation, mitigate adverse tissue responses, and optimize therapeutic efficacy and safety. One-size-fits-all, conventional rigid electrocorticography (ECoG) electrodes, standardized for mass production through lithographic techniques, exhibit limited conformability to the brain’s heterogeneous cortical topography. This mechanical mismatch results in poor electrode-tissue contact, signal loss, and foreign body responses. To address these limitations, we present an integrated novel platform, synergizing MRI-based anatomical mapping, finite element analysis (FEA)—optimized mechanical design, and direct ink writing (DIW) 3D printing to fabricate electrodes customized to individual gyral patterns. The resulting honeycomb-inspired printable gel electrode (HiPGE) employs a bioinspired honeycomb architecture with ultra-soft hydrogels, engineered to match the bending stiffness of brain tissue (0.1–10 kPa) while maintaining cost-efficiency and long-term durability. This mechanical congruence ensures exceptional cortical conformability and adaptive interfacing, circumventing the geometric and material limitations of traditional rigid electrodes. By combining patient-specific design with scalable fabrication, our platform establishes a transformative framework for neural interface engineering, enhancing precision, biocompatibility, and functional performance in neuromodulation therapies and neuroprosthetic applications.

## 1 | Introduction

The human cerebral cortex features complex folds formed through gyrification, packing billions of neurons in a multilayered organizational structure [1, 2]. Individual variability in gyral patterns, possibly shaped by factors such as sex, age, weight, height, or handedness [3, 4], creates a unique “fingerprint” for each brain. This folded structure, consisting of ridges (gyri) and grooves (sulci), maximizes cortical surface area within the skull, enhancing neural connectivity and information processing efficiency [5]. These patterns also offer critical insights into cognitive functions and neurological disease mechanisms [6, 7], which underscores the critical need to develop personalized neural interfaces for the analysis of topography-function relationships. Indeed, electrodes tailored to the topography are essential for neural activity recording and modulation in applications such as speech recognition [8] and prosthetic control [9, 10], where precise neural signal recording is essential to rehabilitate functions lost to injuries or diseases [11–13].

Conventional neural interfaces, including multi-channel electrocorticography (ECoG) systems [14, 15], often rely on rigid, lithography-fabricated electrodes standardized for mass production [16–18]. Their “one-size-fits-all” design fails to adapt to individualized brain shapes, leading to poor electrode-tissue contact and signal degradation [19, 20] due to scar formation, thereby necessitating extensive signal processing that may introduce bias and undermine data reliability [21, 22]. The stiffness mismatch with soft brain tissues may amplify micromotions from physiological processes such as respiration and blood flow, exacerbating tissue displacement [23, 24], signal instability, and foreign body responses [25–27]. While soft materials-based electrodes have been explored [28, 29], the inability to customize them for patient-specific gyral patterns persists, resulting in tissue deformation, compromised biocompatibility, and degraded signal quality.

To address these limitations, we present a bioinspired neural interface platform integrating three core innovations: patient-specific MRI scans for cortical mapping, finite element analysis (FEA)-guided designs to optimize electrode geometry and softness for pre-implantation safety and efficacy, and direct ink writing (DIW) 3D printing [30, 31] for scalable, patient-specific device fabrication. Central to this platform is the honeycomb-inspired printable gel electrode (HiPGE), a hydrogel-based electrode, engineered with a biomimetic honeycomb structure and tissue-matching softness to mirror the mechanical properties of brain tissue. Biomimetic honeycomb structure offers exceptional cost and material efficiency, lightweight, and durability [32, 33]. Through systematic evaluation of electrode placement and trajectory optimization, HiPGE’s patient-specific design seamlessly conforms to cortical contours, eliminating mechanical mismatch. This ensures high-fidelity electrophysiological recordings across diverse cortical geometries while preventing tissue deformation or chronic inflammatory responses post-implantation. By synergizing cost-effective 3D fabrication with biomimetic engineering principles, our state-of-the-art platform achieves unprecedented functional stability in dynamic neural environments. This synergy advances precision monitoring of localized field potential and establishes a transformative framework for next-generation neuroprosthetics and personalized neuromodulation therapies.

## 2 | 3D Reconstruction of Patients’ Brains to Design HiPGE

**FIGURE 1 |:**
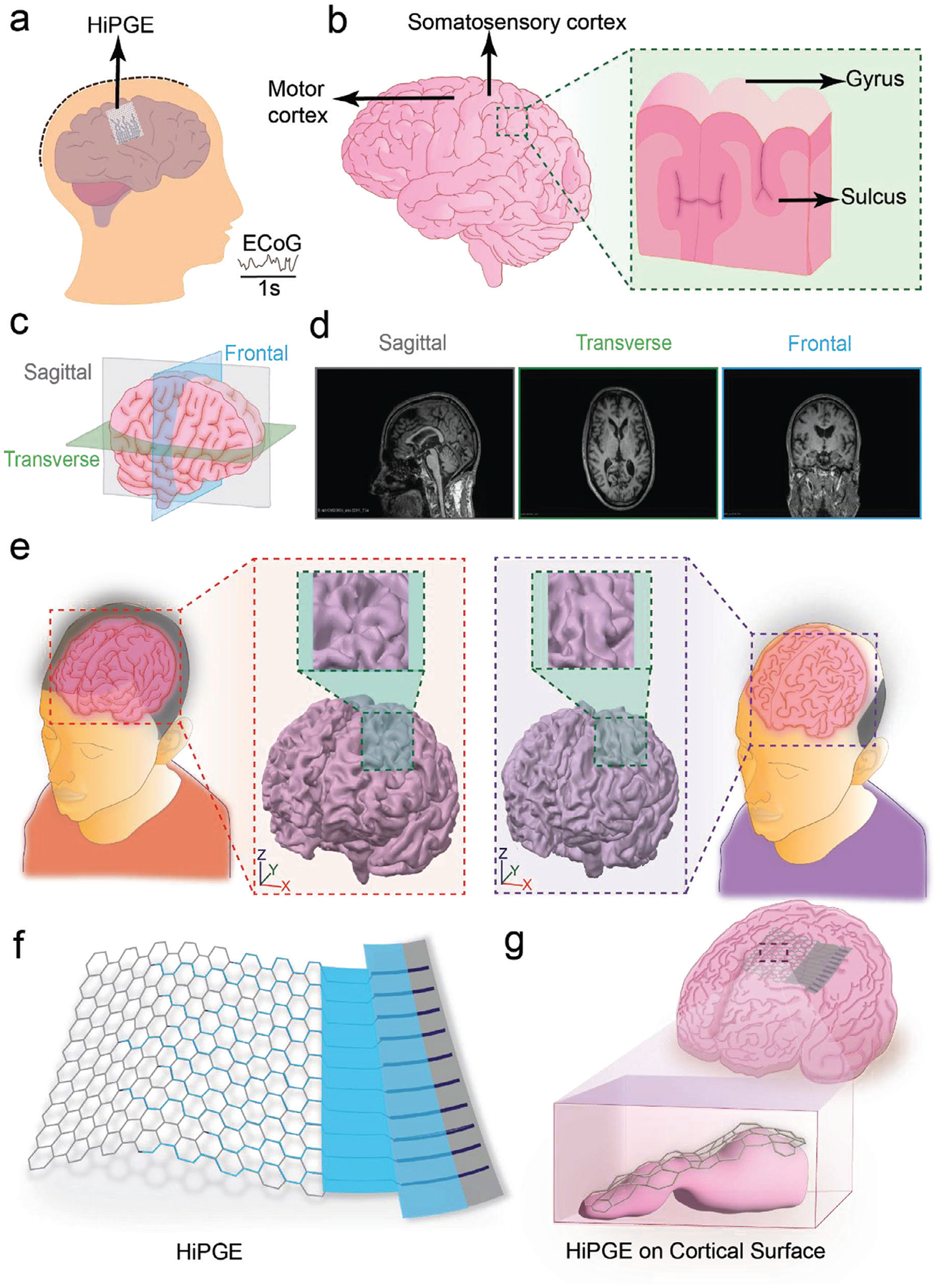
Overview and designs of personalized HiPGE for ECoG recording. (a) Schematic of ECoG recording with a personalized HiPGE from somatosensory and motor cortex as designated ROIs. (b) Schematic of ROI illustrates the brain and zoom-in view of an anatomical structure of the cortical surface, demonstrating gyrus and sulcus. (c,d) Schematic of three anatomical planes (c) and demonstration of MRI structural images of sagittal plane, transverse plane, and frontal plane (d). (e) Illustration of the differences of individuals’ cortical surface and its zoom-in cross-sectional view of gyral patterns (patient 330 – Left-handed and patient 349 – Ambidextrous). (f) Representative of the designs of HiPGE. (g) The side view of the implanted HiPGE conformed to the cortical surface.

In neuroscience research and clinical applications, ECoG offers a significant advantage by enabling high temporal resolution recording of cortical electrophysiological signals (Figure 1a,b), providing invaluable insights into cortical dynamics [34]. To design patient-specific neural electrodes for ECoG recordings and ensure maximum electrode contact with the brain based on individual brain size, structure, and gyri patterns, MRI imaging was utilized. By providing a clear delineation of gyri variations from structural images [35] (Figure 1c,d), MRI imaging enables guided design of personalized neural electrodes.

To account for cross-individual variability [36, 37] in cortical folding, we reconstructed 3D brain models from MRI data of 21 patients from the Open Access Series of Imaging Studies (OASIS) database using 3D Slicer (Figure S1, details in Methods) to analyze individual gyral patterns of the brains. These models revealed significant inter-subject differences in brain sizes and gyral complexity (Figure 1e; Tables S1 and S2), highlighting the inadequacy of conventional one-size-fits-all neural implants. By integrating MRI-based surface curvature analysis and region-of-interest (ROI) mapping, we generated patient-specific neural electrode designs tailored to individual cortical geometries.

Inspired by natural honeycomb structures, HiPGE (Figure 1f) employs hexagonal lattice units to maximize cortical surface coverage while maintaining isotropic elasticity through its six-fold rotational symmetry [38]. Building on this principle, HiPGE incorporates multiple hexagonal lattices to form a porous architecture that enhances mechanical strength, flexibility, and conformability to uneven surfaces [32, 33] (Figure 1g). Recent studies have demonstrated that fluids in the cranial space are essential for brain functions and neurological disorders [39–41]. Our design minimizes disruption to native brain tissue and allows unrestricted fluid exchange post-implantation, a critical advantage over conventional ECoG electrodes, which often obstruct fluid dynamics. The honeycomb design also enables cost-effective, massive fabrication of personalized electrodes, reducing healthcare costs and broadening accessibility.

## 3 | FEA-Guided Design and Mechanics Evaluation of HiPGE

**FIGURE 2 |:**
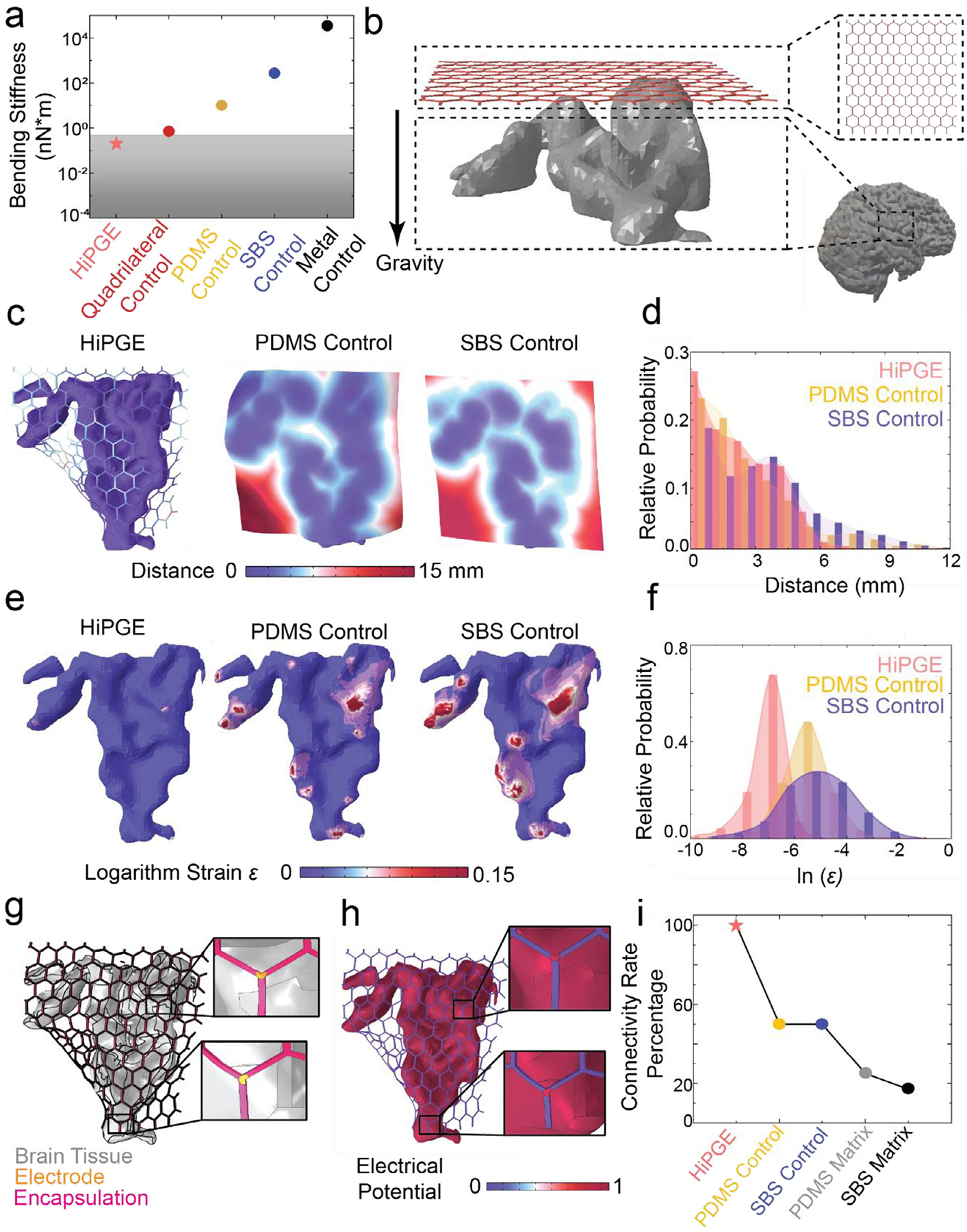
Mechanical and electrical properties of HiPGE from simulations. (a) Comparison of bending stiffness of HiPGE (pink star), brain tissue (grey rectangular), and other control devices, quadrilateral control (red circle) [31], PDMS control (yellow circle), SBS control (blue circle), and Metal control (black circle). (b) Schematic illustration for conformability simulation setup. HiPGE (pink honeycomb structure) suspends on top of a part of the MRI-reconstructed 3D brain model’s tissue (grey domain) and drops down to conform to the brain surface out of gravity. (c) The final configurations of devices attached to the brain tissue after conformability simulation. A color map shows the distance from the devices to the brain surface for HiPGE, PDMS control, and SBS control. (d) The probability histogram shows the distance distribution for HiPGE (pink column), PDMS control (yellow column), and SBS control (blue column). (e) The deformed brain tissue after conformability simulation. A color map shows the logarithm strain distribution on the brain tissue for HiPGE, PDMS control, and SBS control. (f) The probability histogram shows the logarithm strain distribution for HiPGE (pink column), PDMS control (yellow column), and SBS control (blue column). (g) Schematic illustration of HiPGE on the brain surface. The brain surface with constant electrical potential, electrode, and PDMS encapsulation are represented by grey, yellow, and pink domains, respectively. (h) Connectivity simulation results. The electrodes on HiPGE show good signal connectivity. (i) Connectivity rate of different devices, including HiPGE (pink star), PDMS control (yellow circle), SBS control (blue circle), PDMS matrix (grey circle), and SBS matrix (black circle).

The human cerebral cortex exhibits a soft, convoluted structure with a low Young’s modulus of 0.1–10 kPa [42, 43]. To minimize mechanical mismatch and tissue damage, HiPGE employs low-stiffness materials such as polydimethylsiloxane (PDMS) and poly (3,4-ethylene dioxythiophene): polystyrene sulfonate (PEDOT: PSS)-based hydrogels [44]. However, experimental quantification of critical metrics, including bending stiffness, cortical contact area, and implantation-induced strain, remains labor-intensive and prone to experimental error. We thus integrated finite element analysis (FEA) to optimize device geometry, enhancing device design efficiency and precision. FEA revealed the bending stiffness of HiPGE closely matched brain tissue (Figure 2a; Figure S2), outperforming non-porous controls, including PDMS, polystyrene-*block*-polybutadiene-*block*-polystyrene(SBS) [45], and metal. The honeycomb architecture reduced the bending stiffness threefold compared to quadrilateral porous designs [31], given the same material. These results are consistent with previous studies on honeycomb structures, further validating the advantages of this design [32, 46].

To further investigate the conformability of HiPGE on the cortical surface, the intimate contact between the probe and brain tissue was modeled during the implantation process. Given the small strain rate and negligible viscous-induced stress, viscoelastic effects were not included in the current modeling. However, such effects should be considered for chronic and cyclic loading conditions, if present. Simulated gravitational settling of a device-brain assembly, consisting of the device suspended above bottom-fixed, non-viscous, hyperelastic brain tissue (Figure 2b; details in Methods), demonstrated HiPGE’s superior adaptation to cortical topography. Distance contour maps (Figure 2c,d) showed that HiPGE maintained closer contact (average distance: 2.66 mm) compared to PDMS (4.16 mm) and SBS (5.22 mm). Strain distribution analysis further confirmed that HiPGE induced minimal tissue deformation, contrasting sharply with the rigid control devices (Figure 2e,f). HiPGE’s patient-specific electrode placement, guided by gyral patterns, was validated by in silico modeling [47] (Figure 2g,h). Simulating constant electrical potential (brain set to constant potential), only exposed electrodes (not passivated by the encapsulation layers) recorded signals, with the signal connectivity dependent on physical proximity to the cortical surface. The connectivity rate (connected electrodes/total electrodes) quantifies signal recording efficacy. As shown in Figure 2i, HiPGE achieved a near-perfect connectivity rate (Figure S3), surpassing the PDMS and SBS controls (Figure S4) and non-personalized probes with matrix-aligned electrodes [45] (Figure S5). The modeling-guided electrode placement of HiPGE, combined with the soft mechanics, eliminated signal gaps caused by poor contact in rigid controls.

## 4 | Fabrication of HiPGE

**FIGURE 3 |:**
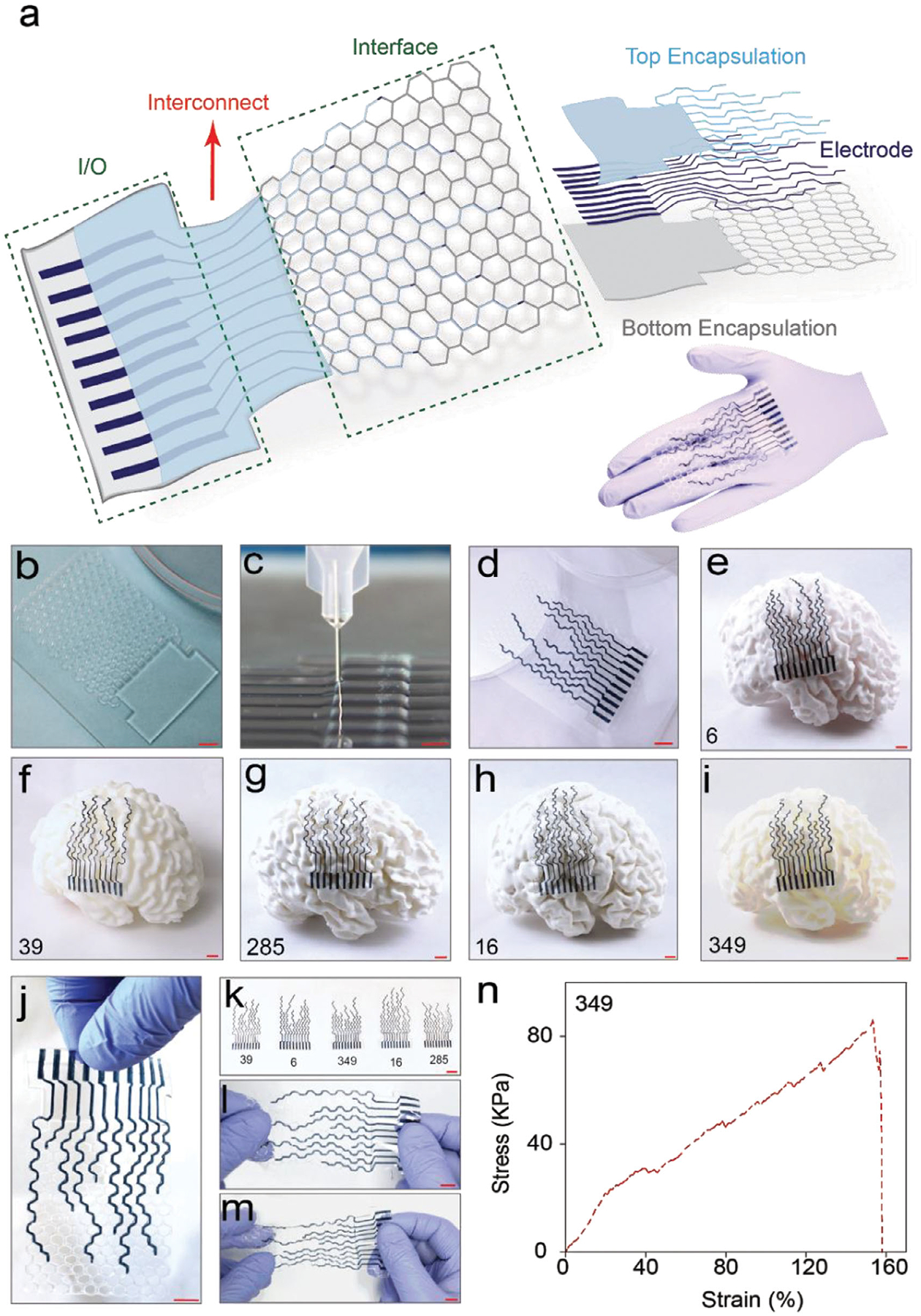
Personalized 3D printable HiPGE for individual patients’ brains. (a) Schematic illustrations of a HiPGE (with three layers: bottom, electrode, and top; three parts: interface, interconnect, and I/O part) and a photograph of 3D printed HiPGE. (b) A photograph of a printed bottom layer on a glass substrate for an individual. Scale bar, 1 cm. (c) An image of printing the top layer of the device. Scale bar, 1 cm. (d) Illustration of a personalized HiPGE printed on a glass substrate. Scale bar, 1 cm. (e–i) Five patient-specific HiPGEs are conformed to their corresponding individual patient’s right hemisphere of the brain model (e—patient 6, f—patient 39, g—patient 285, h—patient 16, i—patient 349). Scale bars, 1 cm. (j) Demonstration of easy handling of a HiPGE. Scale bar, 1 cm. (k) A photograph illustrating the designs and the sizes of five HiPGEs tailored for five different patients. Scale bar, 2 cm. (l–m) Photographs of a HiPGE without (l) and with (m) stretching position. Scale bars, 1 cm. (n) Engineering stress vs engineering strain curve for a HiPGE designed for patient 349.

After successfully verifying the mechanics superiority of HiPGE, five patients were randomly selected from 21 reconstructed 3D brain models to design personalized HiPGEs tailored to their individual brain architecture. The pathways and positions of electrodes were first guided by prominent anatomical landmarks, including the longitudinal fissure, central sulcus, and lateral sulcus (Figure S6), with the sensorimotor (SM) regions designated as ROIs. Based on these anatomical references, five designs were developed for the right hemispheres of the brains (Figures S7 and S8), with electrode configurations tailored to the gyri contours obtained from anatomical measurements in 3D Slicer-based brain models. Following the design process, PDMS was used for encapsulation layers (bottom and top), and a soft and stretchable bi-continuous conducting polymer hydrogel consisting of PEDOT: PSS and polyurethane [48] was used for the electrode layer (Figure 3a). The HiPGE consists of three main parts: the interface (the part that directly interfaces with the cortical surface), the interconnect (the part that bridges the interface and I/O part), and the I/O (input/output for the connection to the recording system). These sandwich-structured devices were fabricated on glass substrates (Figure 3b; Figure S9, details in Methods) using DIW 3D printing technology (Figure 3c,d).

Upon fabricating the HiPGEs, we demonstrated the practical application of our approach by processing five reconstructed 3D brain models using Creality Slicer (details in Methods, Figure S10a), followed by importing them into FDM (Fused Deposition Method) 3D printing technology (Figure S10b) for fabrication. The resulting printed brain models were used to showcase how personalized neural implants conform to distinctive brain anatomies, highlighting the devices’ conformability to the cortical surface and the precise alignment of electrode positions with individual gyri structures. The personalized HiPGEs demonstrated precise conformity to the unique shapes and spatial arrangements of the gyri in each individual, as depicted in Figure 3e–i. In addition, the devices exhibit robustness and ease of handling, as shown in Figure 3j. The variations in device dimensions, reflecting the anatomical differences among patients, are clearly demonstrated in Figure 3k. Furthermore, the HiPGEs show excellent stretchability (Figure 3l,m), demonstrating its robustness in dynamic cortical environments. To quantitatively evaluate this property, tensile testing was conducted, which revealed that the HiPGEs are highly stretchable with an ultimate strain of up to 160% (Figure 3n; Figures S11 and S12). All five different patients’ brain models with their personalized devices are exquisitely displayed in Figure S13, highlighting the precision and adaptability of the HiPGE designs according to individual brain structures and gyri patterns. To further quantify the superiority of personalization, a direct comparative analysis between patient-specific and non-patient-specific designs was performed. Both electrode types were dyed blue for enhanced contrast and carefully placed on 3D brain model surfaces to assess their cortical coverage. Photographs captured after placement clearly reveal the difference in conformity, while quantitative analysis demonstrates a substantially higher percentage of electrode-cortex contact for patient-specific designs (Figure S14).

## 5 | Electrical Characterization of HiPGE

**FIGURE 4 |:**
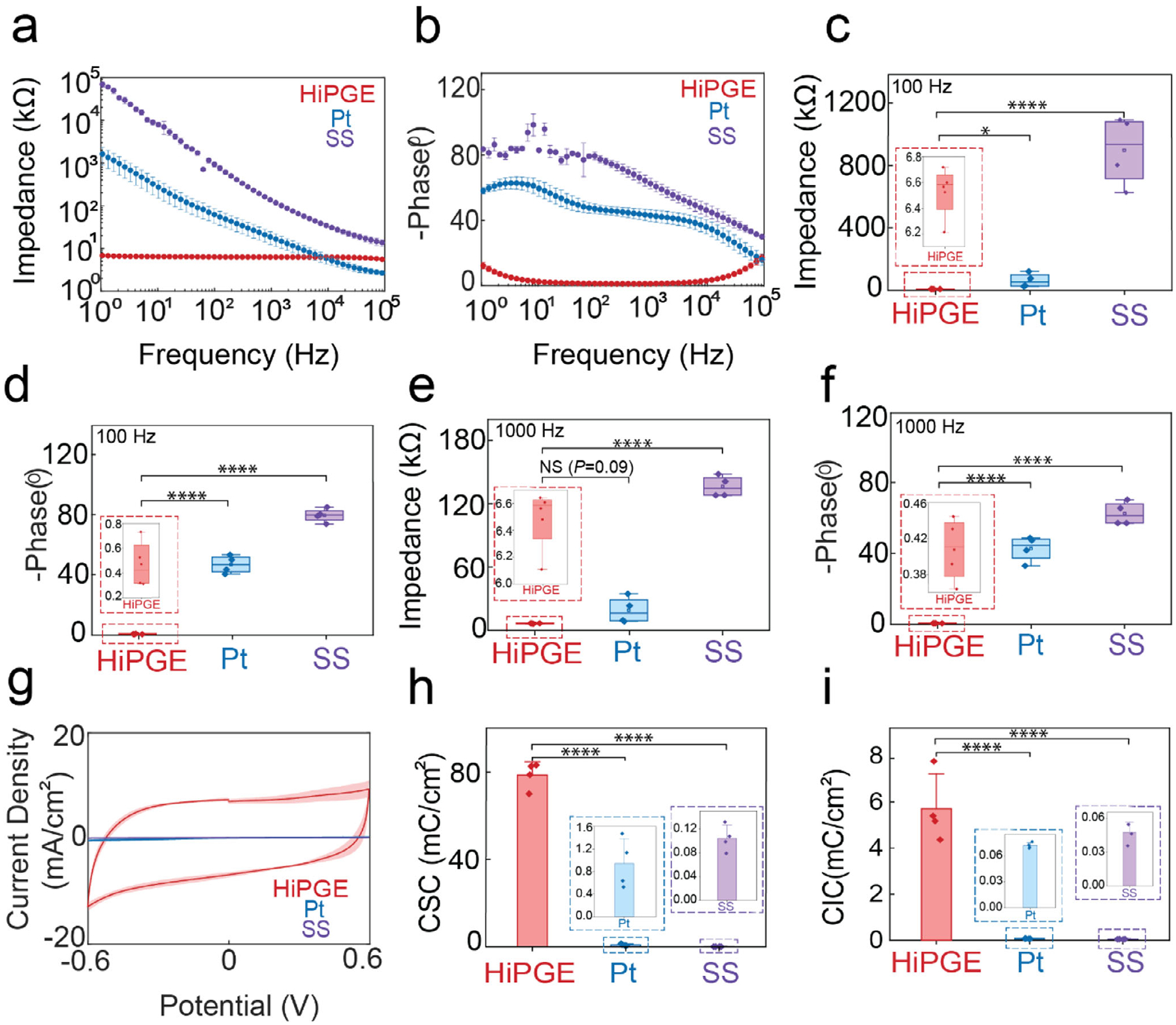
Electrical characterizations. (a,b) Electrochemical impedance spectroscopy (EIS) of HiPGE, Platinum (Pt) electrode, and Stainless Steel (SS) electrode. Values represent mean and standard errors of the mean (SEM) for *n* = 4 independent samples. (c–f) Impedance (kΩ) (c) and phase (°) (d) at 100 Hz; Impedance (kΩ) (e) and phase (°) (f) at 1000 Hz. The 25th and 75th percentiles are displayed by the box limits, center lines indicate the mean, and whiskers exhibit the fifth and 95th percentiles. Data points (mean and SEM) are shown for *n* = 4 independent samples. (g) Current density (mA/cm^2^) vs. potential (V) plots for HiPGE, Pt, and SS. Values represent the mean and SEM for *n* = 4 independent samples. (h,i) Charge storage capacity (CSC) (h), and charge injection capacity (CIC) (i) for HiPGE, Pt, and SS. In bar plots, values represent the mean and SEM for *n* = 4 independent samples. Statistical significance and *p* values are determined by unpaired two-sample *t*-test; NS, not significant; *P < 0.05 (in e, *p* = 0.09); *P < 0.05 (in c, p = 0.04); ****p < 0.0001 (in c–f, and h–i).

The electrical impedance of biological tissues plays a crucial role in providing information on cellular response [49], emphasizing the significance of assessing the impedance of neural devices. Electrochemical impedance spectroscopy (EIS) was applied to study the interfacial impedances of HiPGE in PBS, mimicking physiologically relevant environments. The impedance characterization of HiPGE was compared to control electrodes—platinum (Pt) electrode and a stainless steel (SS) electrode. The results demonstrated that the impedance of HiPGE was below 10 kΏ for all the tested frequencies from 1 to 100 000 Hz, whereas the impedance for Pt and SS was up to three or four magnitudes higher than HiPGE, as illustrated in Figure 4a and Figure S15a–c. It also clearly showed that the phase angles for HiPGE were close to zero, while phase angles for Pt and SS were much higher than HiPGE (Figure 4b; Figure S15d–f). The impedance and phase angles of each electrode were also evaluated at frequencies of 100 Hz and 1000 Hz, respectively. The results illustrated that the impedance values of HiPGEs were below 10 kΏ, and phase angles were close to zero. In contrast, the impedance and phase angles for the control electrodes were much higher than HiPGEs, as depicted in Figure 4c–f. The superior electrochemical performance of HiPGE can be attributed to its conducting polymer hydrogel electrode composition, which supports both ionic and electronic charge transport. This dual-conduction mechanism effectively bridges the gap between the electron-based signaling of electronics and the ion-based signaling of biological systems, therefore significantly reducing interfacial impedances [43, 50]. In addition, unlike metal electrodes that provide a 2D electrode interface, conducting polymer hydrogel possesses a 3D, porous structure that can dramatically increase the effective interfacial area available for charge transfer at the electrode to reduce the interfacial impedance.

For further investigation of the electrochemical properties of HiPGEs, cyclic voltammetry (CV) was conducted and compared to the control electrodes, as shown in Figure 4g and Figure S15g–i. The charge storage capacity (CSC) and the charge injection capacity (CIC) for HiPGE are both superior to the control electrodes (Figure 4h–i). This enhanced CIC originates from the conducting polymer hydrogel component in HiPGE, which supports both ionic and electronic charge transport. The hydrated hydrogel network facilitates high ionic mobility and efficient coupling with the ionic environment of neural tissue, while the *π*-conjugated polymer backbone ensures rapid electron mobility within the electrode [51, 52]. This allows the simultaneous movement of electronic charges along the conjugated skeleton and ionic charges at the interface. Unlike metal electrodes that only support double-layer capacitance at the interface, the porous conducting polymer hydrogel enables mobile ion penetration at the interface, which allows a much larger effective interface with significantly higher double-layer capacitance (volumetric capacitance) for charge injection [53]. In addition to double-layer capacitance for charge injection, conductive polymer hydrogel also supports pseudocapacitive behavior from redox reactions that occur on the conducting polymer chains, further increasing the total charge injection capacity [54].

Moreover, the electrodes were immersed in PBS for 28 days at 50°C to evaluate their stability under accelerated aging conditions. While 37°C typically mimics physiological in vivo temperatures, the elevated temperature of 50°C was used to simulate accelerated aging and degradation characteristics [55, 56]. The results showed that HiPGEs demonstrated stable properties for 28 days of incubation at 50°C in PBS (equivalent to 69 days at physiological temperature, Figure S16). The likely degradation mode of the electrode material could come from chain scission resulting from the hydrolysis of the ester in the polyurethane component of the hydrogel material. This could result in gradual softening of the electrode material, which could be mitigated by crosslinking of the bicontinuous structure in the conducting polymer hydrogel material, if needed [48].

To further evaluate HiPGE’s electrochemical stability under more physiologically relevant conditions, additional characterization was performed in artificial cerebrospinal fluid (aCSF). Using procedures analogous to those conducted in PBS, electrochemical impedance spectroscopy revealed that HiPGE maintained impedances below 10 kΩ across the tested frequency range (1–100 000 Hz) during a 28-day stability study in aCSF at 37°C under dynamic agitation (60 rpm, ~1 Hz), a standard in vitro condition used to mimic physiological motion [57, 58]. Impedance values remained stable throughout the testing period (Figure S17). Furthermore, charge storage capacity (CSC) and charge injection capacity (CIC) were measured under the same dynamic aCSF conditions and also remained consistent over 28 days, demonstrating robust electrochemical performance (Figure S18).

## 6 | HiPGE In Vivo Functionality Assessment

**FIGURE 5 |:**
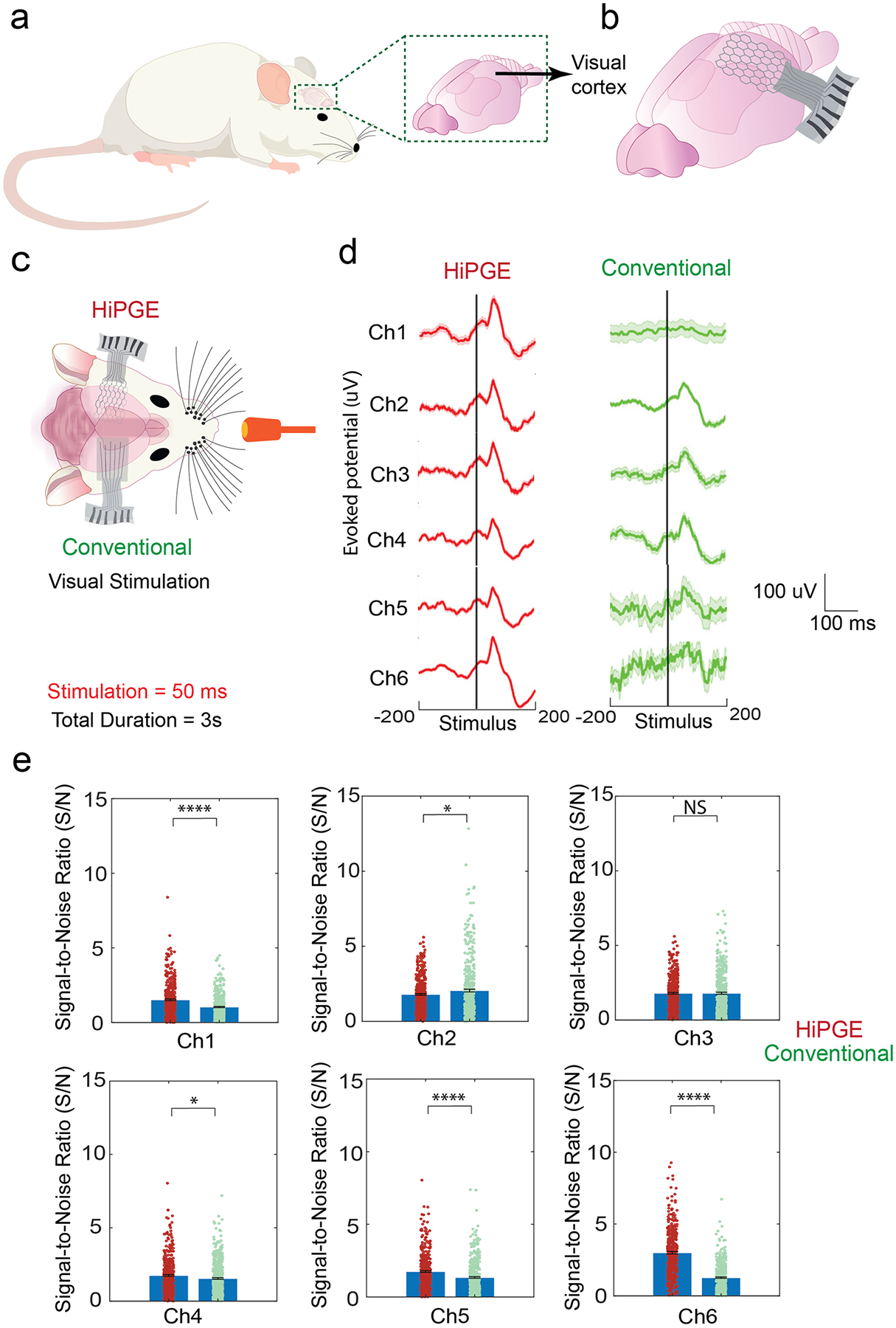
Implantation of personalized HiPGE on a rat’s cortex for recording. (a) Schematic illustration of a rat and its visual cortex. (b) Schematic illustration of the implantation of HiPGE on the visual cortex of a rat’s brain. (c) Schematic illustration of the intra-animal experimental design, showing bilateral implantation of HiPGE (left hemisphere) and conventional electrodes (right hemisphere) over the visual cortex. A controlled visual stimulus (50-ms light flash) was delivered to evoke cortical responses, enabling direct within-subject comparison of recording performance between the two electrode types. (d) Visual evoked potentials (VEPs) recorded from HiPGE and conventional electrodes in awake rats (*n* = 3). Trial-averaged waveforms (433 trials for conventional electrode and 420 trials for HiPGE) are shown for all six channels across animals. The dark traces represent the mean response, and the light-colored shaded regions indicate the standard errors of mean (SEM). (e) Signal-to-noise ratio (SNR) quantification across all animals (*n* = 3), using 433 trials for the conventional electrodes and 420 trials for the HiPGE. Scatter plots show individual trial values. Bars indicate mean ± SEM. Statistical comparisons between the two electrode types were performed using a two-sample t-test. Significance is denoted as *p < 0.05, **p < 0.01, ***p < 0.001, and ****p < 0.0001.

To validate the functional capacity of the interface of HiPGE, the device was implanted in multiple rats to demonstrate that the fabricated HiPGE can conform to the cortical surface of the brain as well as effectively record the ECoG signal, which is designated as ROI, of the cortex (Figure 5a,b). Rats were selected for recording purposes, followed by making personalized designs and fabricating the devices based on the brain size and shape of different rats (Figures S19–S21). To evaluate the functional performance of the personalized HiPGEs in vivo, visual evoked potentials (VEPs) were recorded from awake rats. For intra-animal comparison (Figure 5c), a conventional electrode array (Figure S22; see Methods for details) was implanted on the contralateral visual cortex, such that each rat carried both a personalized HiPGE (left side) and a conventional electrode (right side). Across all animals, 420 trials from the HiPGE and 433 trials were collected from the conventional arrays. The personalized HiPGEs consistently exhibited superior evoked-potential recordings, particularly in channels located near the edges (Figure 5d; Figure S23). This improvement is attributed to the enhanced conformability and softness of the HiPGE design, which enables more intimate and stable contact with the cortical surface, thereby yielding higher-quality neural signals.

Moreover, to quantitatively compare the recording performance of the HiPGE and conventional electrodes, peak latencies (Figure S24), amplitude variance (Figure S25), and signal-to-noise ratio (SNR; Figure 5e) were analyzed. The peak latency and amplitude variance showed no significant differences between the two electrode types, indicating comparable waveform consistency as assessed by two-sample *t*-tests. In contrast, the SNR analysis revealed clear advantages for the HiPGEs. Notably, electrodes located near the edges exhibited significantly higher SNR values compared to their conventional counterparts (Channel 1, Channel 5, and Channel 6; ****p < 0.0001). On the other hand, middle channels exhibited either comparable or only modestly improved SNR. Channel 3 showed no significant difference, whereas channels 2 and 4 demonstrated moderate significance (*). This pattern is consistent with the expected mechanical behavior of the devices: the honeycomb-inspired architecture of the HiPGE renders it softer and more compliant, enabling more seamless conformity to the curved cortical surface. As a result, even the central recording sites maintain improved contact relative to the stiffer conventional electrodes, whose limited flexibility likely contributes to reduced performance in their middle channels. Collectively, these quantitative metrics further support the conclusion that the enhanced mechanical compliance of the personalized HiPGE design underlies its superior recording performance.

This improvement primarily results from the synergistic effects of reduced interfacial impedance and enhanced cortical conformity. The low interfacial impedance facilitates efficient ionic-electronic coupling and charge transfer at the electrode-tissue interface, while the highly conformable honeycomb architecture ensures intimate contact with the cortical surface, enabling an improved interface compared to the conventional electrodes [50, 59].

## 7 | Multimodal Evaluation of Morphological Integrity and Tissue Response

**FIGURE 6 |:**
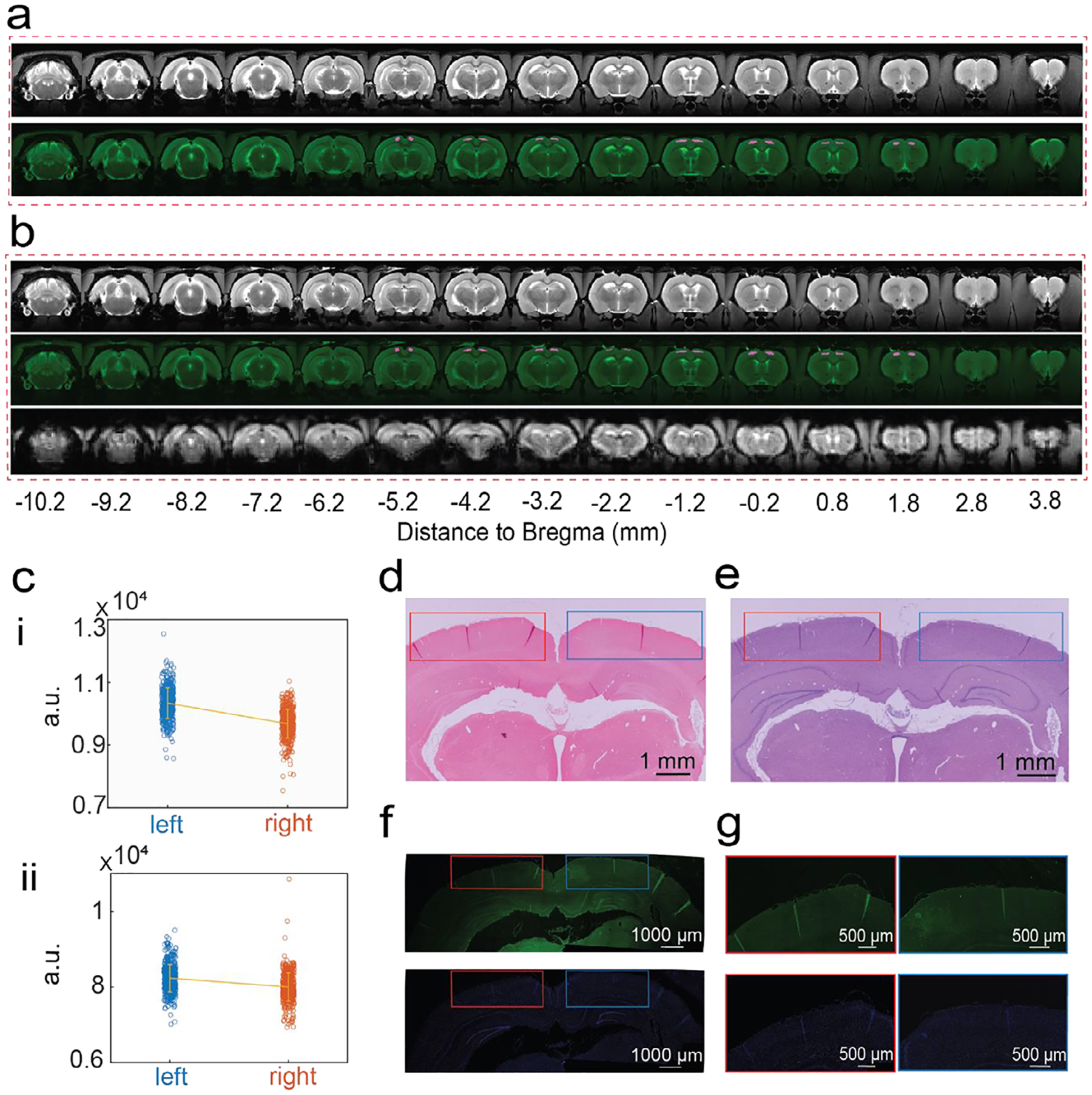
MRI scanning and in vivo biocompatibility of a rat’s brain with and without HiPGE. (a,b) Structural and functional images of Rat#1 brain without HiPGE (before surgery) (a) and with HiPGE (after surgery) (b) on the surface of the left SM cortex. The SM cortical regions under HiPGE were manually selected in a mirrored configuration relative to the right hemisphere in structural images, which were marked as pink in the second row of panels (a,b). (c) Signal intensity of voxels in left SM cortex and right SM cortex without (i) and with HiPGE (ii). (d–g) Representative histological images of a rat’s brain 4 weeks post-implantation. Masson’s Trichrome (d), Hematoxylin and Eosin (e), Iba-1 (green, f), and DAPI staining (blue, f), and zoom-in view of the implanted and non-implanted regions (g). Red and blue boxes refer to non-implanted and implanted regions, respectively.

To comprehensively evaluate the effects of HiPGE on the cortical surface before and after implantation, its MRI compatibility was initially assessed using a phantom test (Figure S26). Following the confirmation of MRI compatibility, structural and functional MRI (fMRI) scans were collected from rats pre- and post-implantation of HiPGE utilizing a 7T scanner (details in Methods), as illustrated in Figure 6a,b. No significant distortion or signal loss was observed surrounding the electrode in either structural or functional MRI images. Furthermore, to validate that the signal intensity was not impacted by the electrode, SM cortical regions under the electrode were manually selected in a mirrored configuration relative to the right hemisphere for all structural images. The signal intensity of voxels within these two regions was plotted for the rat before and after electrode implantation, as shown in Figure 6c. The results demonstrated that the difference in signal intensity between the left and right hemispheres was comparable across genders and remained consistent before and after HiPGE implantation.

To assess the long-term effects of HiPGE implantation on cortical morphology and tissue responses, rat brain tissue from implanted (HiPGE) and non-implanted rat brain regions was harvested and evaluated at 4 (Figure 6d–g; Figure S27) weeks post-surgery for four rats. Masson’s trichrome staining was employed for precise evaluation of fibrotic scar tissue formation, and no collagen fiber deposition was observed in the implanted regions, resembling the non-implanted control (Figure 6d; Figures S27a,d,g and S28). For quantitative analysis, the normalized intensity of the collagen fiber component was analyzed using MATLAB. The regions within 100 μm from the probe boundary were selected on both implanted and non-implanted sides for comparison. A two-sample *t*-test indicated no significant difference (*p* > 0.05) in collagen fiber intensity between the two regions. Furthermore, Hematoxylin and Eosin (H&E) staining of sectioned brain slices revealed no significant morphological differences between the implanted and non-implanted regions (Figure 6e; Figures S27b,e,i and S29). Similar quantitative analysis of the normalized eosin intensity, representing cytoplasmic and extracellular protein distribution, was performed for both sides. Statistical comparison showed no significant difference (*p* > 0.05) in staining intensity, indicating that the implant did not cause noticeable structural disorganization or tissue protein loss around the implantation site.

Additionally, brain slices were labeled with Iba-1, a microglial marker, to further investigate neuroinflammation and glial scar formation. The fluorescent images (Figure 6f,g; Figures S27c,f,j and S30) demonstrated comparable microglial signals for both the HiPGE-implanted brain and the control brain, where nothing was implanted, indicating no obvious immune response to the implanted HiPGE. Similar quantitative analysis of normalized Iba-1 fluorescence intensity revealed no significant difference (*p* > 0.05) between the two regions. Collectively, these results demonstrate that HiPGE achieves seamless integration with minimal tissue response, preserves cortical architecture, and avoids adverse effects at 4 weeks post-implantation across multiple animals.

## 8 | Conclusion

HiPGE represents a transformative leap in neural interface technology, combining a biomimetic honeycomb architecture with patient-specific 3D printing to overcome noise and signal fidelity challenges in neural recording. Using MRI-compatible, low-impendance hydrogels with tunable mechanical properties, the platform enables precise fabrication of microscale electrodes that conform seamlessly to the brain’s gyral folds. This tailored design ensures robust electrode-tissue integration, minimizing mechanical mismatch and improving signal fidelity during in vivo neural activity recording. Chronic histology studies over 4 weeks post-implantation confirmed HiPGE’s biocompatibility, revealing no significant immune response or structural disruption to brain tissue. Furthermore, 3D printability of the device significantly reduces production costs, increasing access to personalized neural implants across diverse groups. By synergizing patient-specific customization with scalable manufacturing, HiPGE pioneers a new era of adaptive neural interface technologies, advancing brain-computer interfaces, neuroprosthetics, and clinical neuromodulation therapies. This innovation sets a blueprint for next-generation neural interfaces that prioritize precision, safety, and accessibility.

## 9 | Experimental Section

### 9.1 | Materials

For the preparation of the bi-continuous conducting polymer hydrogel material for electrodes, poly(3,4-ethylenedioxythiophene):polystyrene sulfonate (PEDOT:PSS) (Agfa Corporation), ethanol (Sigma–Aldrich), dimethyl sulfoxide (DMSO; Sigma–Aldrich), and hydrophilic polyurethane (AdvanSource Biomaterials) were used. For the preparation of the encapsulation layers of the designed devices, PDMS (SYLGARD 186, Dow Corning) was used. For device fabrication, 5-mL syringe barrels and 200 and 100 μm nozzles were used (Nordson EFD). For demonstration purposes, a 3D printer (FDM technology, Creality CR Series) and CR-PLA as a filament (Shenzhen Creality 3D Technology Co., LTAD.) were used for manufacturing human brain models.

### 9.2 | 3D Brain Model Reconstruction

For patient-specific brain models, all MRI data were downloaded from the Open Access Series of Imaging Studies (OASIS, www.oasis-brains.org) Website [60]. A total of 21 patient data were selected (Detailed Information is given in Tables S1 and S2), and 3D brain models were reconstructed using the free and open source software 3D Slicer (Version 5.6.2, www.slicer.org) [61]. In 3D Slicer, after brain construction, the ROI SM cortex areas were selected to determine the dimensions for the personalized HiPGE devices for specific individual patients.

### 9.3 | Fabrication of 3D Printed Brain Models

For printing the human brain models, high-print quality, non-toxic, and reliable PLA (polylactic acid) filament was used in the FDM 3D printer. After the reconstruction of the 3D model in STL format, the 3D model was imported into the Creality Slicer software (Version 4.8) to slice the model. During the slicing of the model, the layer height was selected to be 0.16 mm, and the infill density was chosen to be 20% with a gyroid pattern. The build plate adhesion type was brim. To give the support structure of the models, a tree structure was selected with 60° support overhang angle everywhere. After that, the model was exported into gcode format to load the file in the FDM printer (Creality). The temperature of the printer’s build plate is 60°C, and the 0.4 mm diameter nozzle’s temperature is 200°C.

### 9.4 | Ink Preparation

To prepare the encapsulation ink, parts A and B (10:1 ratio) of PDMS were mixed by a centrifugal mixer (AR-100, Thinky). The mixed solution was then transferred into a 5-mL syringe, followed by a thorough mixer. For conductive ink, 6 w/v% PEDOT: PSS was dissolved in a mixture of deionized water and DMSO (DI water: DMSO = 85:15 v/v) and filtered with a syringe filter. To prepare 10 w/v% polyurethane (PU), 20 w/v% polyurethane in ethanol solution (ethanol:DI water = 95:5 v/v) was used. 10 w/v% PU and 6 w/v% PEDOT: PSS were then thoroughly mixed so that the PEDOT: PSS to PU ratio is 1:3, followed by filtering. The ink was ready to print after the final stage of transferring the combined solution into a barrel for 3D printing and thoroughly mixing it in the mixer.

### 9.5 | Finite Element Modeling

To evaluate the mechanical properties of HiPGE, finite element simulations were carried out by the software ABAQUS. The HiPGE was reconstructed in modeling, based on the experimental fabrication of the patient 39 device (Figure S7). Since the electrode layer contributes little to the mechanical response of the device, only the encapsulation layer was modeled in FEM. The PDMS, SBS, and metal control devices share the same trapezoidal geometrical setting but feature a space-filled plate rather than a hexagonally porous structure. The PDMS layer was modeled as a Neo-Hookean hyperelastic material with Young’s modulus and Poisson’s ratio as 1.8 MPa and 0.48 [31]. For SBS and metal, the Young’s modulus was set to be 45 MPa and 60 GPa, and Poisson’s ratio to be 0.48 and 0.42 [45]. To calculate bending stiffness, a fixed boundary condition was applied at one end of the device, and a small vertical displacement *d* was given on the other. The external work (*W*) required to bend the device was then calculated. The effective bending stiffness per width of the device can then be estimated as *D* = 2*Wl*^3^/3*d*^2^*b*, where *l*, *b* are the length and width of the device, respectively [62, 63]. For the conformability test, the brain tissue was cut from SM cortical regions of an MRI-reconstructed 3D brain model, with a Young’s modulus of 6 kPa and a Poisson’s ratio of 0.48 [64]. Viscoelastic effects were not included because: (1) the strain rate of the simulation was relatively small (0.1–0.3 s^−1^); and (2) the viscosity of brain tissue (1–10 Pa·s) [65] would generate stresses of only 0.1–3 Pa, which are negligible compared with the elastic response (~10 kPa). The device was initially set to suspend 5 mm above the brain surface and gradually descend because of gravity. The bottom of the brain tissue is fixed, and the surface interaction between the device and the brain tissue was set to be adhesive and inseparable.

The in silico modeling was carried out by COMSOL Multiphysics to measure the connectivity rate. The geometry setting of the device-brain assembly was inherited from the results of the conformality test. Four locations of electrodes on the HiPGE were manually picked from the proximal region of the brain surface (distance within 0.5 mm, Figure S3). In PMDS and SBS control devices’ assembly, the same four locations of electrodes were selected as HiPGE-brain assembly (Figure S4). In PDMS and SBS matrix devices, the electrodes were set to be matrix-aligned, as conventional devices designed [45], with 12 electrodes in total (Figure S5). A constant unit electrical potential was applied to the surface of brain tissue, and all the domains except the electrodes on the devices were set to be insulators. The electrodes were set to be conductors with high conductivity. The number of connected electrodes was counted to calculate the connectivity rate percentage.

### 9.6 | Device Fabrication With 3D Printing Technology

The designs of the three layers of the HiPGE were generated by computer-aided design (CAD), followed by exporting them in DXF format. Next, the DXF format was converted into the codes to control the ink printing in the x-y-z direction by the built-in DXF conversion package. As a substrate of the fabrication process, glass plates were used and treated with Rain-X water repellent, followed by printing of the PDMS ink. A 5-mL syringe barrel containing ink was connected to the UlitmusPlus dispenser for applying pressure during printing to control the on and off of the dispenser, which allows the ink to print the designed paths accordingly. The encapsulation layers were printed with PDMS ink in a 5-mL syringe barrel, followed by curing at 125°C for 15 min. For the encapsulation layers, printing was performed at a speed of 3.5 mm/s under an applied pressure of 100 psi, whereas the conductive layers were printed at a speed of 9 mm/s with a pressure of 25 psi, with a printing resolution of 100 μm. The rheological properties of both inks are demonstrated in Figure S9.

### 9.7 | Electrical Characterization

For the electrical characterization, the device electrode part (exposed area: 200 μm × 100 μm), the Platinum counter electrode, and the Ag/AgCl reference electrode were immersed in phosphate-buffered saline (PBS, as an electrolyte). Electro-chemical impedance spectroscopy (EIS) was performed using a potentiostat with a frequency analyzer (Autolab PGSTAT204, Metrohm). The frequency range of 1–100 kHz was scanned with an amplitude of 0.01 V.

Cyclic voltammetry (CV) measurements were conducted using a potentiostat (Autolab PGSTAT204, Metrohm) with the potential of ± 0.6 V (scan rate 0.1 V/s). After that, charge storage capacity (CSC) was calculated from the data of CV measurements using a customized MATLAB code from the formula, $\int_{E2}^{E1}i(E)/(2vA)$, where E1 and E2 were the range of potential window, i, the current is a function of potential and was taken for each potential value, and *ν* and A were considered as scan rate and area of the device, respectively. To measure the charge injection capacity (CIC) of the device, Chronopotentiometry fast was performed using galvanostat mode (Autolab PGSTAT204, Metrohm). The current pulses were progressively amplified until the electrode under examination polarized to an extent marginally higher than the water electrolysis’s voltage range of −0.6 to 0.8 V. Ohmic resistance in the circuit generated instantaneous polarization of the electrodes, which was subtracted to adjust the voltage traces [64]. The measured output voltage and current were used to calculate the CIC of the device as Q(c) + Q(a)/A (where Q(c) and Q(a) are the total injected current in the cathodal and anodal phase, respectively, and A is the area of the device). To benchmark the electrochemical performance of HiPGE, identical characterization experiments were conducted using platinum control electrodes (radius: 65 μm, exposed length: 0.2 cm) and stainless-steel control electrodes (radius: 50.8 μm, exposed length: 0.4 cm).

### 9.8 | Mechanical Characterization

Uniaxial tensile tests were performed using a mechanical testing machine (CellScale) to evaluate the stress-strain behavior of the devices. Devices were stretched at a constant rate of 1 mm/s until rupture. Young’s modulus was determined from the initial slope of the stress–strain curve, and ultimate strain was defined as the strain at failure. For quantitative conformity analysis, cortical surface coverage was defined as the percentage of electrode sites in direct contact with the cortical surface, calculated as (number of electrode sites contacting the cortex / total number of electrode sites) × 100%.

### 9.9 | Conventional Electrode Fabrication

For comparing the functional performance of the personalized HiPGE with a conventional electrode, the electrode designs were tailored to match the size of a rat’s brain. To replicate the structure of conventional electrodes (Figure S22), the fabrication included a bottom layer produced with a 500 μm nozzle, a middle layer with a 100 μm nozzle, and a top layer with a 200 μm nozzle. While the same materials were used for printing both designs, the overall stiffness of the replicative electrodes was significantly higher than the HiPGE due to the thickness of the layers.

### 9.10 | Animals

To test the functional performance of HiPGE, four adult male Long-Evans rats (400–650 g) and three adult female Long-Evans rats (240–260 g) were used in this study. Animals were housed in Plexiglas cages with ad libitum access to water and food. The animal room was under a 12-h light: 12-h dark cycle, and the temperature was maintained at 22°C–24°C. All experiments were approved by the Pennsylvania State University Institutional Animal Care and Use Committee (IACUC).

### 9.11 | Phantom Test

The animal was anesthetized with a cocktail of ketamine (33 mg/kg) and xylazine (13 mg/kg) and perfused first with saline, then with 4% PFA (Thermo Scientific, Catalog No. J61899.AP). The brain was then taken out carefully and fixed in 4% PFA with 30% sucrose for one day. After fixation, the electrode was placed on the surface of the left SM cortex and fixed with a tissue adhesive (Vetabond, 3 M, St. Paul, MN). Then, the brain was placed in a plastic tube with 2% w/v agarose. For the preparation of 2% w/v agarose, 2 g agarose (Invitrogen, Catalog No. 16500–500) was mixed with 100 mL of water, and then the solution was heated to a boil in a microwave oven. After that, it was kept in a plastic tube and cooled down at room temperature. Then, the brain with HiPGE was placed on the surface of agarose, and then the tube was filled with saline. A structural MRI of the phantom was performed on a 7T Bruker 70/30 BioSpec scanner through ParaVision 6.0.1 software (Bruker, Billerica, MA) at the high-field MRI facility at the Pennsylvania State University. Structural data were collected using a rapid imaging with refocused echoes (RARE) sequence with the following parameters: echo time (TE) = 40 ms; repetition time (TR) = 3000 ms; in-plane resolution = 0.125 × 0.125 mm^2^; slice thickness = 1 mm; number of slices = 20; FOV = 32 × 32 mm^2^; image matrix = 256 × 256.

### 9.12 | MRI Scanning

Before and after surgery, anatomical images were recorded using a rapid imaging with refocused echoes (RARE) sequence with the following parameters: echo time (TE) = 40 ms; repetition time (TR) = 3000 ms; in-plane resolution = 0.125 × 0.125 mm^2^; slice thickness = 1 mm; number of slices = 20; FOV = 32 × 32 mm^2^; image matrix = 256 × 256. Moreover, fMRI data were also collected after surgery using a T2*-weighted gradient-echo echo-planar-imaging (EPI) sequence with the following parameters: echo time (TE) = 15 ms; repetition time (TR) = 1000 ms; in-plane resolution = 0.5 × 0.5 mm^2^; slice thickness = 1 mm; number of slices = 20; FOV = 32 × 32 mm^2^; image matrix = 64 × 64. All MRI scans were aligned to a rat brain template for consistent visualization. For structural scans, the sensorimotor (SM) cortical regions under the electrode in the left hemisphere were manually selected in a mirrored configuration relative to the corresponding regions in the right hemisphere. The BOLD intensity of voxels in the regions under the electrode was then compared to the BOLD intensity of the mirrored brain regions in the right hemisphere. This comparison was conducted before and after surgery across both male and female rats.

### 9.13 | In Vivo Surgery and Recording

For the implantation of HiPGE and conventional electrode, anesthesia was initiated with isoflurane, followed by the intramuscular administration of a ketamine-xylazine cocktail (40 and 12 mg/kg, respectively) and a subcutaneous injection of buprenorphine (1.0 mg/kg). Throughout the surgery, a gas mixture of oxygen and isoflurane (0%–2%) was provided constantly through a nose cone to maintain the anesthetic state and blood oxygenation level, and the body temperature was maintained at 37°C via a warming pad (PhysioSuite, Kent Scientific Corporation). Oxygen saturation (SpO_2_) and heart rate were continuously monitored and recorded every 20 min via a pulse oximetry (MouseSTAT Jr, Kent Scientific Corporation). During the surgery, the animals were immobilized on a stereotaxic platform (David Kopf Instruments, Tujunga, CA). Craniotomies were made over the left and right visual cortex area separately (coordinates: anterior: −3, posterior −9; medial: −0.5, lateral: −3.7), and dura mater was carefully removed after once exposed. Personalized HiPGEs (each electrode: 200 × 100 μm, with 100 μm center-to-center spacing of 0.8 mm on AP direction and 1 mm on ML direction) were gently placed on the surface of the left visual cortices through the window. In a similar way, the conventional electrodes were implanted over the corresponding regions of the right hemisphere. Specifically, the electrodes consisted of six recording sites arranged in a 2 × 3 grid. Inter-site distances were 1.0 mm along the mediolateral (ML) direction and 0.8 mm along the anteroposterior (AP) direction, corresponding to approximately 4 mm of cortical surface coverage over V1/V2 along the AP axis. The stereotaxic coordinates for the six recording sites were defined relative to bregma along the mediolateral (ML) and anteroposterior (AP) axes. For example, for rat#2, the HiPGE electrode placed in the left hemisphere, the six sites were located at ML/AP coordinates of −2.5/−9.0 mm (Ch1, primary visual cortex (V1)), −3.5/−8.2 mm (Ch2, V1), −2.5/−7.4 mm (Ch3, V1), −3.5/−6.6 mm (Ch4, V1), −2.5/−5.8 mm (Ch5, secondary visual cortex (V2)), and −3.5/−5.0 mm (Ch6, V2), spanning primary and secondary visual cortical areas. For the conventional electrode placed in the right hemisphere, the corresponding sites were positioned at +2.5/−9.0 mm (Ch1, V1), +3.5/−8.2 mm (Ch2, V1), +2.5/−7.4 mm (Ch3, V1), +3.5/−6.6 mm (Ch4, V1), +2.5/−5.8 mm (Ch5, V2), and +3.5/−5.0 mm (Ch6, V2). The grounding and reference wires of the electrode were twisted with a silver wire fixed on the surface of the right cerebellum. the surface of the cortex was fixed using a tissue adhesive (Vetabond, 3 M, St. Paul, MN) and dental cement (ParaBond, COLTENE, Cuyahoga Falls, OH). After surgery, 7 days or more were allowed for rats to recover. To enable intra-animal comparisons between HiPGEs and conventional electrodes, electrophysiology recordings were performed during visual stimulation under awake conditions. Before recording, animals were briefly anesthetized with isoflurane, then fixed in a restrainer and allowed to fully recover before stimulation. A blue laser system (473TB-300FC, Shanghai Laser & Optics Century Co., Ltd) was calibrated to an output power of 150 μW and positioned 2–3 cm from the eyes. Dim illumination was used to minimize irritation. Each trial lasted 3 s, with the laser on for 50 ms and off for the remainder. TTL pulses generated through LabVIEW synchronized the laser with the ECoG recording system (Tucker Davis Technologies (TDT) Inc, Alachua, FL). After completion of the recording session, animals were euthanized using CO_2_ in accordance with approved animal care protocols.

### 9.14 | Data Analysis

The recorded data were analyzed using custom MATLAB codes (MATLAB 2021b). During the preprocessing, the motion-induced artifacts were first removed from the raw electrophysiology signal using a threshold = mean of the data ± 3* standard deviation of the data. After that, 60 and 8 Hz notch filters were applied to the data to remove power supply noise and heartbeat-induced noise. Then the data was filtered from 0.1 to 200 Hz to get LFP. To find peaks of VEP, we first averaged electrophysiology signal across trials from each rat, then the maximum value and minimum value of the signal in the time interval of 0–200 ms after stimulus were found and defined as positive peak (P1) and negative peak (N1). Peak latencies were defined as the duration between the onset of stimulus and the two peaks. Amplitude variance was calculated as the variance of peak amplitude across all trials for each rat. The signal-to-noise ratio (SNR) was calculated using the formula, SNR = (S/N) [66], where ‘S’ represents the peak(P1)-to-peak(N1) signal amplitude of evoked potential during the stimulation period and ‘N’ is the standard deviation of the signal during the baseline resting period (−1.5–0.5 s before stimulus) for each trial [66–68].

### 9.15 | Histology

After 4 weeks of implantation, the rat was anesthetized with a cocktail of ketamine and xylazine (33 and 13 mg/kg) and perfused transcardially with saline, followed by a mixed solution containing 4% paraformaldehyde (PFA) and 10% sucrose to ensure thorough fixation. To preserve the brain tissue and minimize structural damage, the brain was taken out carefully and immersed in 4% PFA for 48 h. Subsequently, the fixed brain was processed using the Tissue-Tek VIP 6 AI Vacuum Infiltration Processor for automated tissue preparation. The processing cycle lasted 16 h, after which the brain tissue was embedded with evenly distributed paraffin wax using the Tissue-Tek TEC embedding system. The paraffin block was then cooled on a cold plate to solidify. Then, the brain was sectioned into 5 μm-thick slices using the Tissue-Tek Automated Microtome. The sections underwent deparaffinization, followed by hematoxylin and eosin (H&E) staining using the Leica Autostainer. For additional Immunohisto-chemistry staining, including Masson’s Trichrome and ionized calcium-binding adapter molecule (iba-1), the deparaffinized sections were collected from the autostainer for subsequent processing.

For Masson’s Trichrome staining, the deparaffinized brain sections were mordanted in Bouin’s solution for 1 h at 56°C, followed by washing under running water until the yellow coloration was completely removed and rinsing with deionized water. Subsequently, the sections were immersed in Weigert’s iron hematoxylin solution for 10 min, rinsed in deionized water, and stained with Biebrich scarlet-acid fuchsin solution for 2 min. The slices were then rinsed again with deionized water. Next, the sections were treated with a phosphomolybdic-phosphotungstic acid solution for 10 min, followed by staining in an aniline blue solution for 5 min. After another rinse with deionized water, the sections were treated with glacial acetic acid for 5 min. The samples were subsequently dehydrated through graded ethanol solutions (95% ethanol followed by absolute ethanol) and cleared in xylene with two changes for each step. Finally, the sections were mounted onto glass slides using Permount as the mounting medium.

For iba-1 staining, brain tissue sections were blocked using a blocking solution consisting of 0.3% Triton X-100 and 5% goat serum (Abcam) in 1× PBS for 1 h at room temperature. Slices were then incubated with the primary antibodies, rabbit anti-iba1 (1:400 dilution; FujiFilm Irvine Scientific) containing 0.3% Triton X-100 and 3% goat serum overnight at 4°C. After the incubation, slices were rinsed nine times over 30 min with 1× PBS. Slides were then incubated with the secondary antibodies, Alexa Fluor 488 goat anti-rabbit (1:200 dilution; Abcam) for 1 h at room temperature. Slices were then rinsed nine times over 30 min. They were then mounted on glass slides with coverslips using ProLong Gold Antifade Mountant with DAPI (Thermo Fisher Scientific). The slides were kept in the dark at room temperature for at least 24 h before imaging. Thereafter, the slices were imaged with a Leica SP8 Dive multiphoton microscope.

### 9.16 | Image Data Analysis

Microscopic images were processed using color deconvolution in Fiji to isolate specific staining components, including the collagen fiber (Masson’s Trichrome), eosin (H&E), and Iba-1 (immunofluorescence) channels. A custom MATLAB script was then used to quantify normalized staining intensity for each image. The probe boundary was defined manually using a polygon, and the distance of each pixel from the HiPGE interface was computed as its shortest distance to this boundary. Pixels were grouped according to their distance from the probe surface, with all pixels along a given polygon assigned the same distance value. Pixels within every 25 μm away from the probe boundary were grouped, and the intensity was normalized to the baseline intensity (500 μm away from the probe boundary). The average normalized intensity was then calculated for pixels located 100 μm away from the probe boundary. The same analytical procedure was applied to all images presented in Figures S26–S28. Bar plots were constructed to compare the mean ± SD normalized intensities between implanted and non-implanted regions.

### 9.17 | Statistical Analysis

OriginPro 2023 software and MATLAB (MATLAB 2021b) were used to assess the statistical significance of all comparison studies in this work. In the statistical analysis for comparison between multiple samples, one-way ANOVA was conducted with the threshold of *p < 0.05. Comparisons between two groups were conducted using two-sample *t*-tests, with significance defined as *p < 0.05. Significance levels are denoted as follows: *p < 0.05, **p < 0.01, ***p < 0.001, and ****p < 0.0001. Comparisons between two related groups were performed using two-sample *t*-tests, which revealed no statistically significant differences (*p* > 0.05).

## Supplementary Material

SI

Additional supporting information can be found online in the Supporting Information section.

**Supporting file**: adma72802-sup-0001-SuppMat.docx.

## Data Availability

The data that support the findings of this study are available from the corresponding author upon reasonable request.
