# Supplementary material for: 3D-Printable, Honeycomb-Inspired Tissue-Like Bioelectrodes for Patient-Specific Neural Interface
Source: Adv Mater. Author manuscript; Available in PMC 2026 Mar 26. (PMC13016203; doi:10.1002/adma.202516291)
Supplement: SI [file NIHMS2158131-supplement-SI.docx]

# **Supporting Information for**

**3D-Printable, Honeycomb-inspired Tissue-like Bioelectrodes for Patient-Specific Neural Interface**

*Marzia Momin^1,11^, Luyi Feng^1,11^, Xiaoai Chen^2,11^, Salahuddin Ahmed^1^, Basma AlMahmood^3^, Li-Pang Huang^4^, Jiashu Ren^1^, Xinyi Wang^5^, Hyunjin Lee^2^, Samuel R. Cramer^2^, Nanyin Zhang^2, 6,7,8^*, Sulin Zhang^1,2,9^*, Tao Zhou^1,2,6,7,10^**

^1^ Department of Engineering Science and Mechanics, The Pennsylvania State University, Pennsylvania 16802, USA.

^2^ Department of Biomedical Engineering, The Pennsylvania State University, Pennsylvania 16802, USA.

^3^ Department of Physics, The Pennsylvania State University, Pennsylvania 16802, USA.

^4^ Department of Biology, The Pennsylvania State University, Pennsylvania 16802, USA.

^5^ Department of Mechanical Engineering, The Pennsylvania State University, Pennsylvania 16802, USA.

^6^ Center for Neural Engineering, The Pennsylvania State University, Pennsylvania 16802, USA.

^7^ Huck Institutes of the Life Sciences, The Pennsylvania State University, Pennsylvania 16802, USA.

^8^ The Neuroscience Graduate Program, The Pennsylvania State University, Pennsylvania 16802, USA

^9^ Department of Materials Science and Engineering, The Pennsylvania State University, Pennsylvania 16802, USA

^10^ Materials Research Institute, The Pennsylvania State University, Pennsylvania 16802, USA.

^11^ These authors contributed equally: Marzia Momin; Luyi Feng; Xiaoai Chen.

* Corresponding authors. Emails: [nuz2@psu.edu](mailto:nuz2@psu.edu) (N. Zhang); [suz10@psu.edu](mailto:suz10@psu.edu) (S. Zhang); tzz5199@psu.edu (T. Zhou)

**
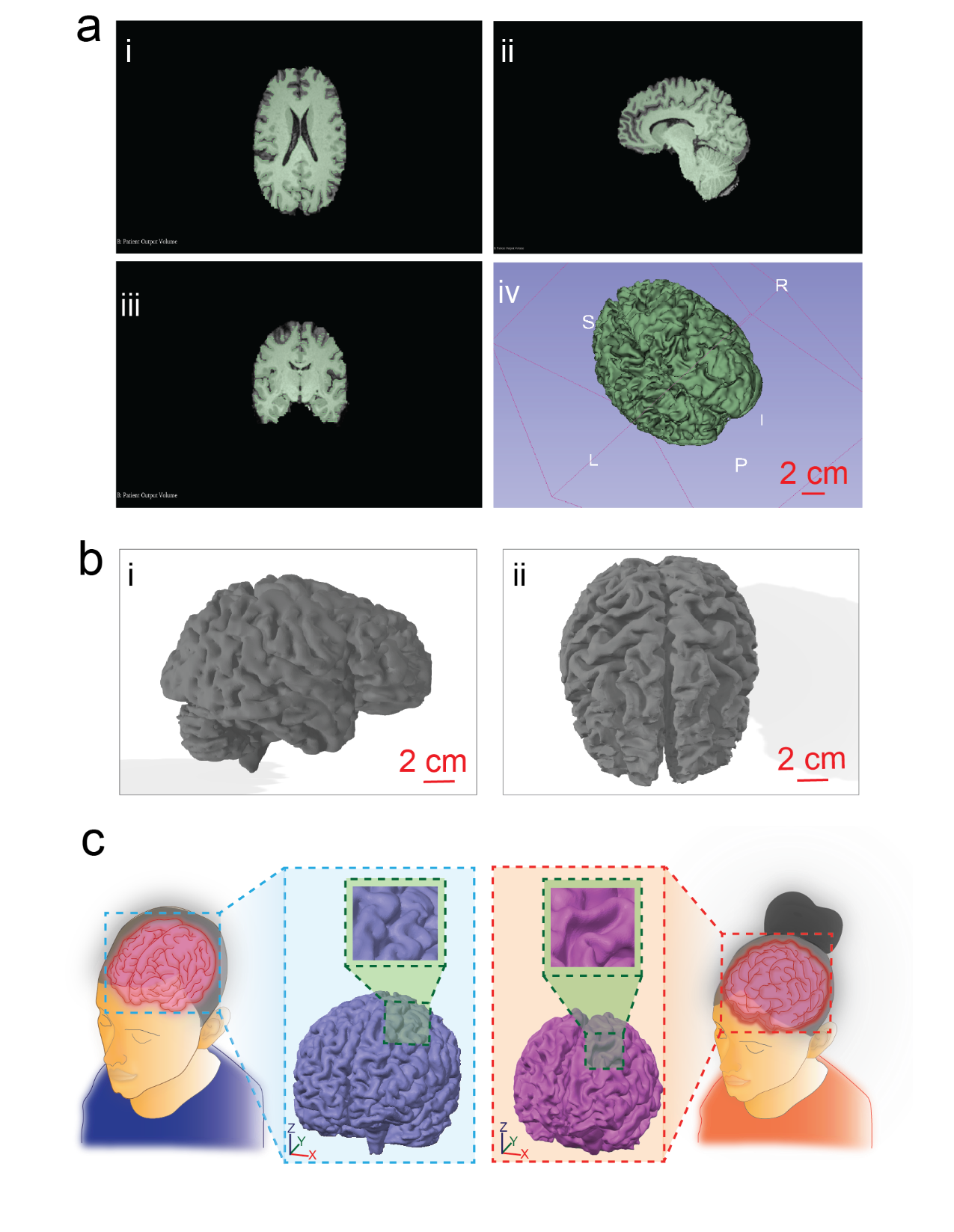
**

**Figure S1. Illustration and 3D model of brain reconstruction. a)** MRI structural images of the three planes - transverse (i), sagittal (ii), and frontal (iii), and reconstructed 3D model from the MRI structural images (iv) in 3D Slicer. **b)** Demonstration of 3D models, side view (i) and top view (ii). **c)** Illustration of the cortical surface and its zoom-in cross-sectional view of individuals (Patient 16 - right-handed and Patient 261 - left-handed).

**
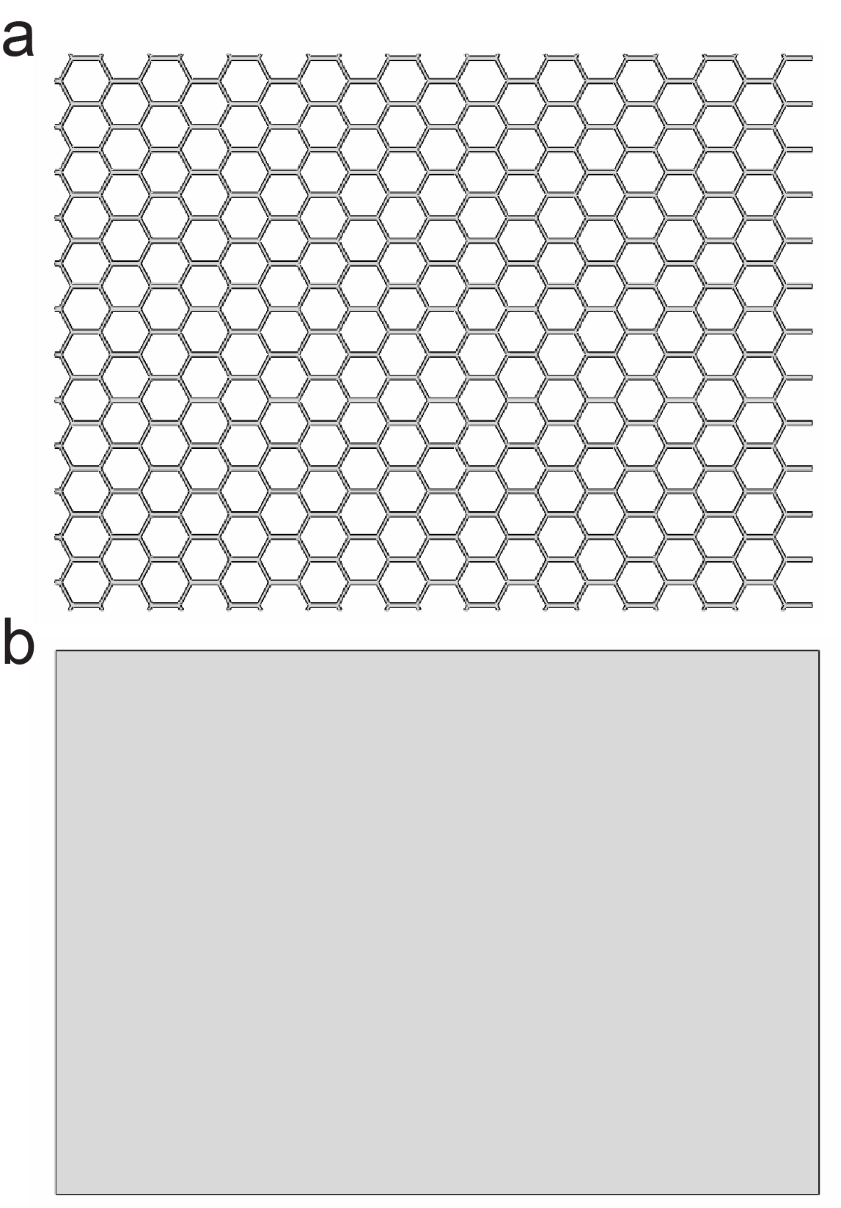
**

**Figure S2. Geometrical setting of HiPGE (a) and control device (b) for mechanics evaluation simulations.**

**
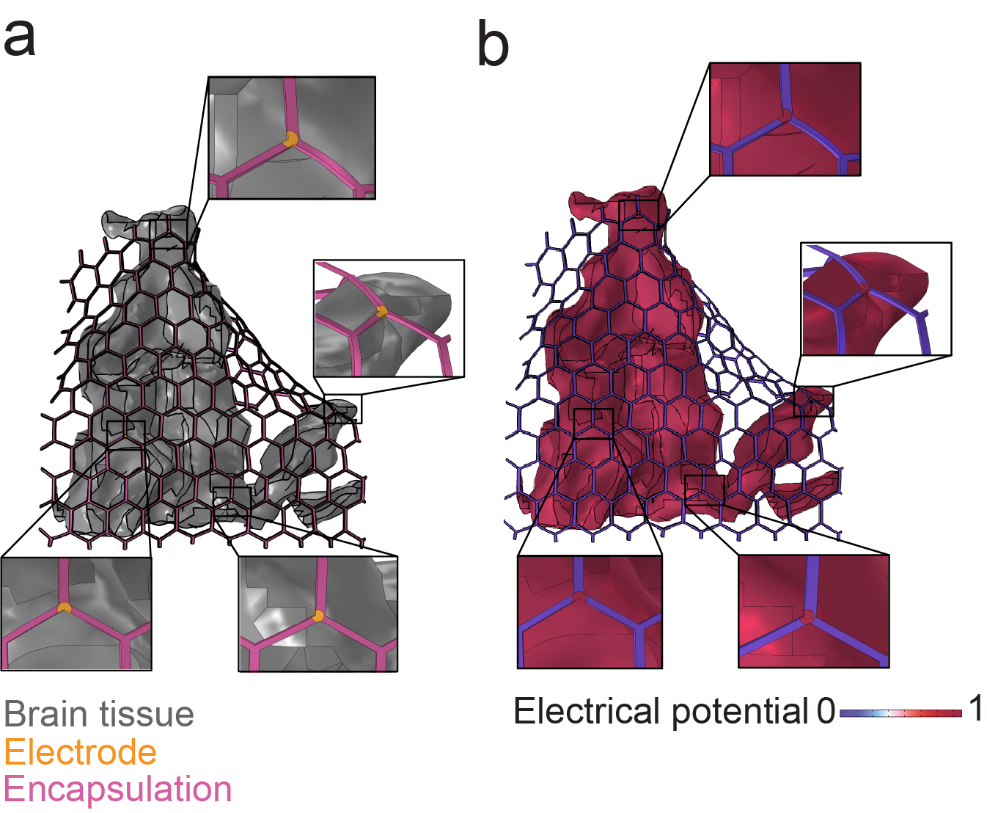
**

**Figure S3. Detailed schematic illustration of patient-specific design on HiPGE. a)** Totally four locations were selected for electrodes on HiPGE. **b)** All electrodes are connected to the electrochemical signal on the cortical surface of the brain tissue.

**
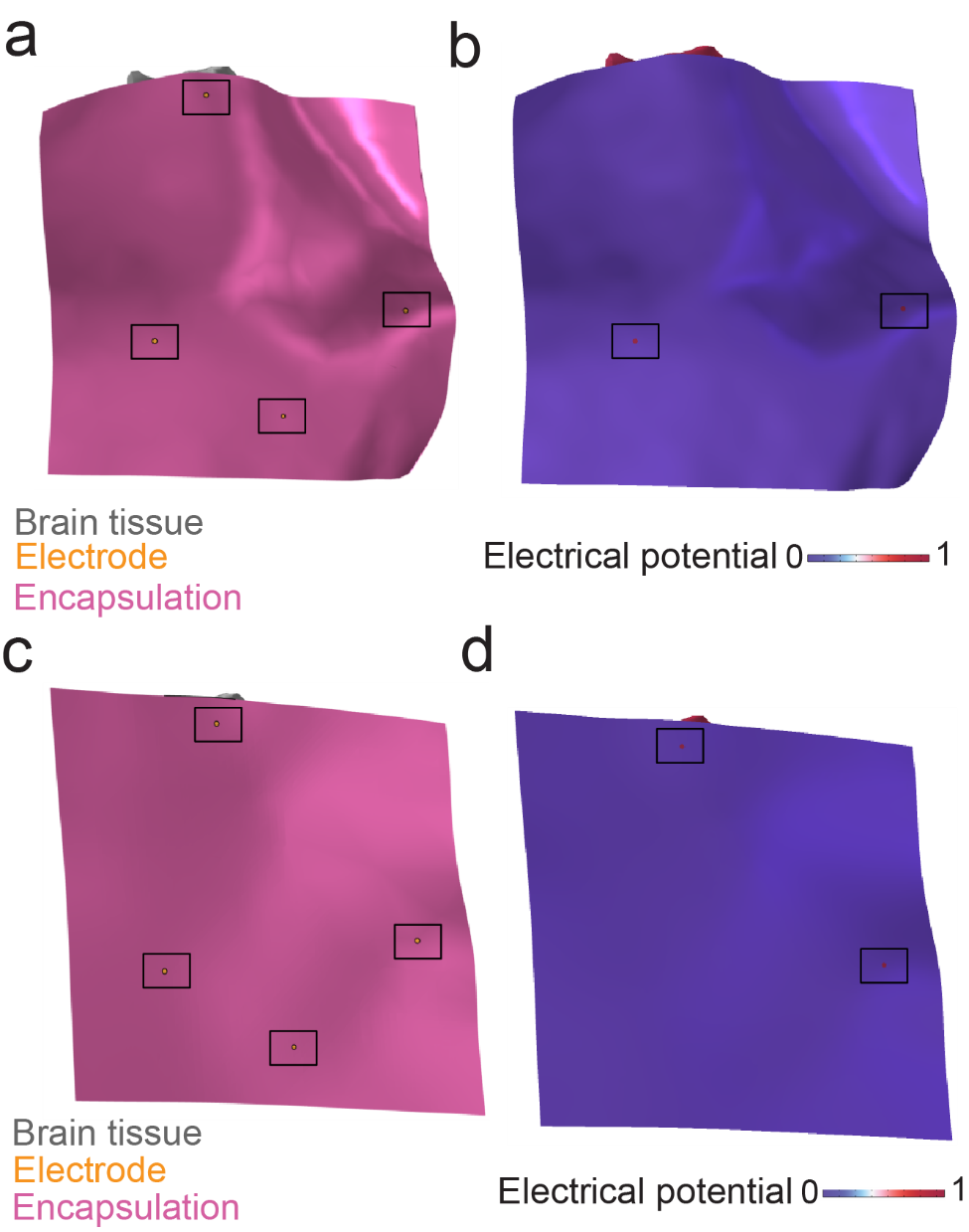
**

**Figure S4. In silico modeling of connectivity test on PDMS (a-b) and SBS (c-d) control devices**. For PDMS-brain assembly (a) and SBS-brain assembly (c), four of the same locations were selected for electrodes as HiPGE. Only two out of four electrodes successfully connected to the electrical signal, both on PDMS (b) and SBS (d) control devices.

**
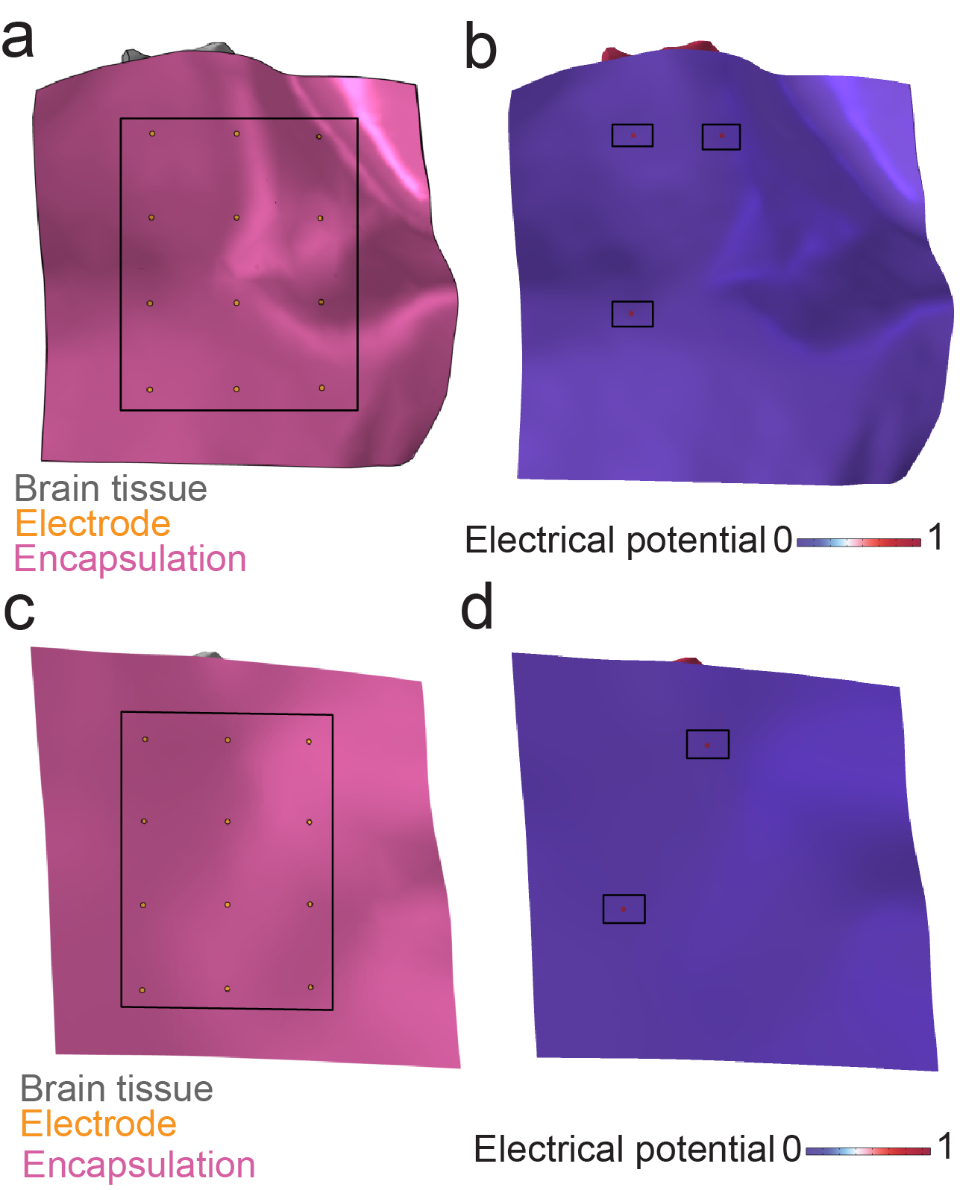
**

**Figure S5. In silico modeling of connectivity test on PDMS (a-b) and SBS (c-d) matrix devices.** Conventional matrix-aligned electrodes were used (a and c). Only a few electrodes are connected to the electrical signal (b and d).

**
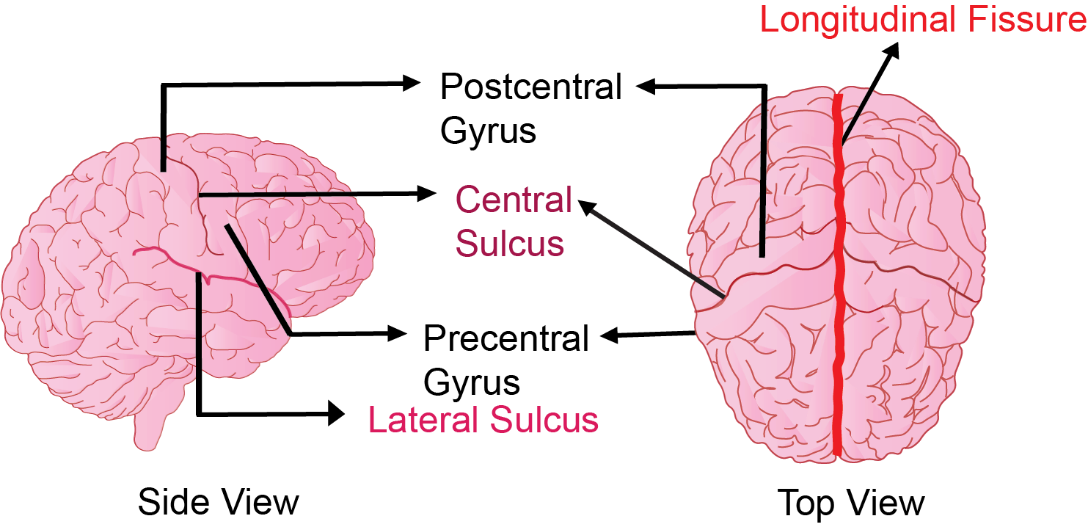
**

**Figure S6. Schematic of brain segmentation as anatomical reference points for the SM region.**


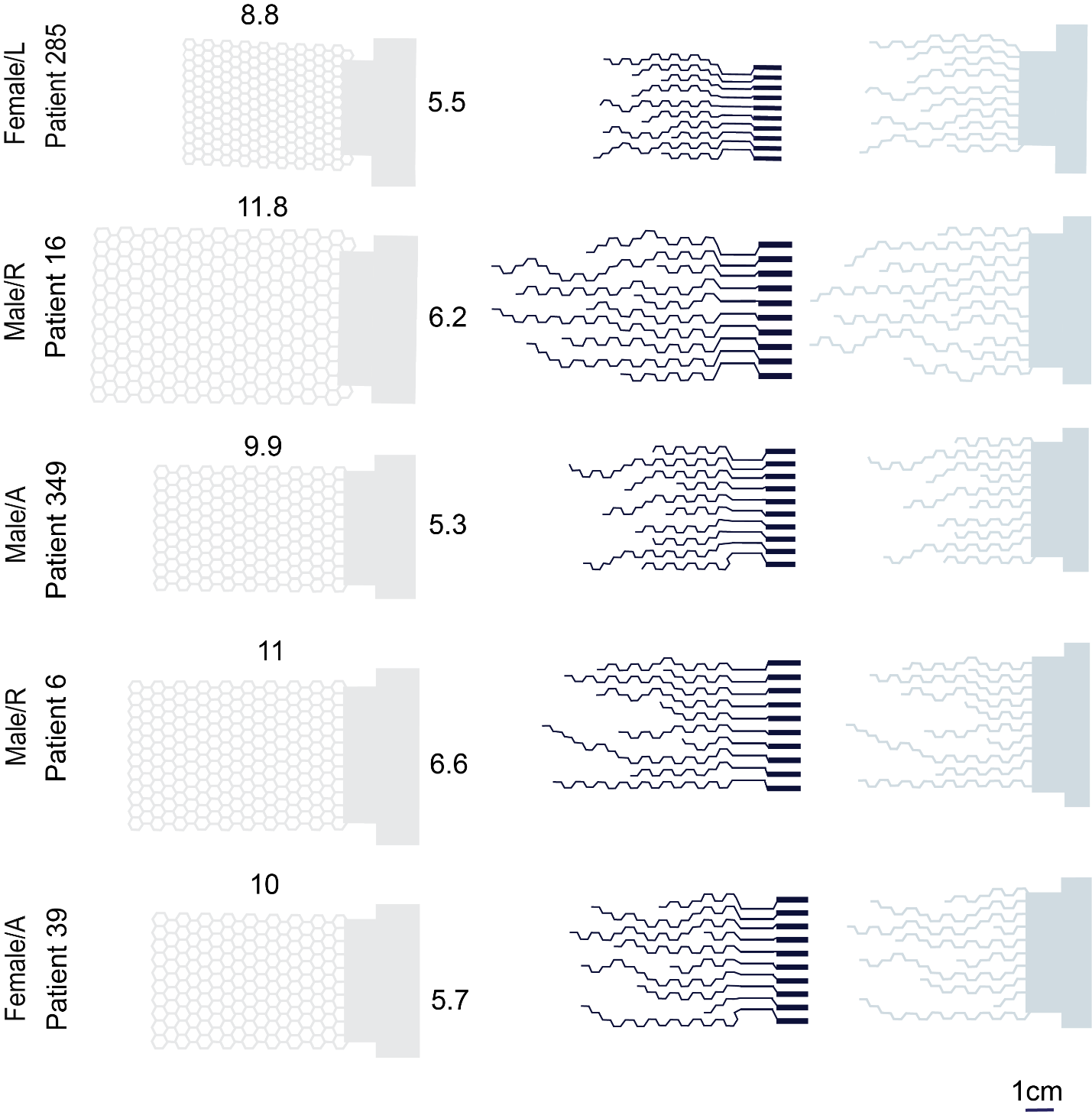


**Figure S7. Schematics of personalized designs of HiPGE based on patients’ SM regions.** (Handedness; A = ambidextrous, L = left-handed, R = right-handed).

**
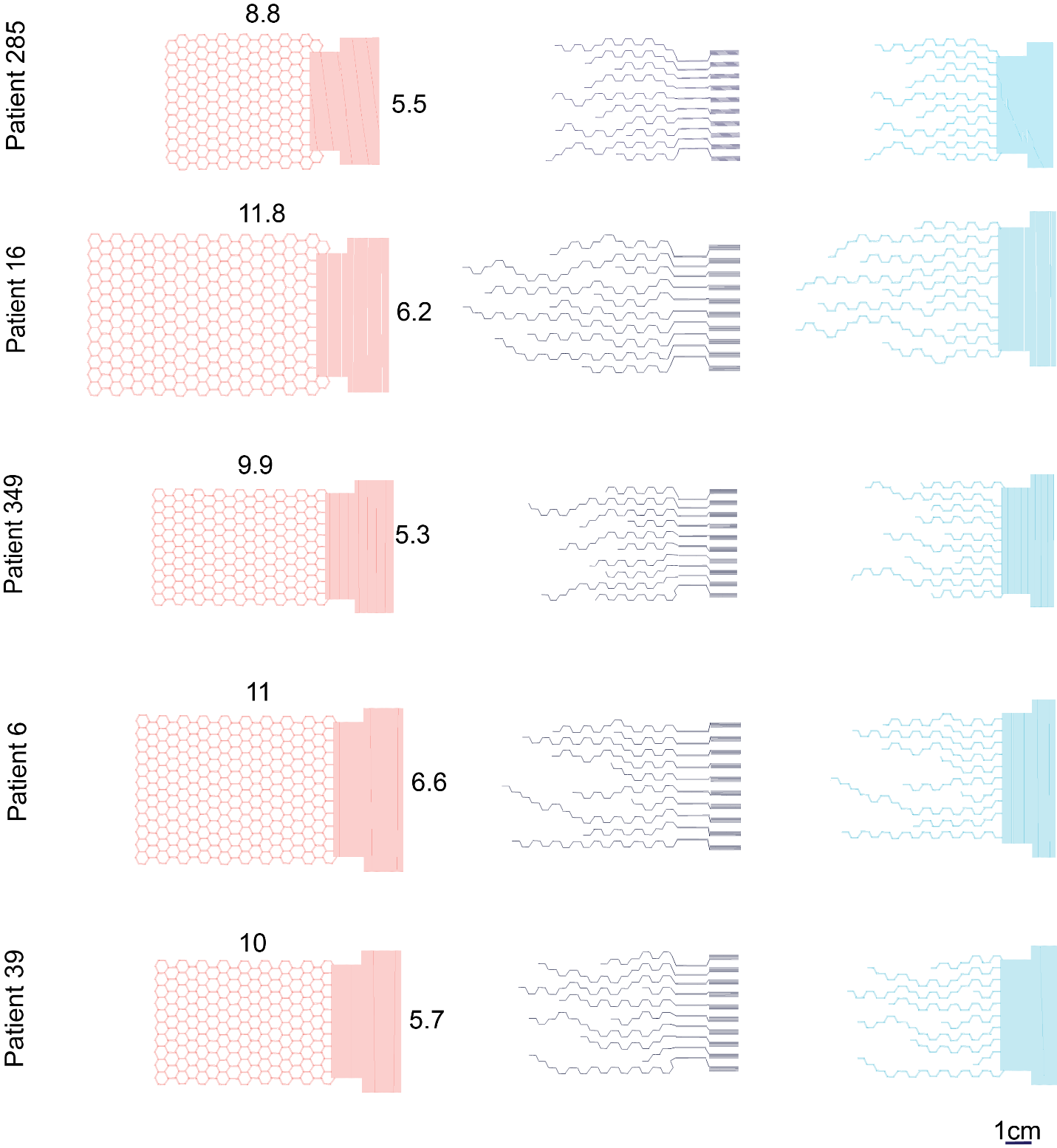
**

**Figure S8. Printing pathways of HiPGE for interfacing with ECoG recording.**

**
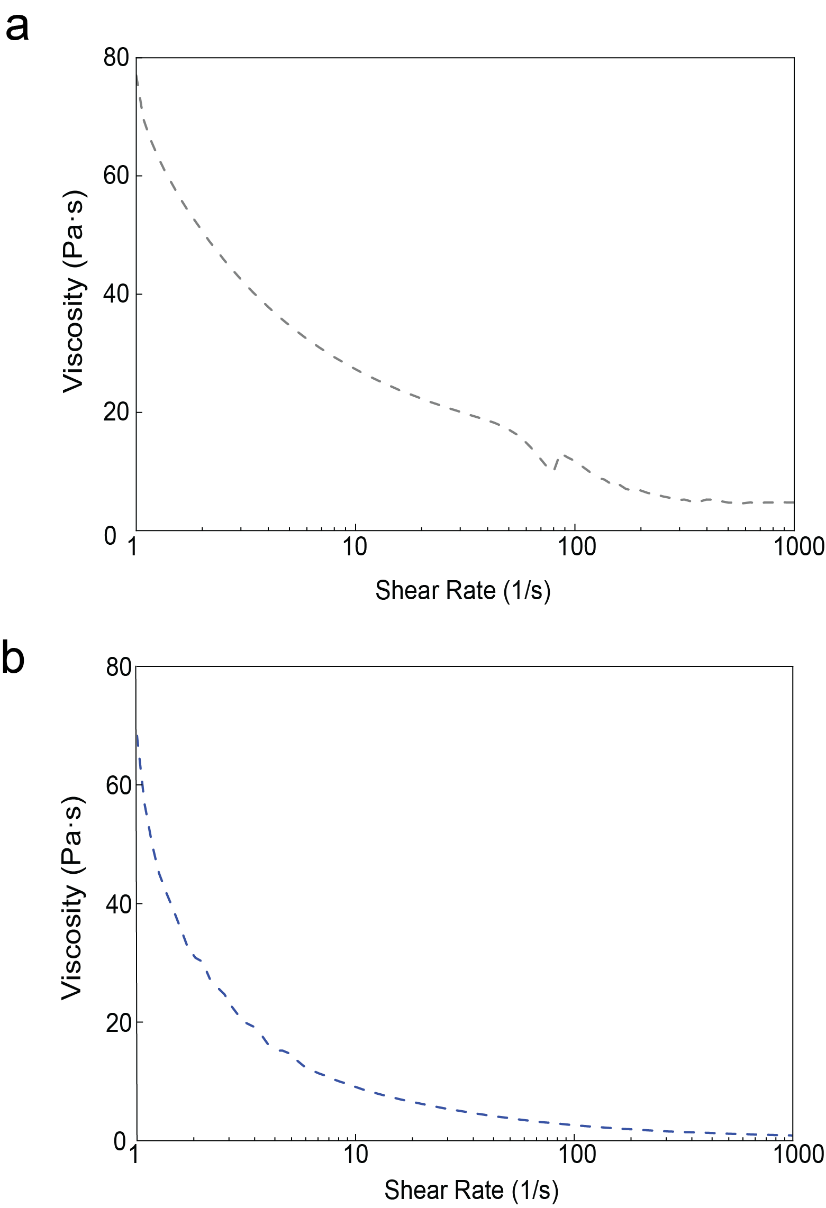
**

**Figure S9. Rheological properties of HiPGE’s encapsulation ink (a) and conductive ink (b).**

**
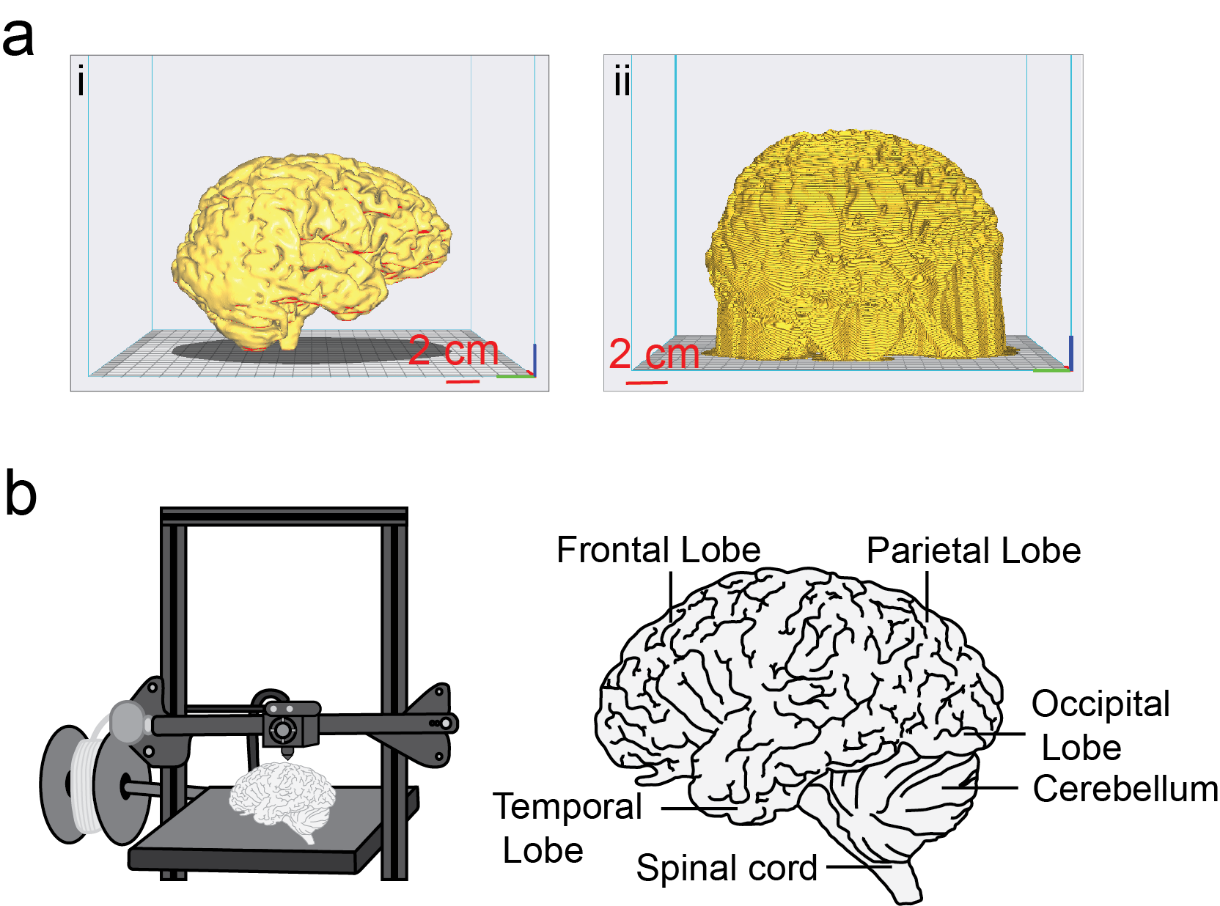
**

**Figure S10. 3D printing of patient brain models. a)** 3D model of the brain without (i) and with (ii) support layer, for 3D printing as a demonstration (a). **b)** Schematic of the 3D printed human brain by FDM 3D printers (b).

**
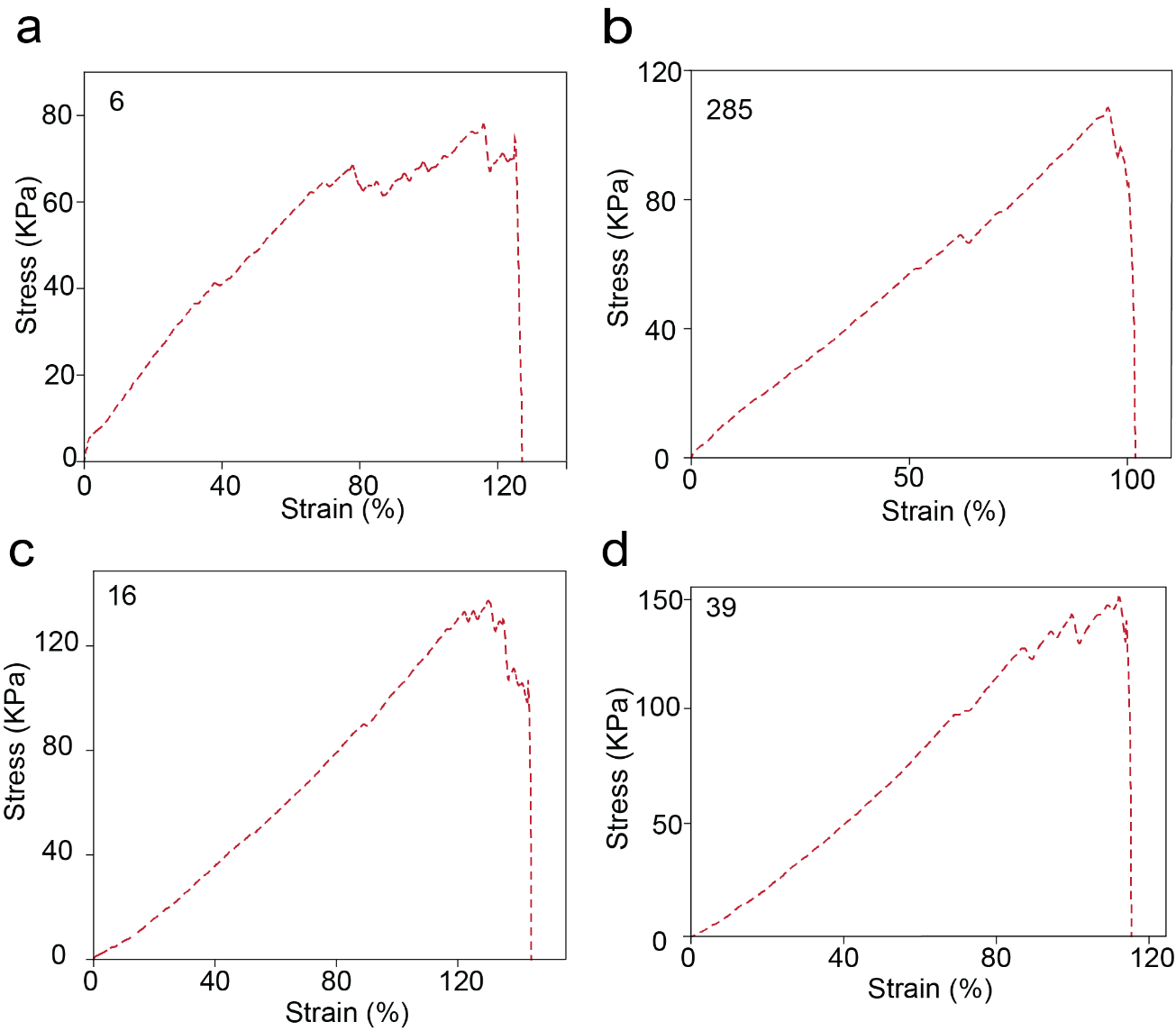
**

**Figure S11.** Engineering stress vs engineering strain curve for patient-specific HiPGEs.

**
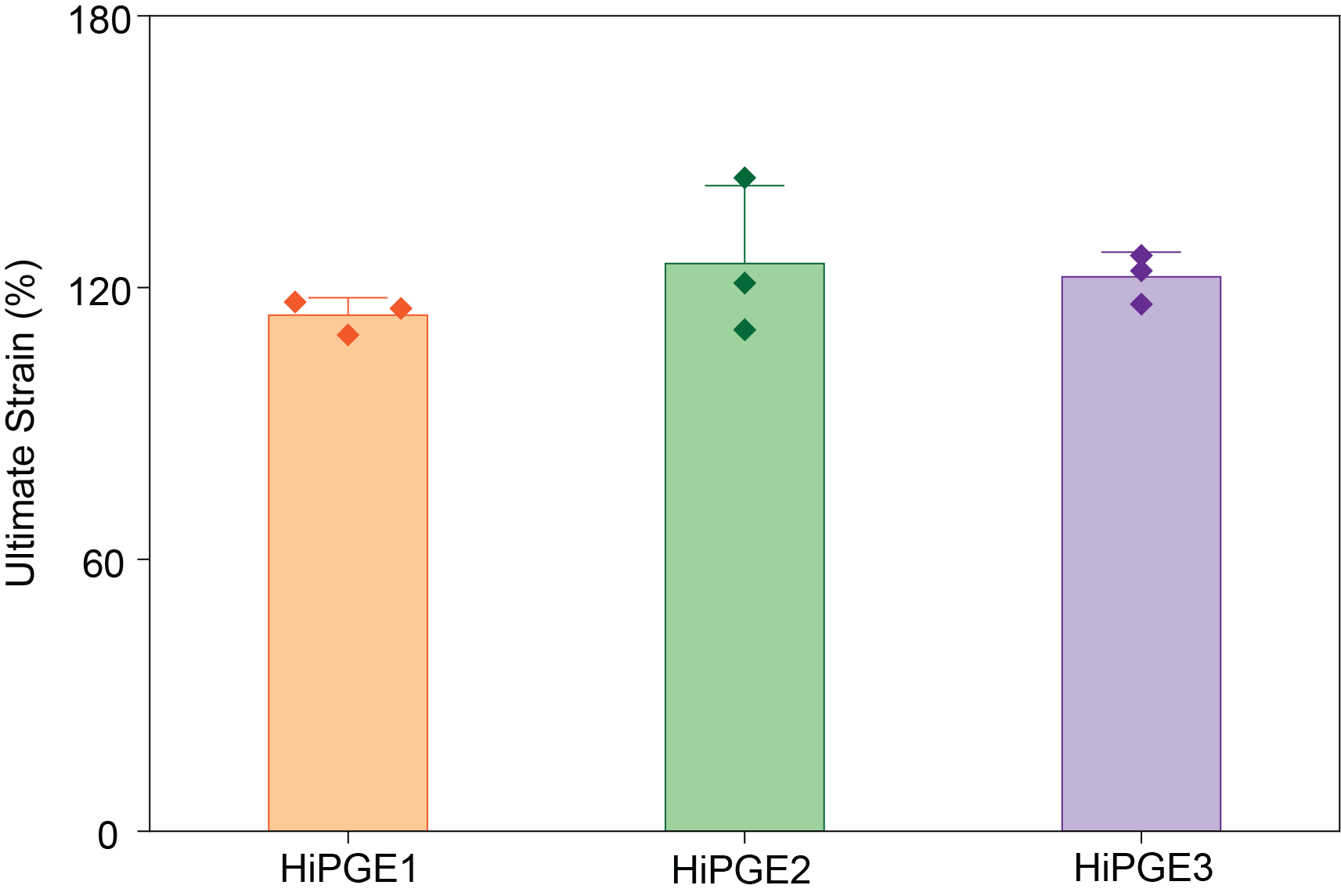
**

**Figure S12. Statistical analysis of ultimate strain for HiPGEs.** Values represent mean and standard deviation (SD) (*n*=3; independent samples).

**
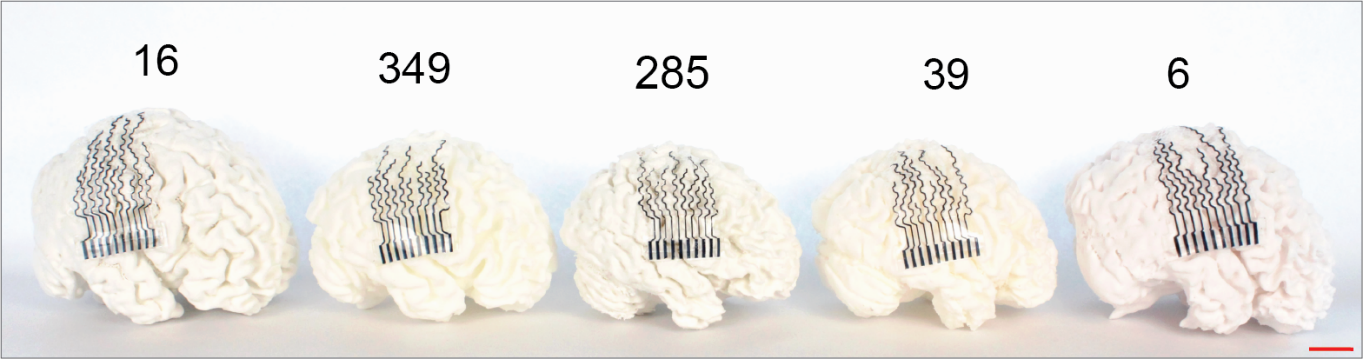
**

**Figure S13.** Demonstrating different sizes of personalized HiPGEs on their corresponding patients’ 3D-printed brain models. Scale bar, 3 cm.

**
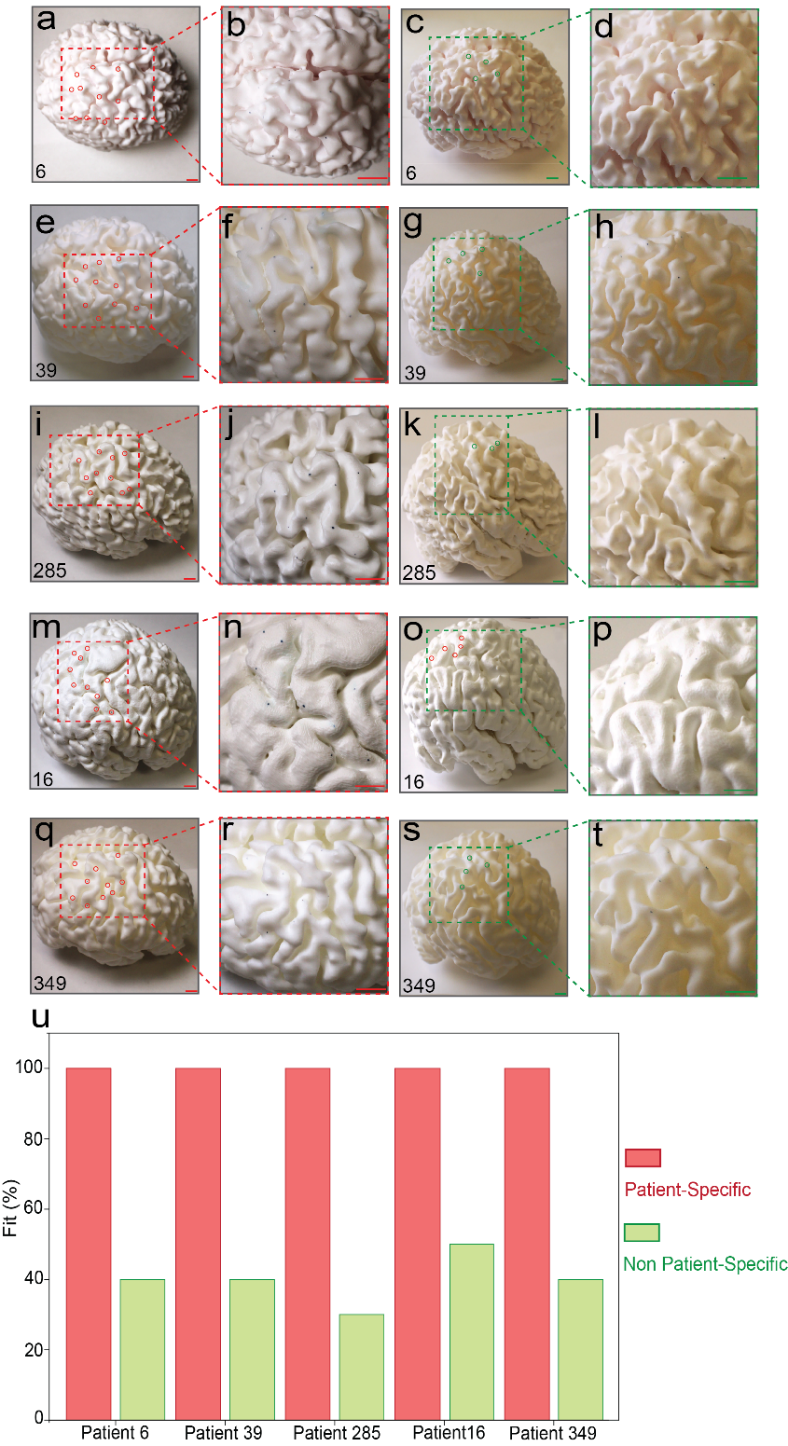
**

**Figure S14.** **Quantitative conformity analysis of patient-specific vs. non-patient-specific designs.** Representative conformity results for five different patients are shown after dye-labeling the electrodes for contrast enhancement. **a-d)** Patient 6: patient-specific design (a) with zoomed view of a selected cortical region (b), compared with non-patient-specific design (c) and its zoomed view (d). **e-h)** Patient 39: patient-specific (e,f) vs. non-patient-specific (g,h). **i-l)** Patient 285: patient-specific (i,j) vs. non-patient-specific (k,l). **m-p)** Patient 16: patient-specific (m,n) vs. non-patient-specific (o,p). **q-t)** Patient 349: patient-specific (q,r) vs. non-patient-specific (s,t). In each comparison, patient-specific designs exhibit superior conformity to sulcal and gyral geometries. Scale bars: 1 cm. **u)** Quantitative percentage of cortical surface coverage for patient-specific vs. non-patient-specific designs across all five patients.

**
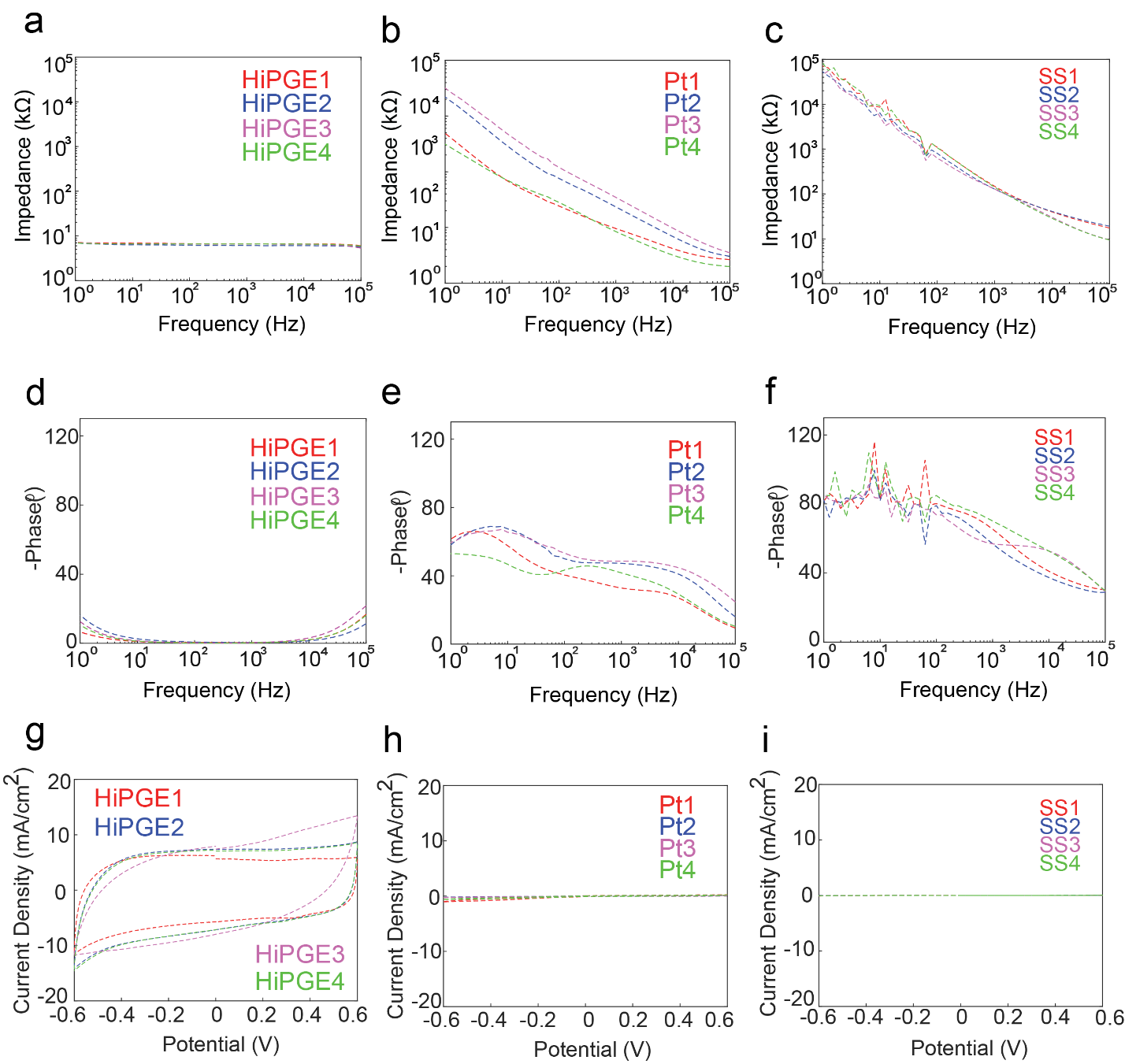
**

**Figure S15. Comparison of impedance (a-c), phase (d-f), and current density (g-i) among HiPGE, Pt electrode, and SS electrode.**

**
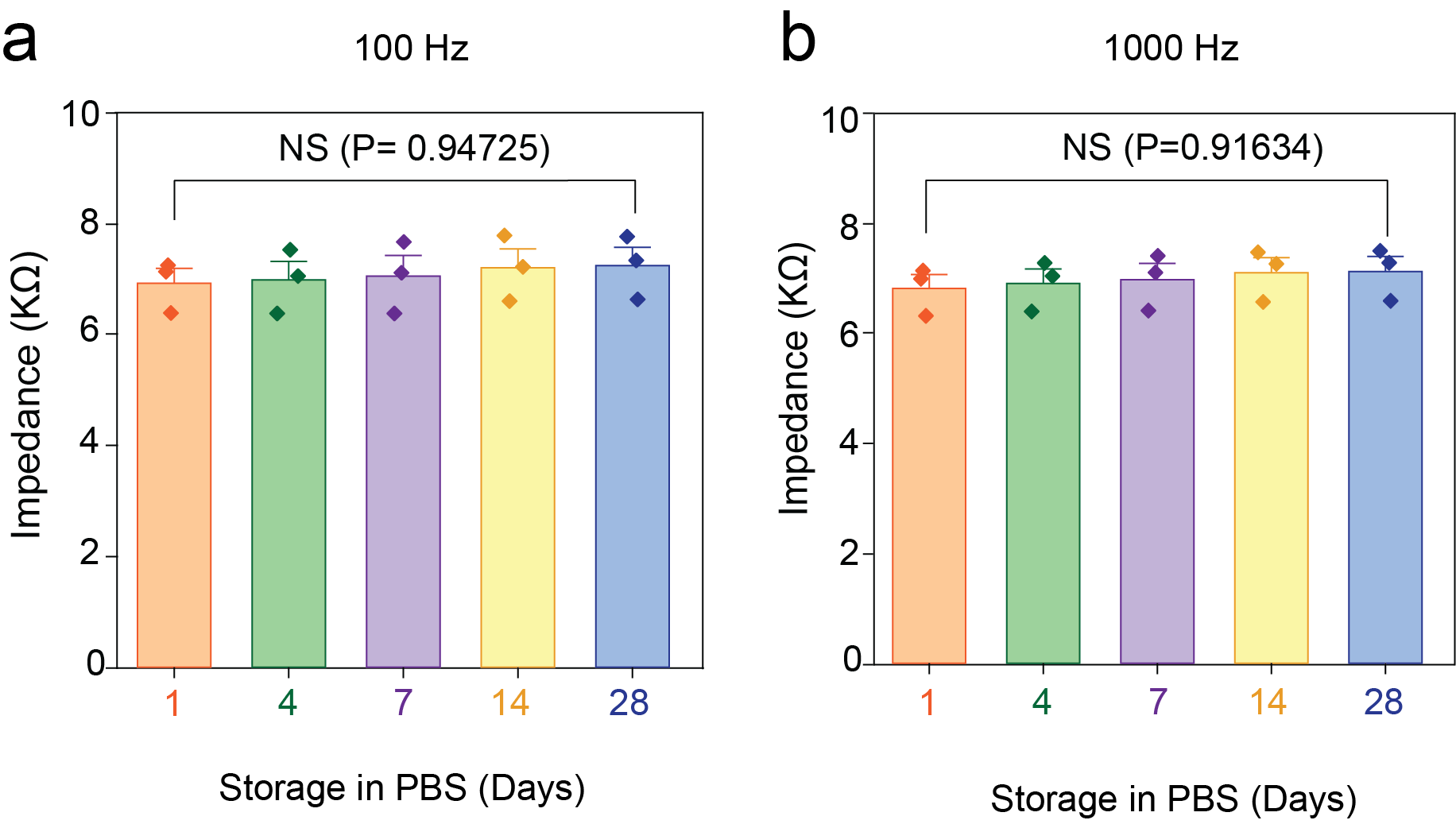
**

**Figure S16. Impedance of HiPGE on different days in a PBS bath at 50°C.** Measurement of impedance at 100 Hz (a) and at 1 K Hz (b). Values represent mean and standard errors of the mean (SEM) (*n*=3; independent samples). Statistical significance and P values are determined by a one-way ANOVA test; NS, not significant.

**
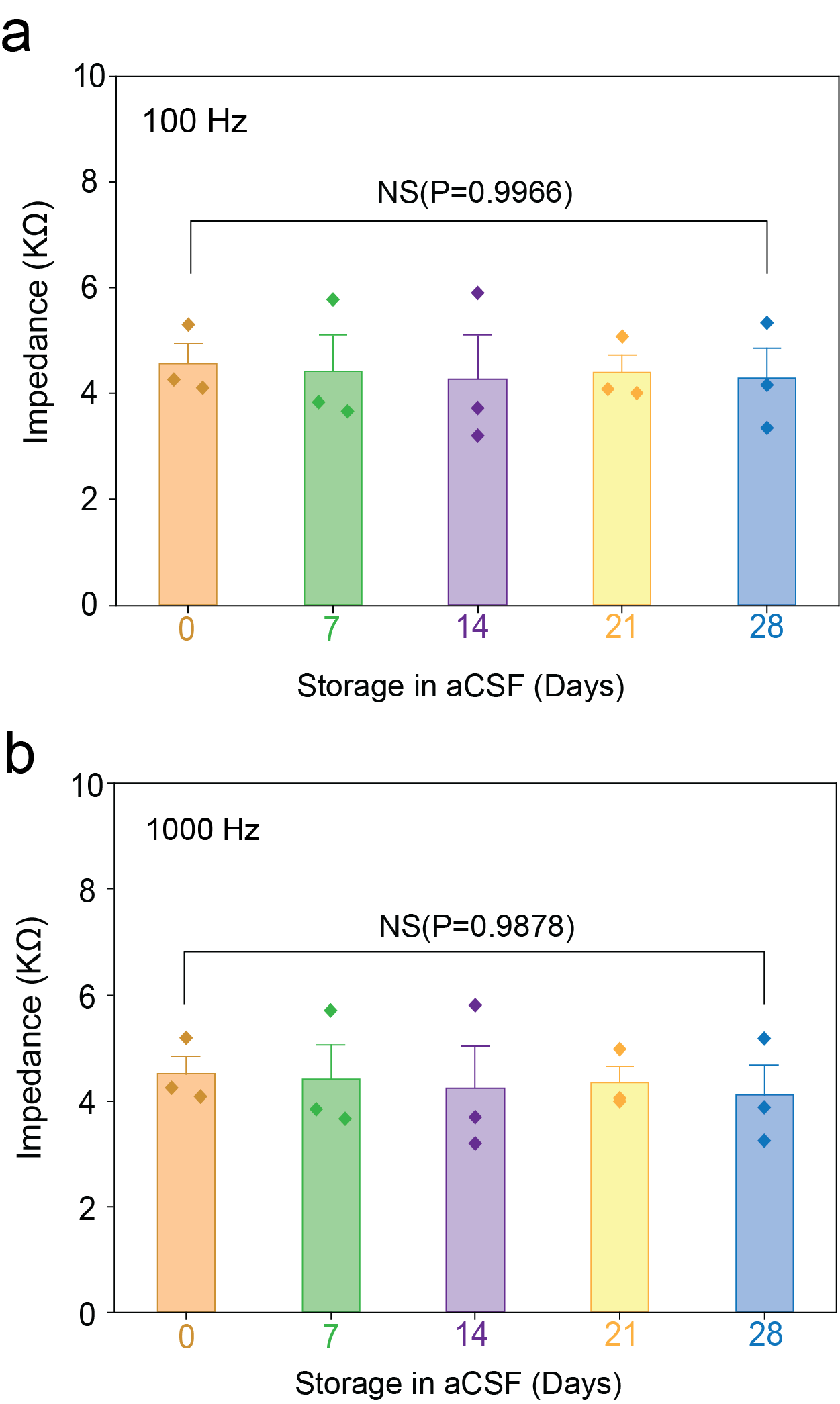
**

**Figure S17. Impedance of HiPGE on different days in aCSF bath at 37°C.** Measurement of impedance at 100 Hz (a) and at 1 K Hz (b). Values represent mean and standard errors of the mean (SEM) (n=3; independent samples). Statistical significance and P values are determined by a one-way ANOVA test; NS, not significant.

**
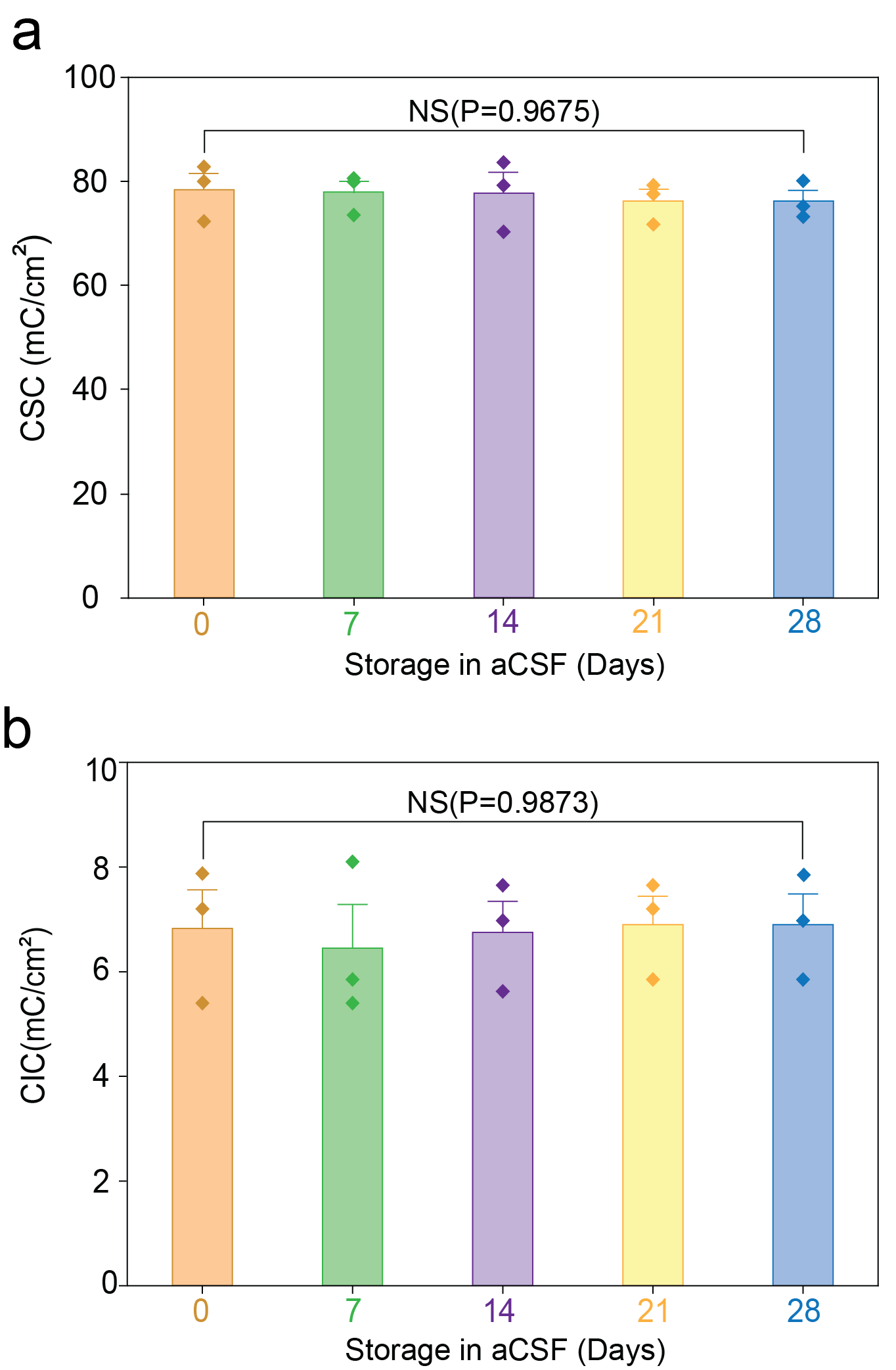
**

**Figure S18. Charge storage capacity (CSC) and charge injection capacity (CIC) of HiPGE on different days in aCSF bath at 37°C.** Measurement of CSC (a) and CIC (b). Values represent mean and standard errors of the mean (SEM) (n=3; independent samples). Statistical significance and P values are determined by a one-way ANOVA test; NS, not significant.

**
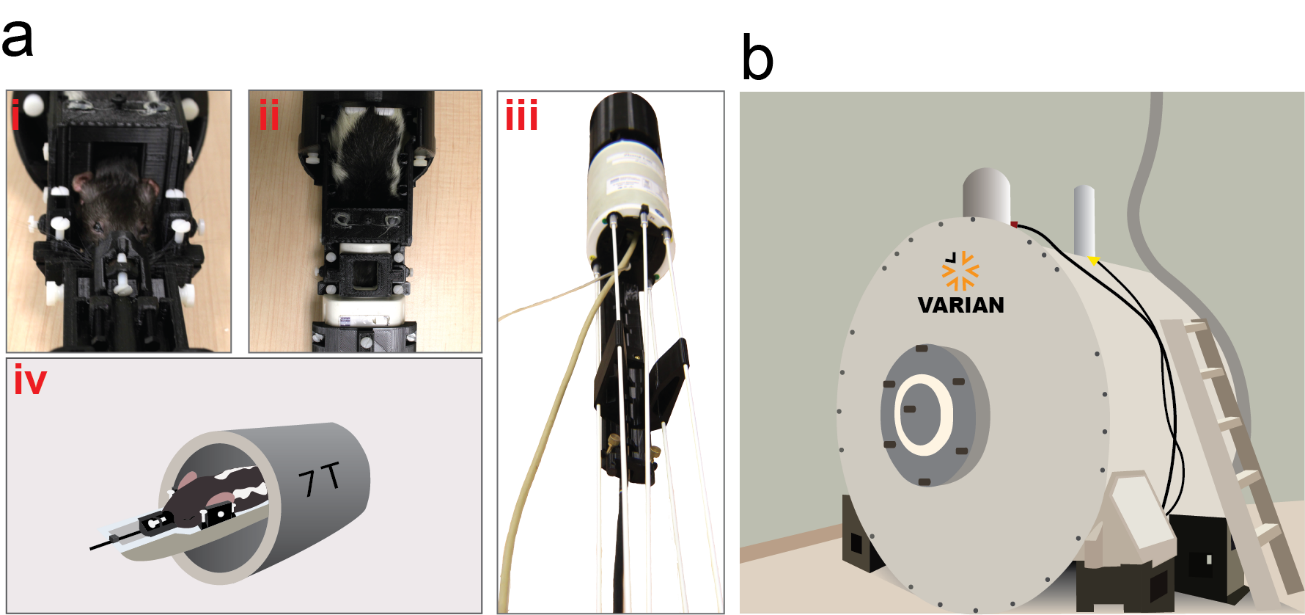
**

**Figure S19. Customized 3D printed restrainers for rat’s MRI scanning. a)** The animal setup for MRI scanning. **i,** The rat head is secured using a bite bar and two side bars. **ii,** The rat’s body is restrained by the body holder and a shoulder pressing bar. **iii,** the 3-channel coil is placed on the top of the rat's head. **iv**, The rat is fixed in a volume coil and placed in a 7T scanner. **b)** 7T MRI scanner for small animals.

**
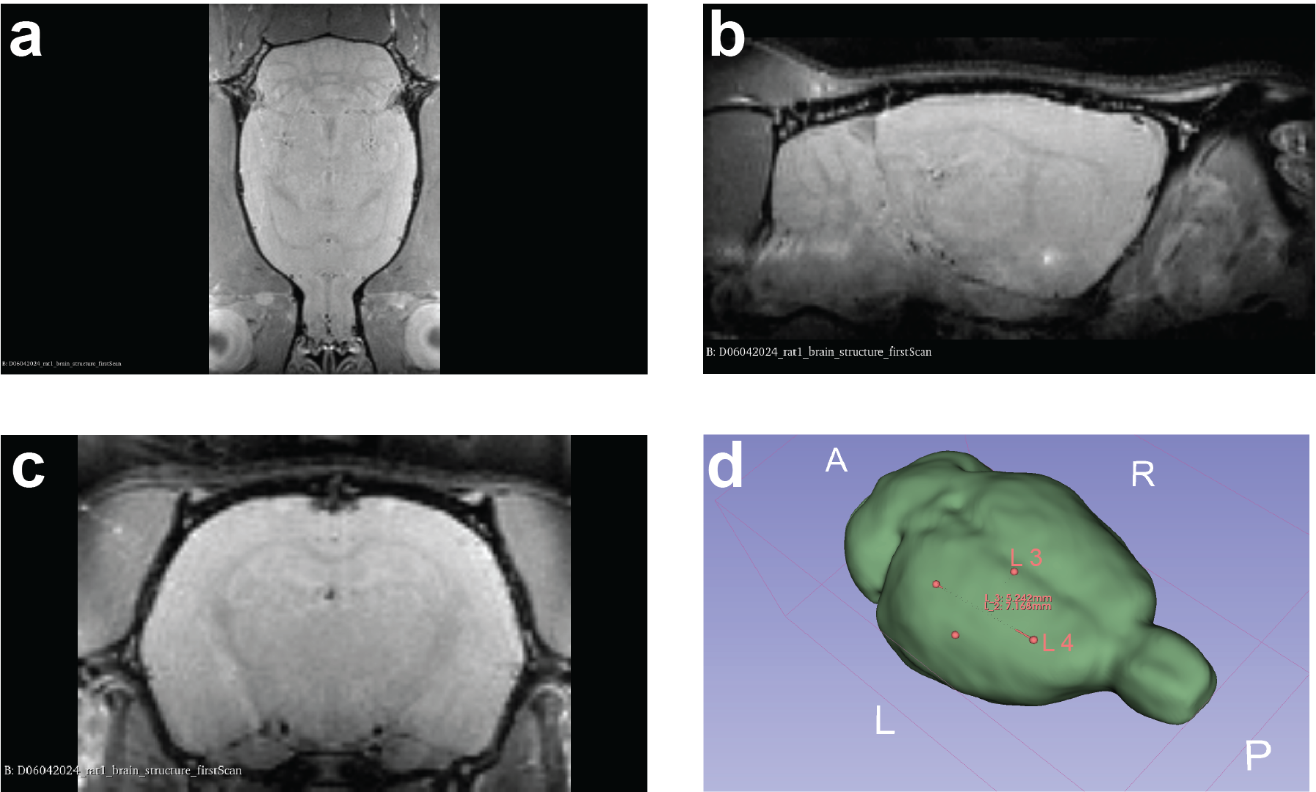
**

**Figure S20. Structural image of MRI scanning (a - Transverse, b - Sagittal, c -Frontal) and reconstruction of a 3D model (d) from structural images for a rat.**

**
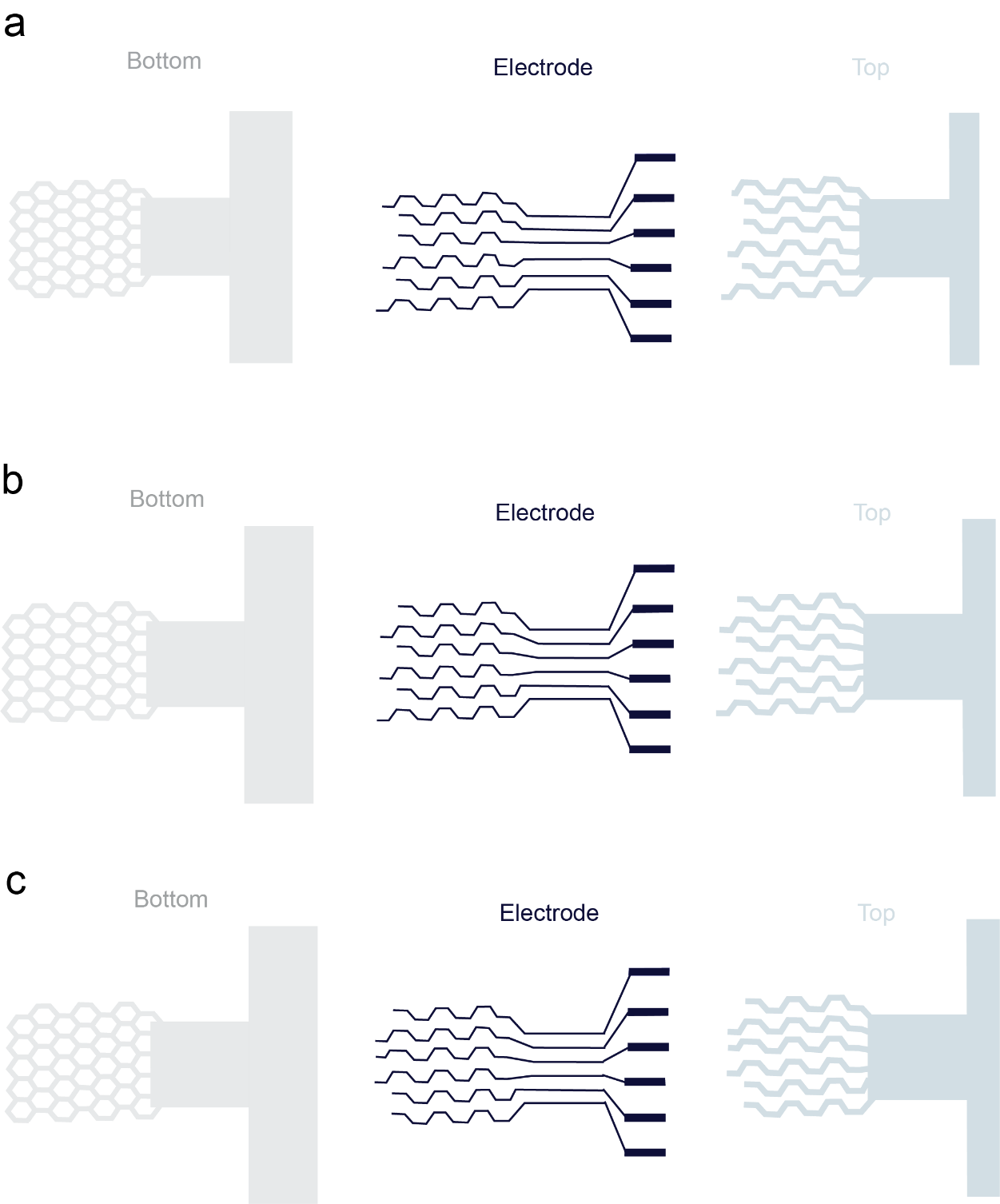
**

**Figure S21. Schematic of three layers of HiPGE for the three implanted rats for VEPs. Panels (a), (b), and (c) correspond to Rat#1, Rat#2, and Rat#3. Each schematic depicts the bottom, electrode, and top layers of the device.**

**
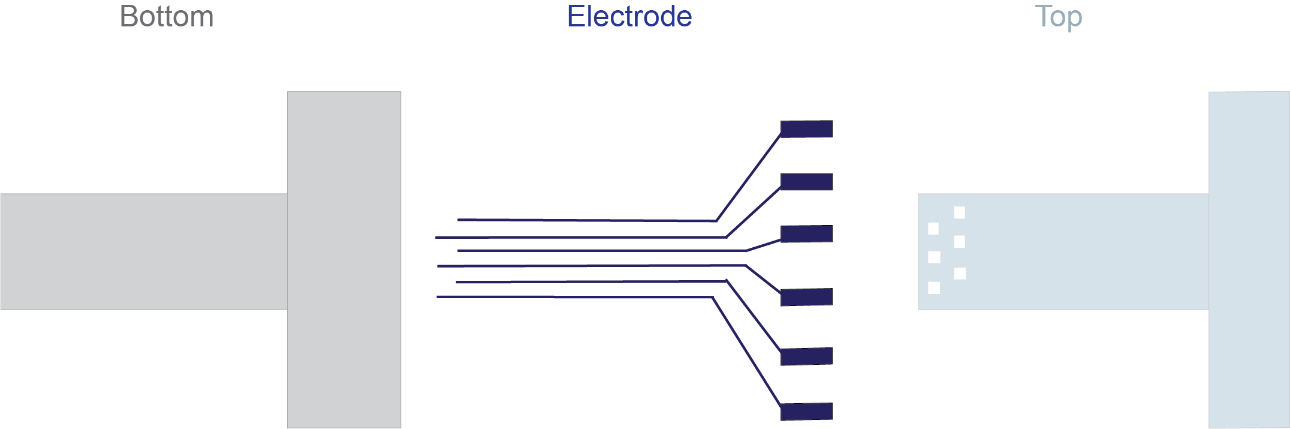
**

**Figure S22. Schematic of three layers of conventional electrodes.**

**
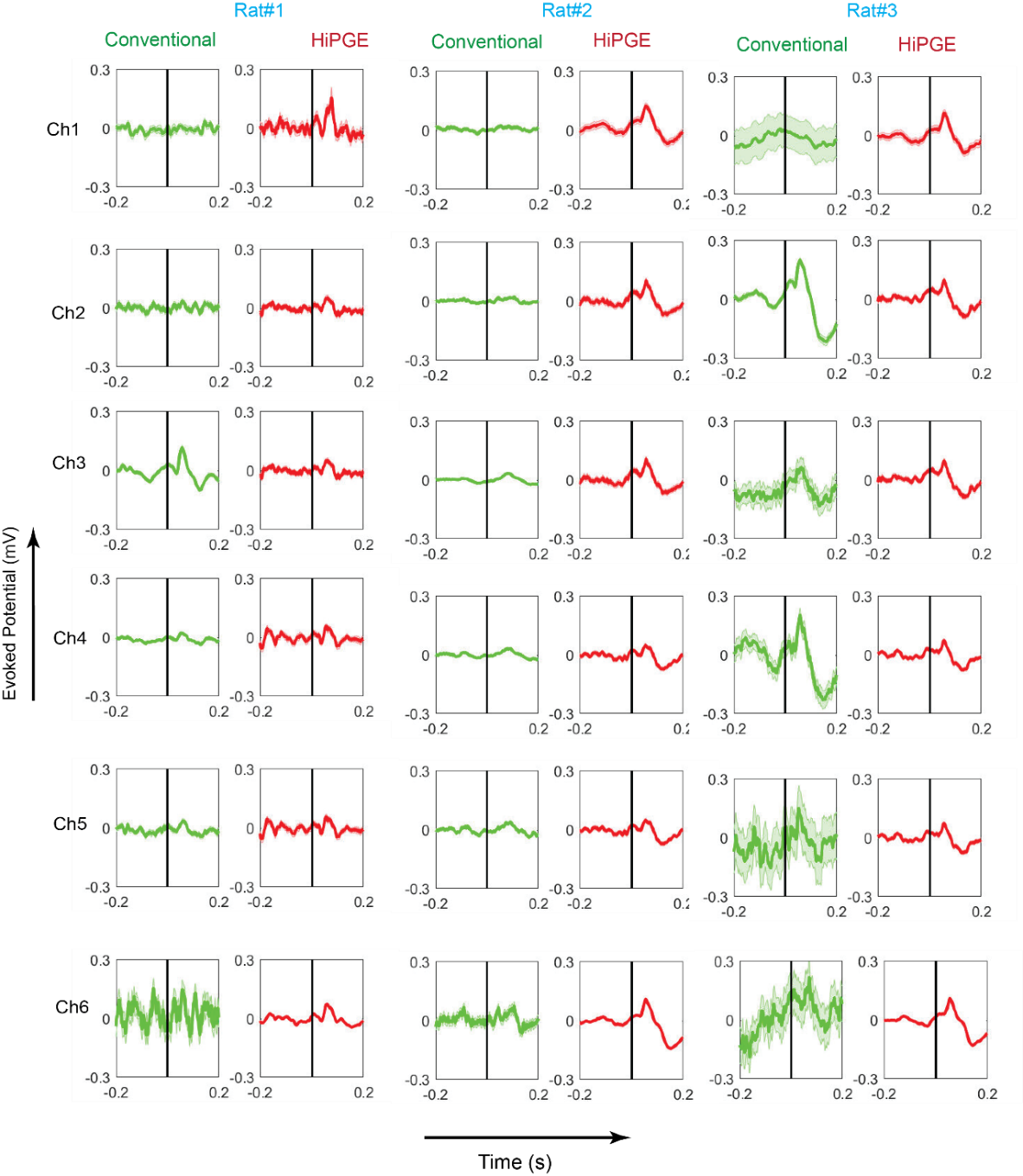
**

**Figure S23.** **Multichannel visual evoked potentials (VEPs) recorded in awake rats (n = 3).** Trial-averaged VEP waveforms from six channels across the visual cortex are shown for each rat, comparing recordings obtained using the conventional electrode (green) and the HiPGE (red). Traces are aligned to the onset of visual stimulation (vertical black line at 0 ms). Dark-colored lines represent the mean response across trials, and the light-colored shaded regions denote ±1 standard error, illustrating trial-to-trial variability. Results consistently show clear visually evoked responses across animals and channels for HiPGEs, while side channels of conventional electrodes demonstrate reduced signal quality, resulting in weaker or inconsistent evoked responses.


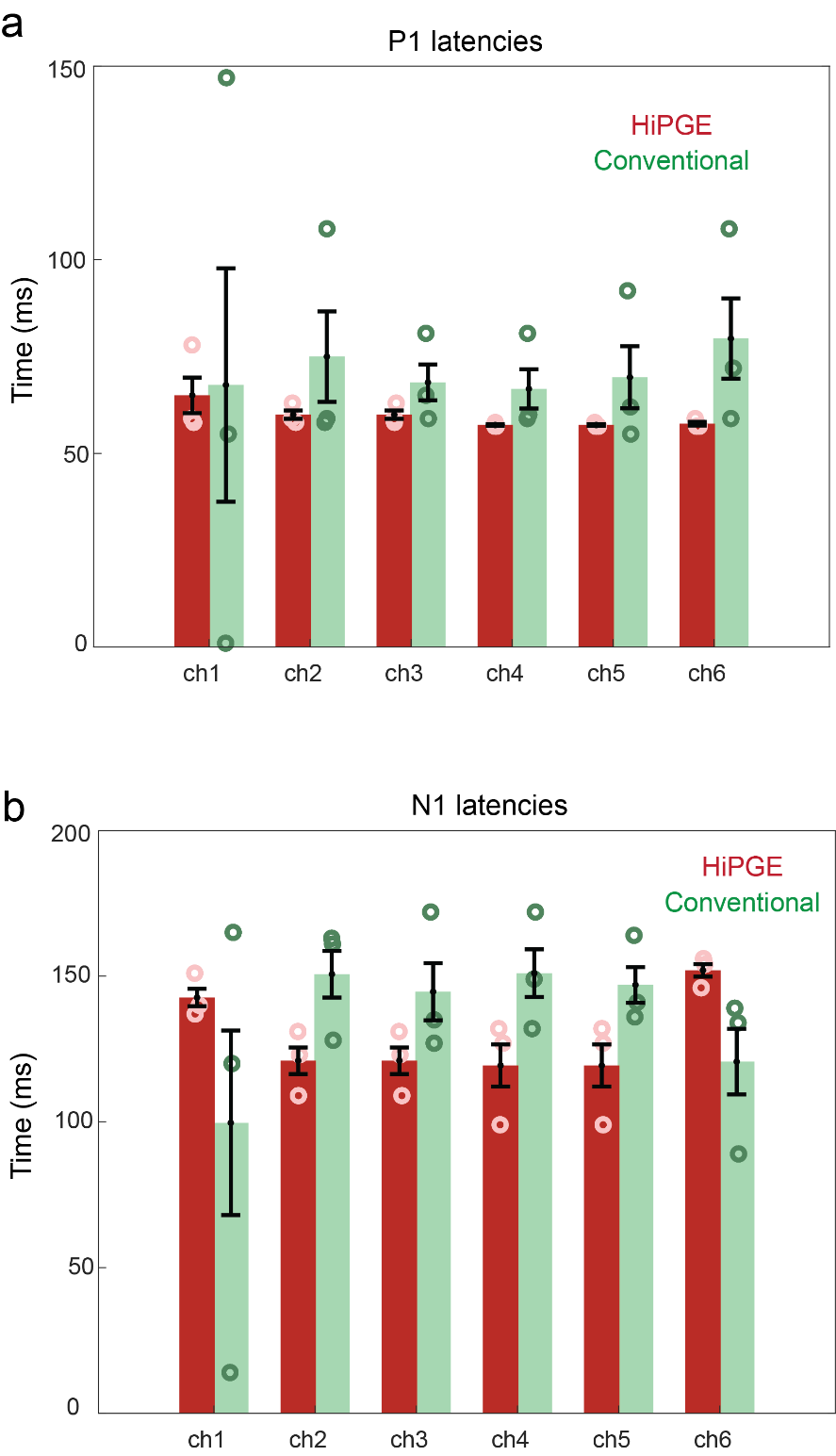


**Figure S24.** **Quantification of peak latencies from multichannel visual evoked potentials (VEPs).** Peak latencies were extracted from trial-averaged VEPs across six channels and compared between conventional electrodes (green) and HiPGEs (red). (a) Positive peak (P1) latencies and (b) negative peak (N1) latencies. Bar plots represent the mean latency across rats, with error bars indicating the standard error of the mean (n = 3 rats). Individual data points are shown as open circles, with each circle corresponding to peak latency for each rat. Comparisons between two groups were conducted using two-sample t-tests, with significance defined as *P < 0.05.

**
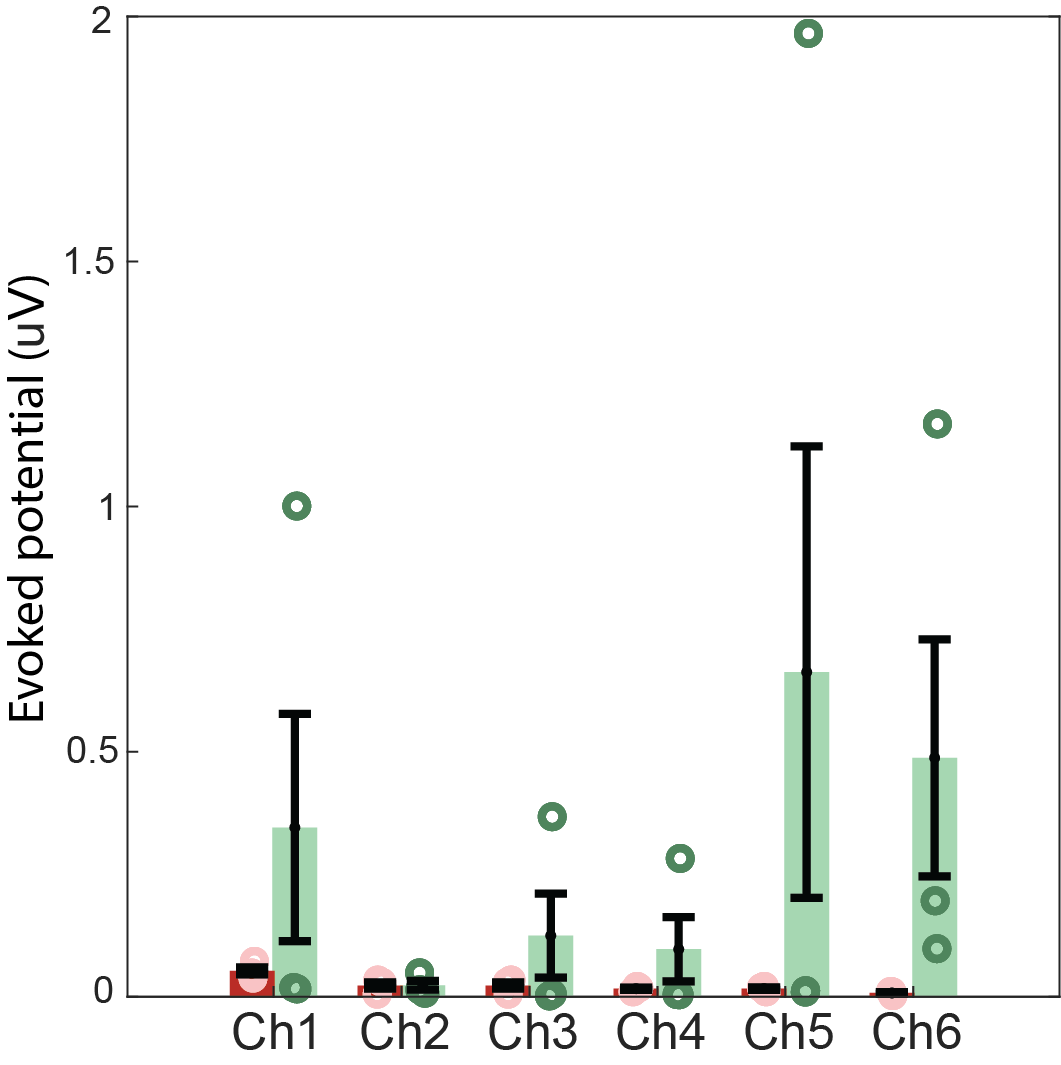
**

**Figure S25. Amplitude variance of visual evoked potentials (VEPs) across six channels.** Amplitude variance was computed from all trials for each rat, and individual data points (circles) represent the variance measured for each animal at each channel. Bars denote the mean amplitude variance across rats (n = 3), with error bars indicating the standard error of the mean (SEM).

**
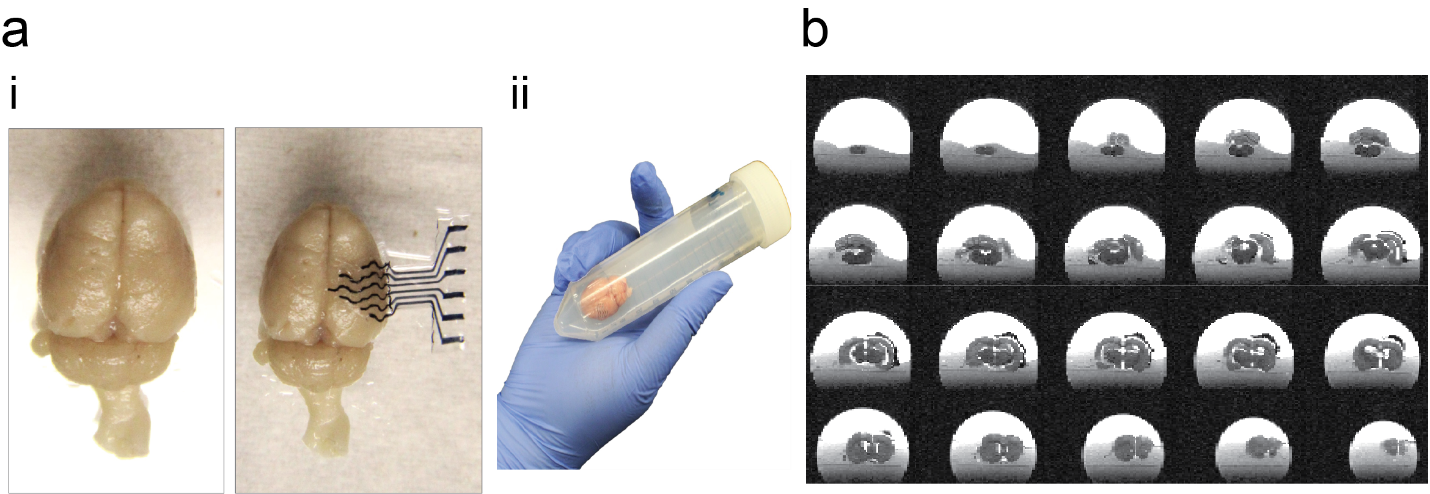
**

**Figure S26. Phantom test of HiPGE. a)** HiPGE was placed on the right hemisphere of a rat brain (i). 2% w/v agarose in a tube and HiPGE attached to the brain was immersed in that tube, then the tube was filled with saline (ii). **b)** Phantom test of HiPGE. T2*-weighted images of the brain with an electrode showing no significant artifact.


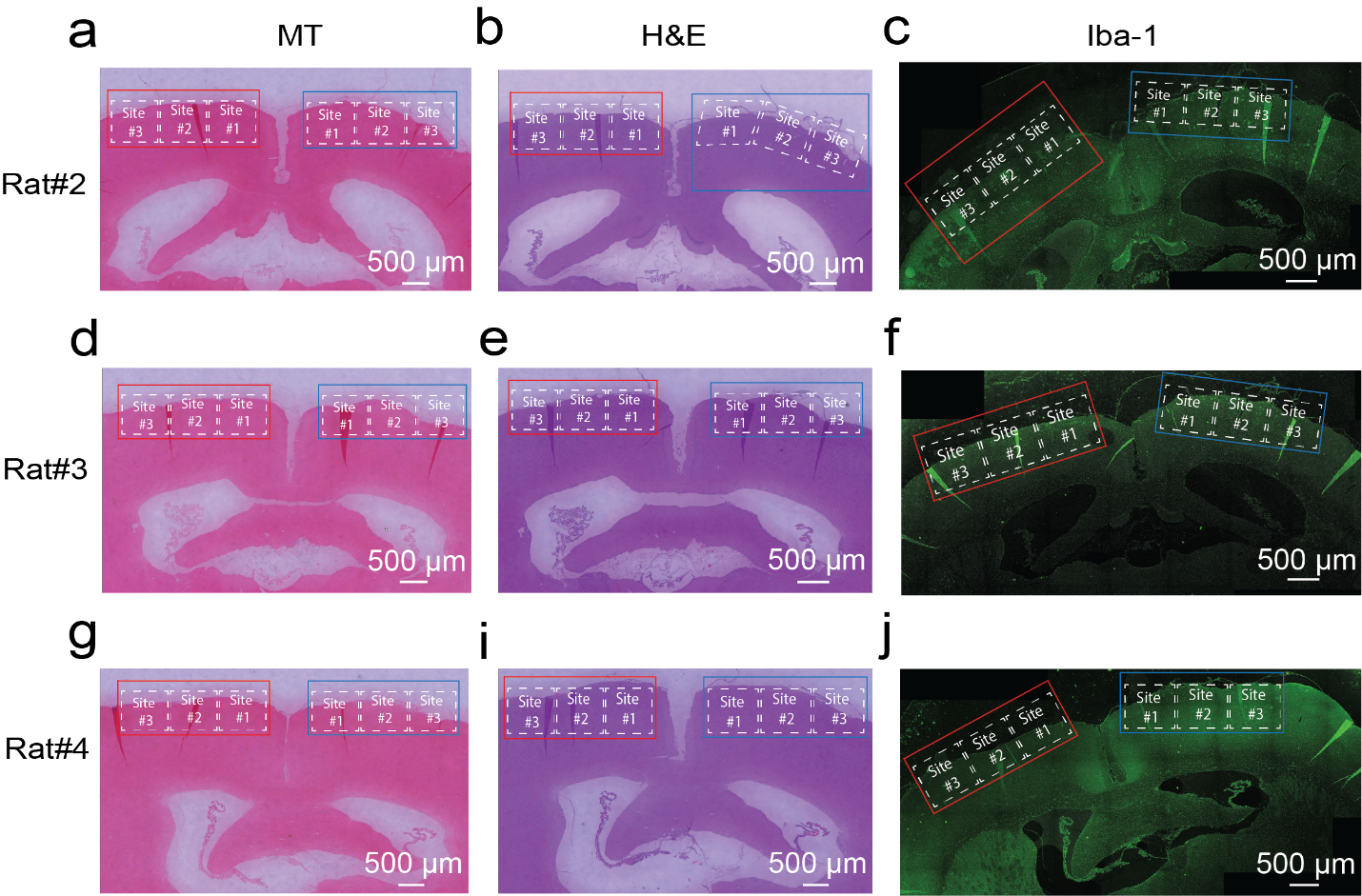


**Figure S27. Illustrations of histological images of the rat’s cortical surface after 4 weeks of implantation (n = 3 rats).** a-c) Masson’s Trichrome, Hematoxylin and Eosin, Iba-1 staining for Rat#2. d-f) Masson’s Trichrome, Hematoxylin and Eosin, iba-1 staining for Rat#3. g-j) Masson’s Trichrome, Hematoxylin and Eosin, Iba-1 staining for Rat#4. Red and blue squares refer to non-implant and implant regions, respectively.


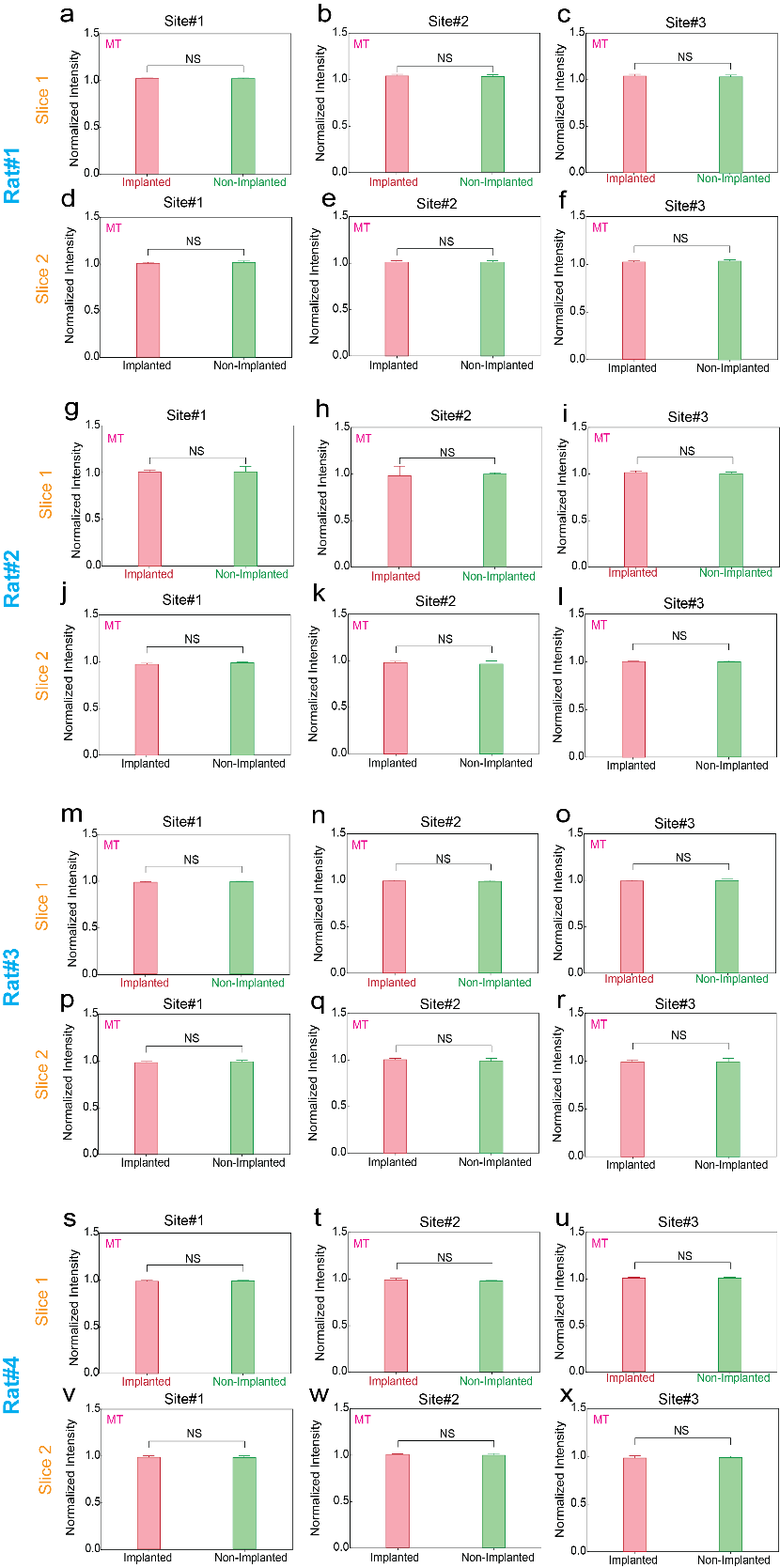


**Figure S28. Quantitative analysis of normalized Masson’s Trichrome staining intensity near the probe boundary across multiple slices and implantation sites for all rats.** a–f correspond to Rat #1, g–l to Rat #2, m–r to Rat #3, and s–x to Rat #4. Bar plots show the mean ± SD intensity for implanted and non-implanted regions, where ‘NS’ denotes no statistically significant difference (P > 0.05, two-sample t-test).


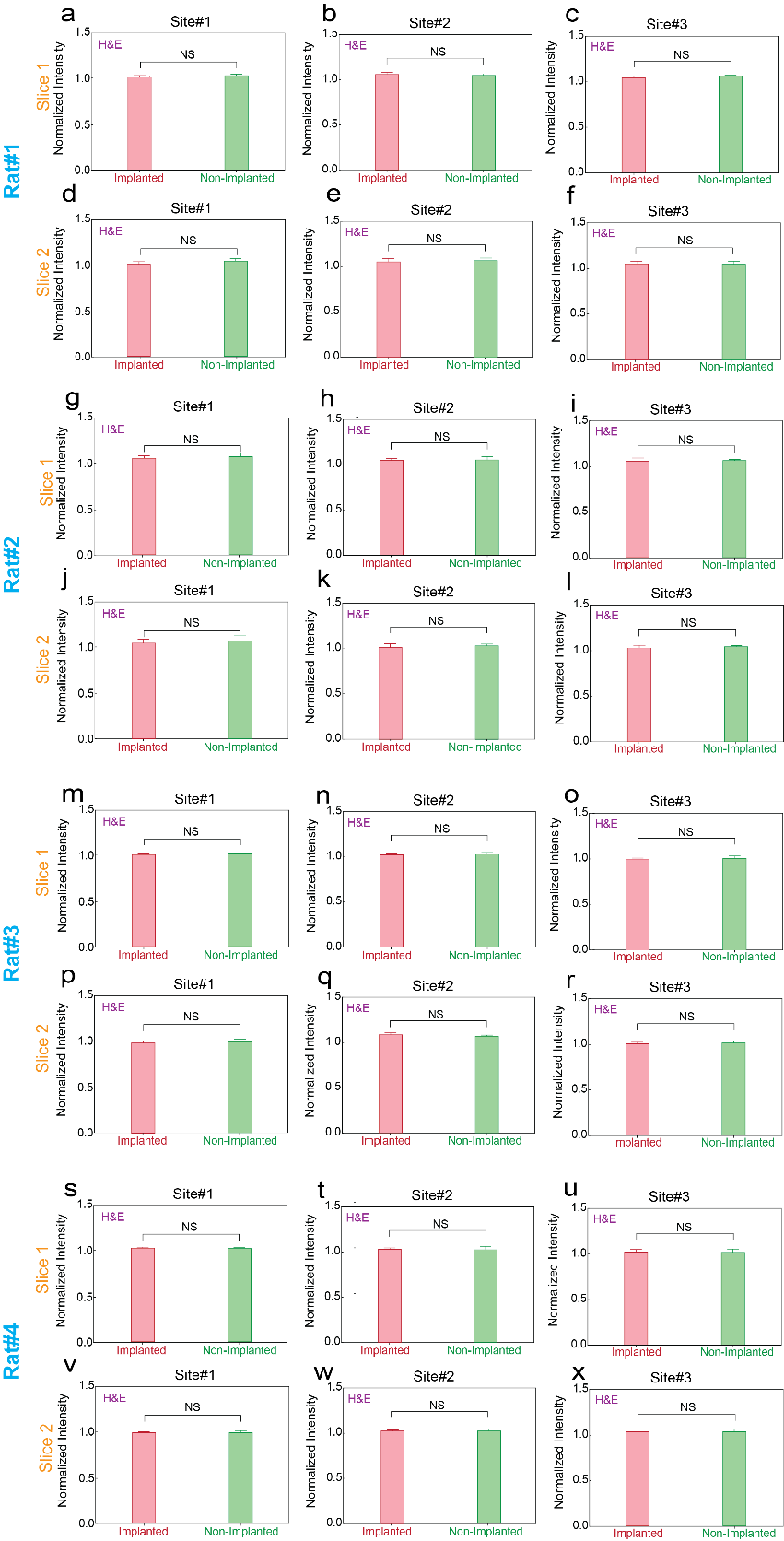


**Figure S29. Quantitative analysis of normalized H&E staining intensity near the probe boundary across multiple slices and implantation sites for all rats.** a–f correspond to Rat #1, g–l to Rat #2, m–r to Rat #3, and s–x to Rat #4. Bar plots show the mean ± SD intensity for implanted and non-implanted regions, where ‘NS’ denotes no statistically significant difference (P > 0.05, two-sample t-test).

**
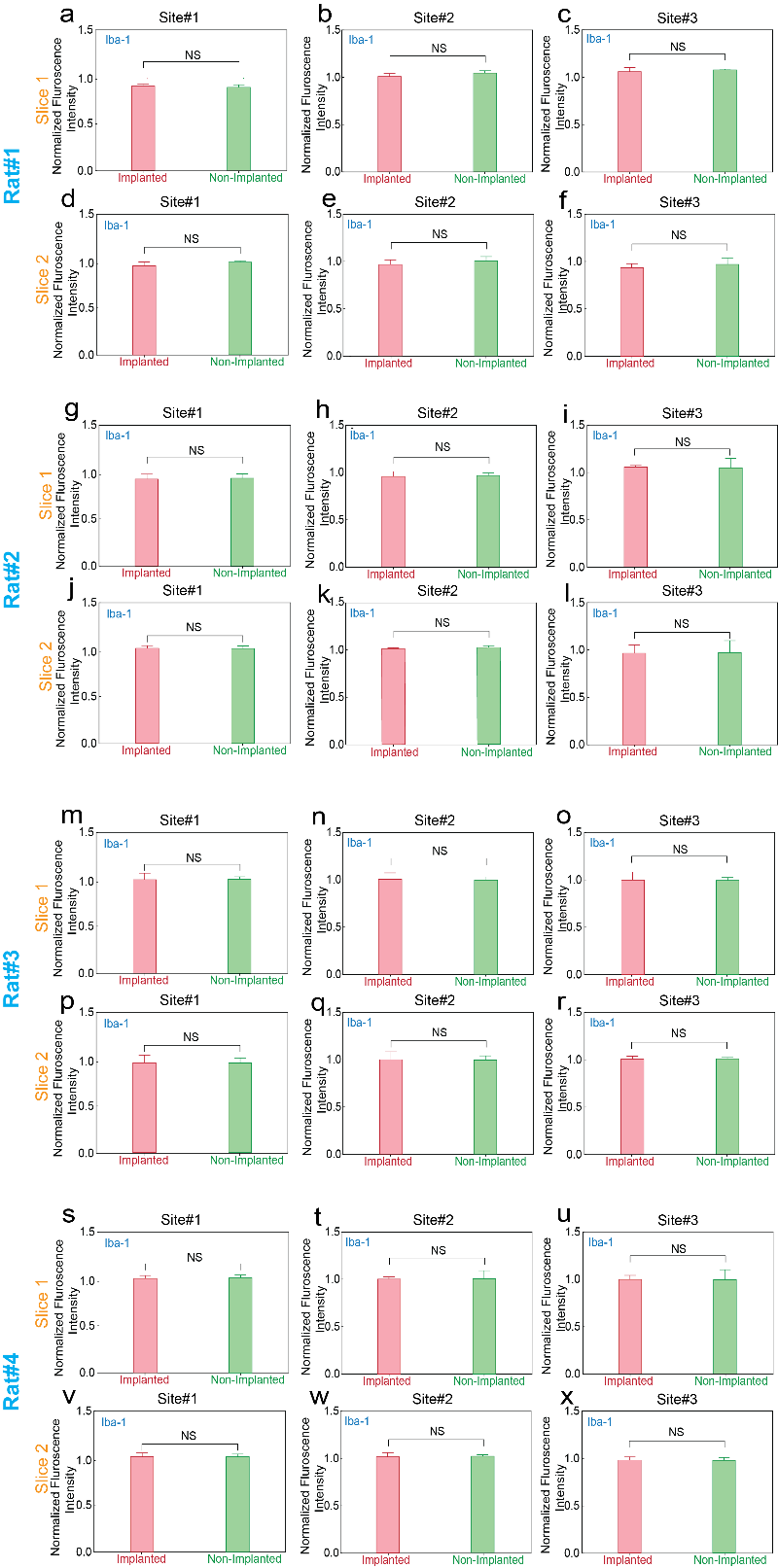
**

**Figure S30. Quantitative analysis of normalized fluorescence intensity of Iba-1 staining near the probe boundary across multiple slices and implantation sites for all rats.** a–f correspond to Rat #1, g–l to Rat #2, m–r to Rat #3, and s–x to Rat #4. Bar plots show the mean ± SD intensity for implanted and non-implanted regions, where ‘NS’ denotes no statistically significant difference (P > 0.05, two-sample t-test).

**Table S1**. Details of 21 patients

|  | Patient  Number from OASIS | Sex | Height (in) | Weight  (lbs) | Age (Years) | Handedness (L/R/A) | BMI  (kg/m^2^) | Health Status |
| --- | --- | --- | --- | --- | --- | --- | --- | --- |
| 1 | 16 | Male | 71 | 254 | 77 | R | 35.43 | Obese |
| 2 | 28 | Male | 64 | 182 | 75 | R | 31.24 | Obese |
| 3 | 35 | Female | 64 | 146 | 80 | R | 25.06 | Overweight |
| 4 | 39 | Female | 61 | 144 | 73 | A | 27.21 | Overweight |
| 5 | 6 | Male | 70 | 203 | 67 | R | 29.13 | Overweight |
| 6 | 349 | Male | 70 | 204 | 76 | A | 29.27 | Overweight |
| 7 | 261 | Female | 62 | 121 | 69 | L | 22.13 | Normal |
| 8 | 330 | Male | 64 | 192 | 81 | L | 32.96 | Obese |
| 9 | 285 | Female | 66 | 127 | 46 | L | 20.50 | Normal |
| 10 | 432 | Female | 70 | 148 | 43 | R | 21.24 | Normal |
| 11 | 638 | Male | 70 | 204 | 42 | R | 29.27 | Overweight |
| 12 | 668 | Female | 64 | 183 | 47 | L | 31.41 | Obese |
| 13 | 922 | Female | 67 | 117 | 46 | L | 18.33 | Underweight |
| 14 | 1059 | Female | 54 | 137 | 49 | L | 33.03 | Obese |
| 15 | 1072 | Male | 68 | 207 | 52 | R | 31.48 | Obese |
| 16 | 1099 | Female | 65 | 184 | 49 | R | 30.62 | Obese |
| 17 | 90 | Male | 74 | 186 | 53 | R | 23.88 | Normal |
| 18 | 91 | Female | 61 | 133 | 84 | R | 25.13 | Overweight |
| 19 | 360 | Male | 70 | 193 | 58 | A | 27.69 | Overweight |
| 20 | 352 | Female | 63 | 137 | 95 | A | 24.27 | Normal |
| 21 | 228 | Male | 69 | 169 | 64 | R | 24.96 | Normal |

**Table S2**. Size of 3D reconstructed brain models of 21 patients

|  | Patient  Number from OASIS | X  (cm) | Y  (cm) | Z (cm) | 3D reconstructed models | |
| --- | --- | --- | --- | --- | --- | --- |
|  |  |  |  |  | Side View | Top View |
| 1 | 16 | 13.72 | 16.85 | 14.54 | 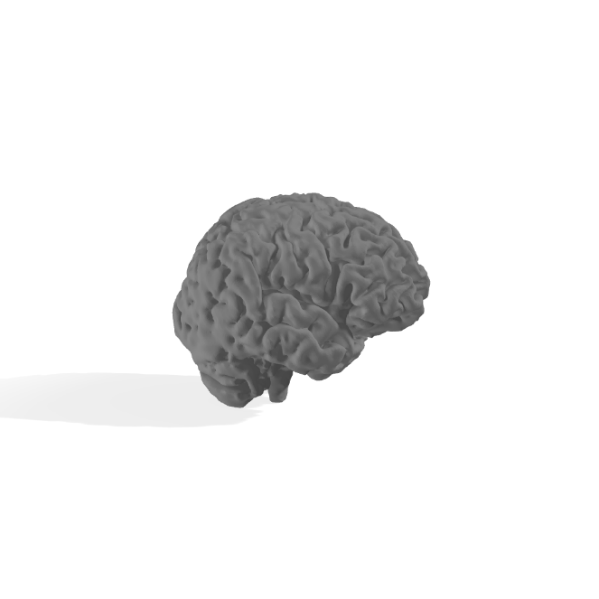 | 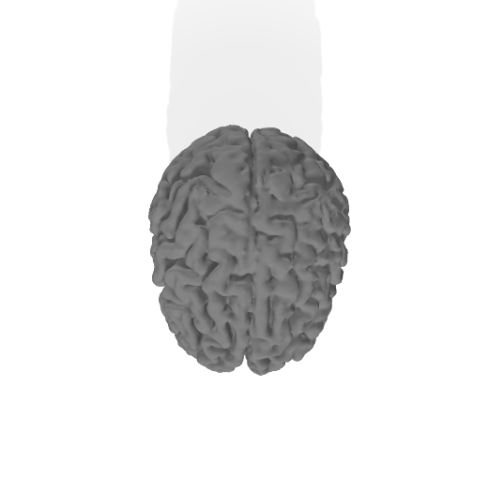 |
| 2 | 28 | 13.97 | 15.84 | 13.26 | 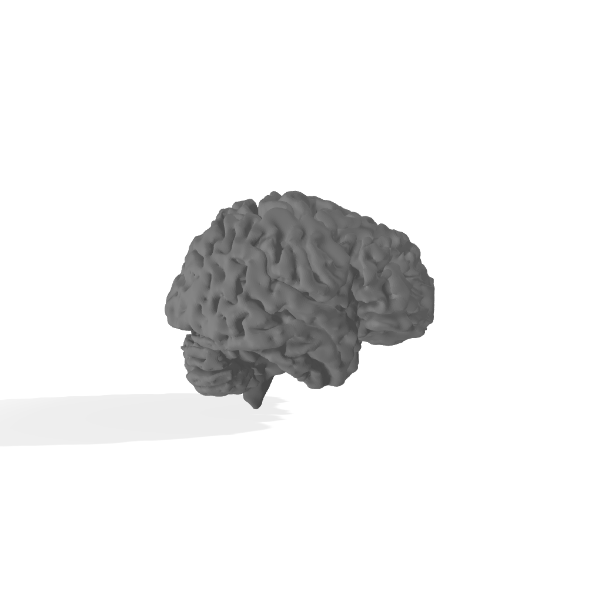 | 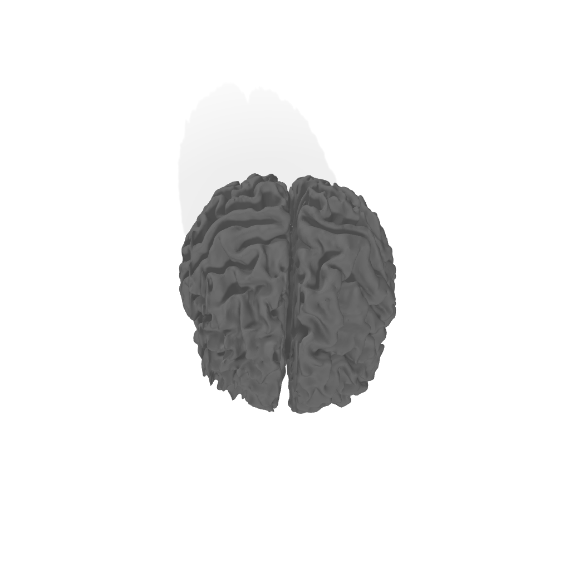 |
| 3 | 35 | 12.47 | 16.38 | 13.34 | 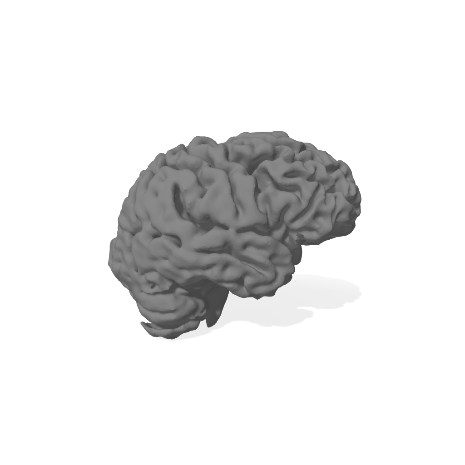 | 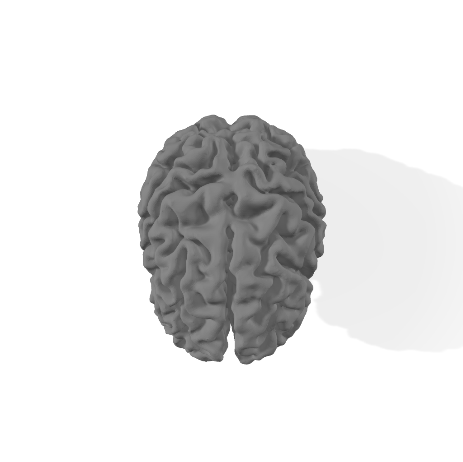 |
| 4 | 39 | 12.95 | 15.63 | 13.06 | 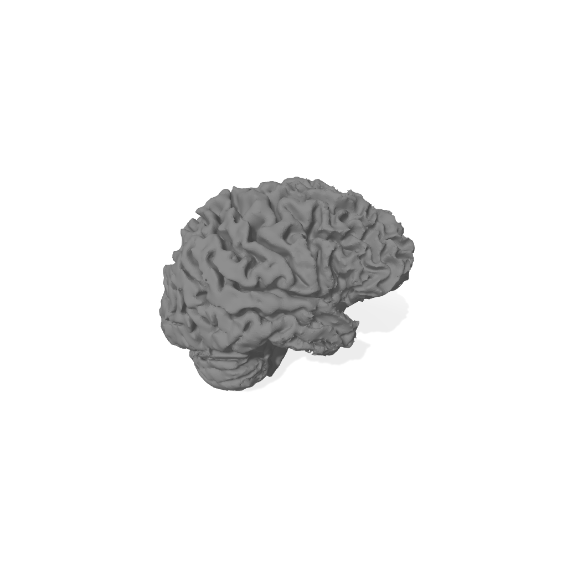 | 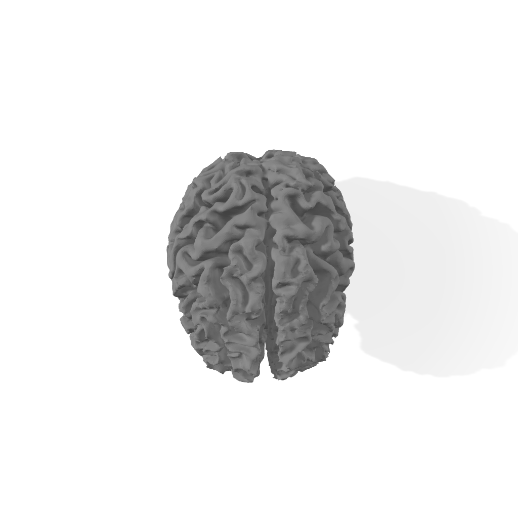 |
| 5 | 6 | 13.35 | 17.67 | 14.45 | 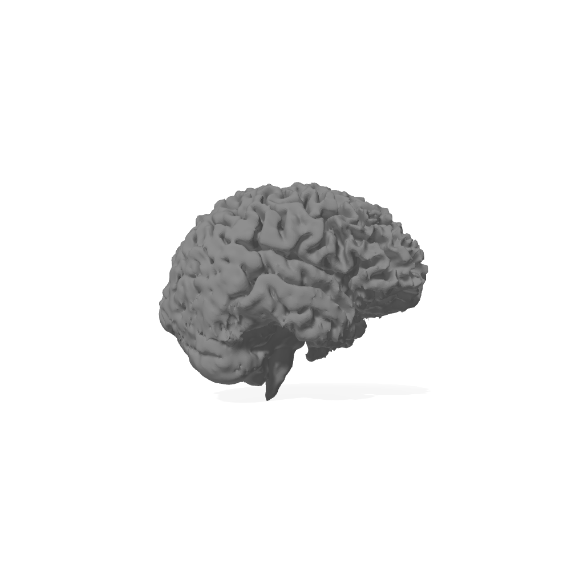 | 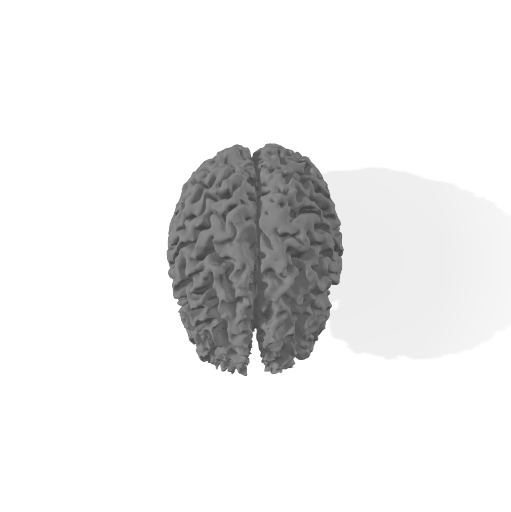 |
| 6 | 349 | 12.78 | 16.19 | 13.29 | 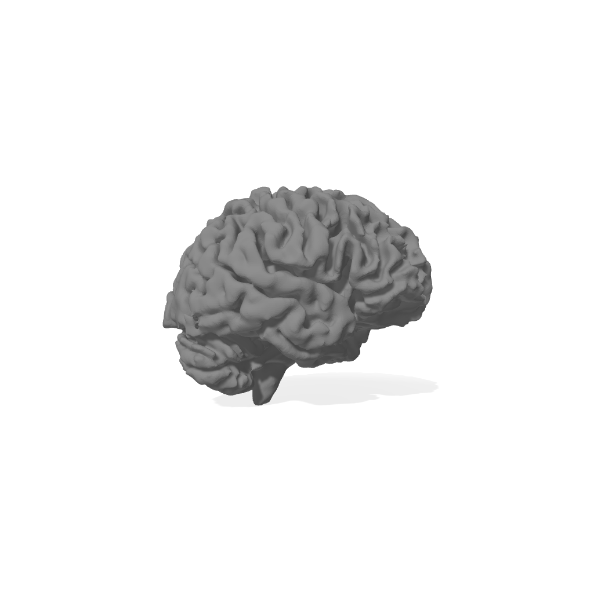 | 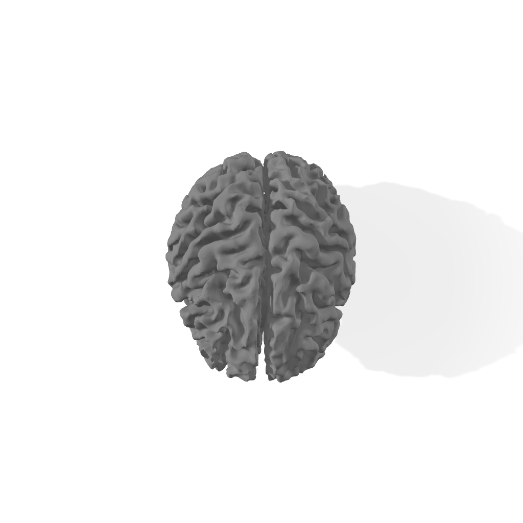 |
| 7 | 261 | 12.24 | 15.55 | 12.27 | 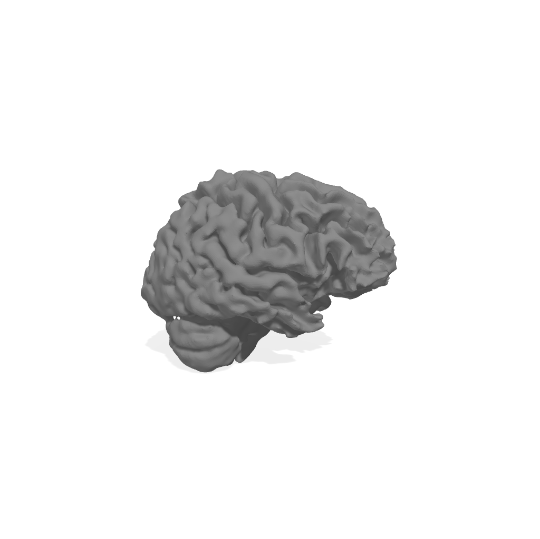 | 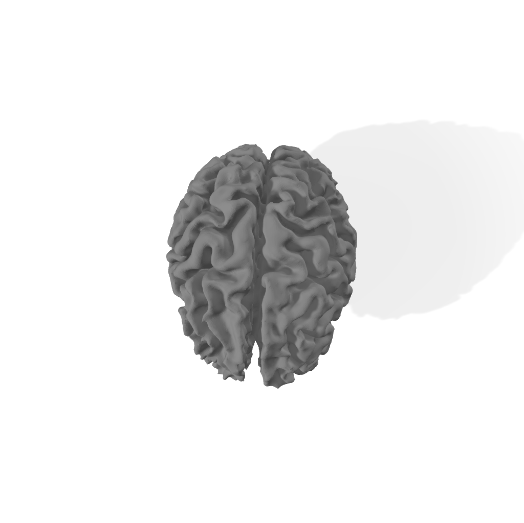 |
| 8 | 330 | 13.12 | 16.86 | 14.08 | 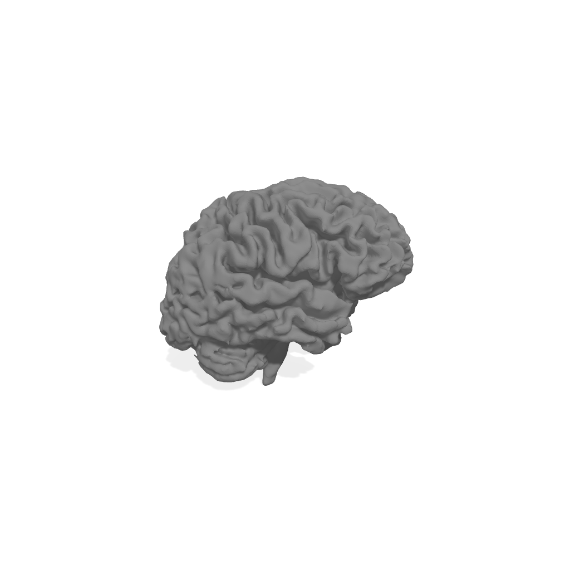 | 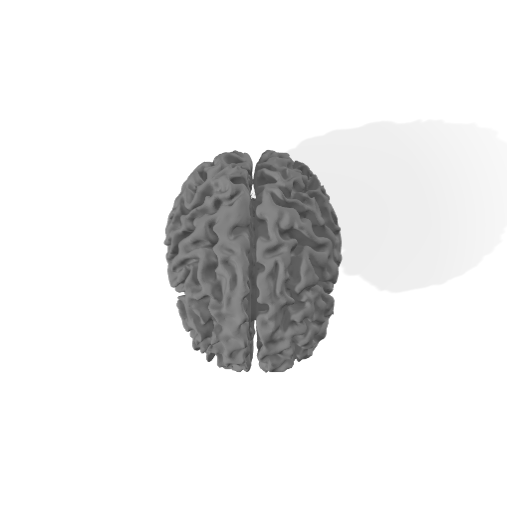 |
| 9 | 285 | 12.29 | 15.60 | 12.41 | 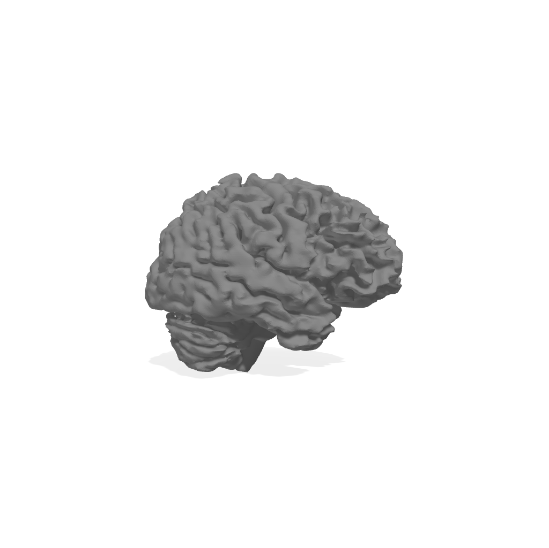 | 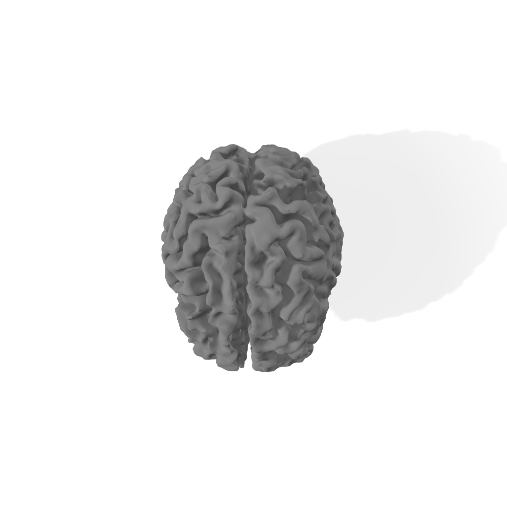 |
| 10 | 432 | 12.31 | 16.02 | 12.71 | 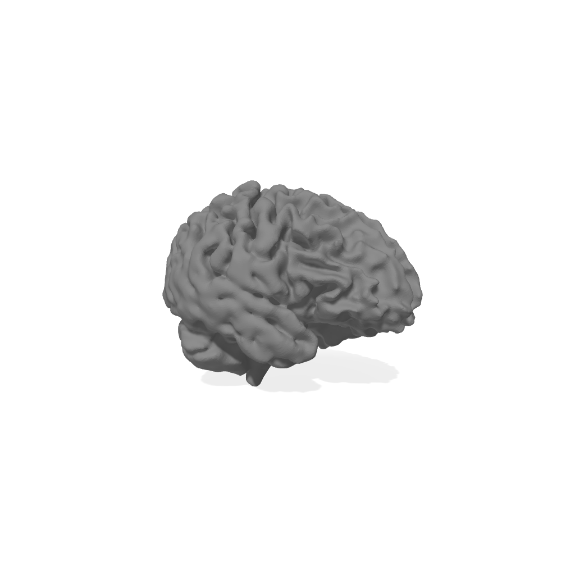 | 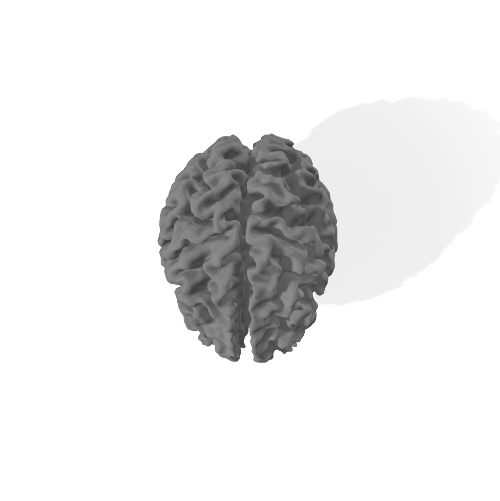 |
| 11 | 638 | 12.93 | 16.91 | 14.4 | 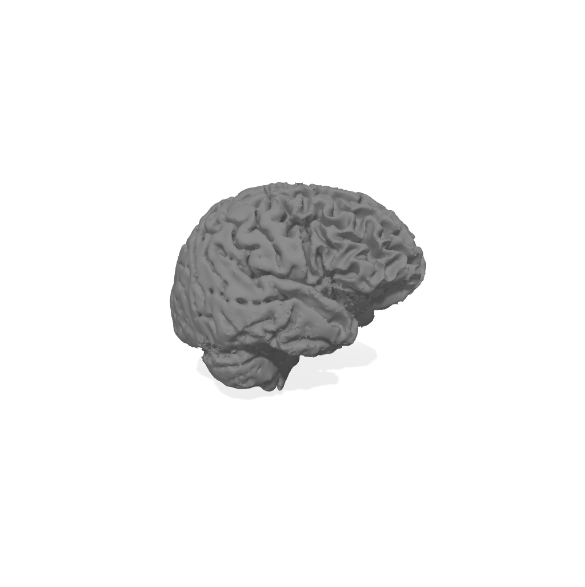 | 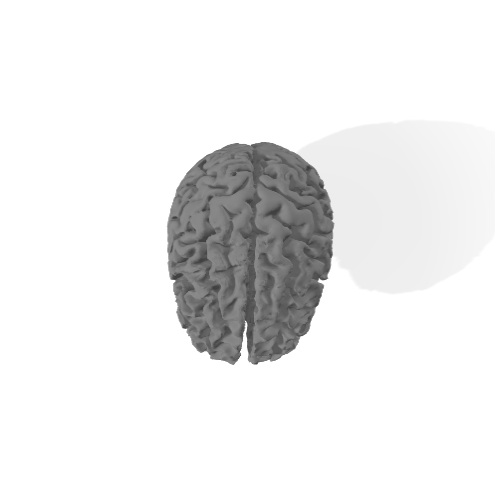 |
| 12 | 668 | 13.16 | 16.36 | 12.78 | 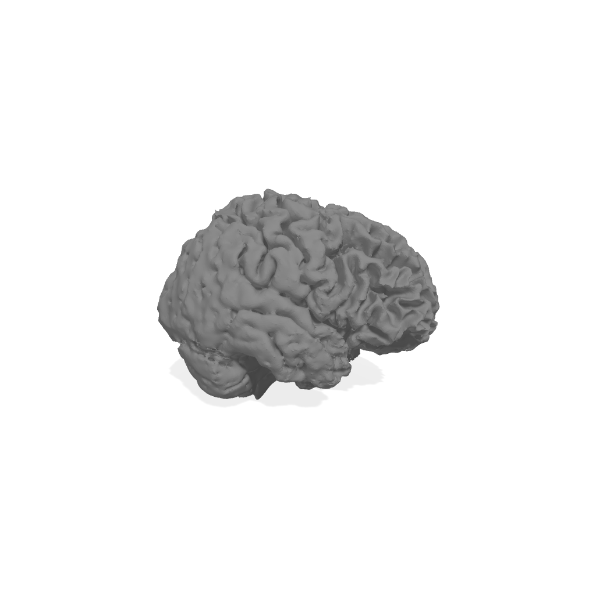 | 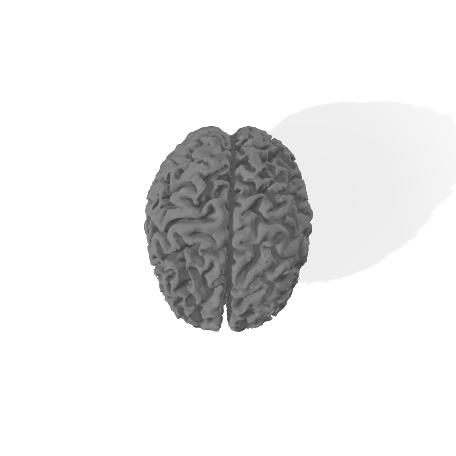 |
| 13 | 922 | 12.22 | 15.97 | 11.68 | 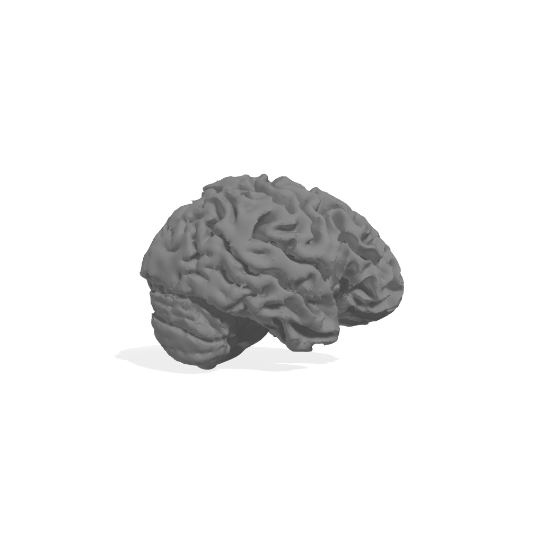 | 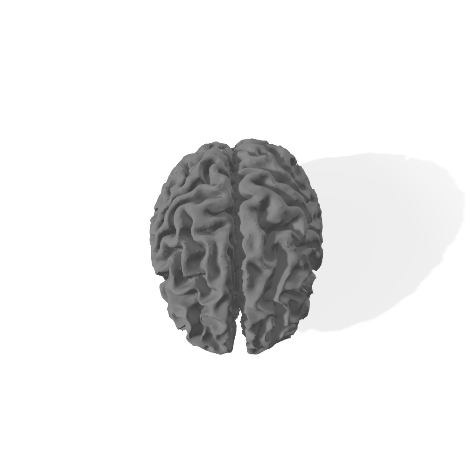 |
| 14 | 1059 | 12.20 | 15.56 | 12.58 | 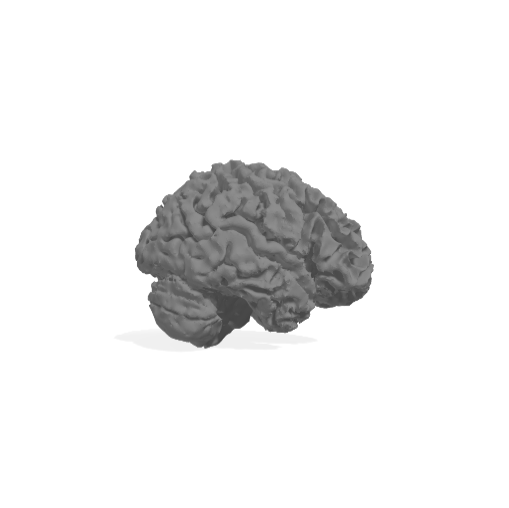 | 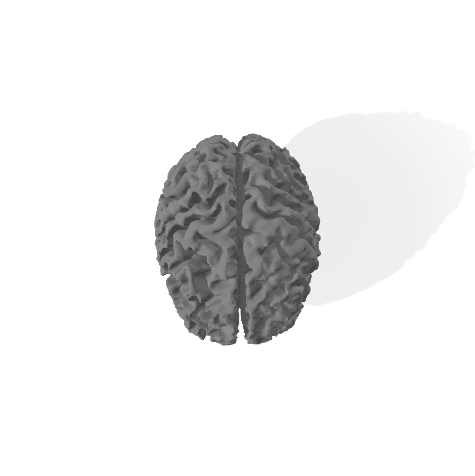 |
| 15 | 1072 | 12.88 | 16.17 | 13.55 | 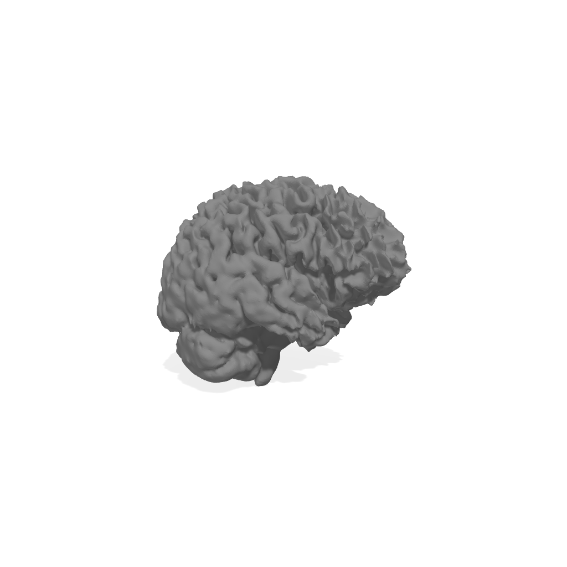 | 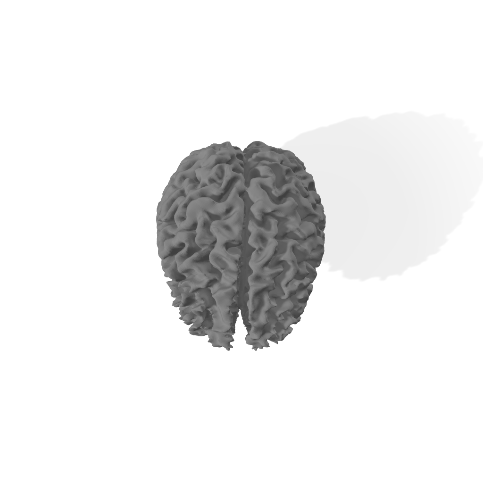 |
| 16 | 1099 | 12.30 | 16.62 | 12.61 | 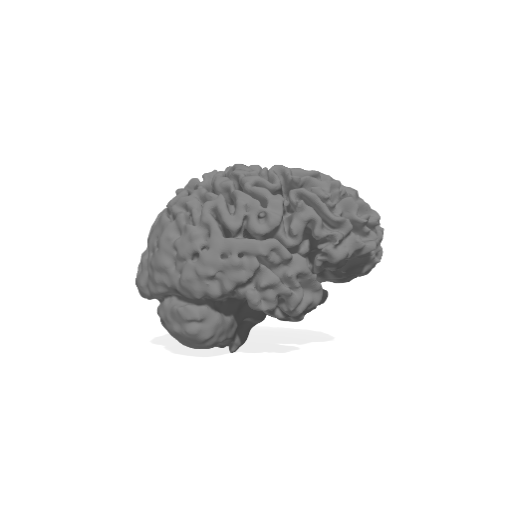 | 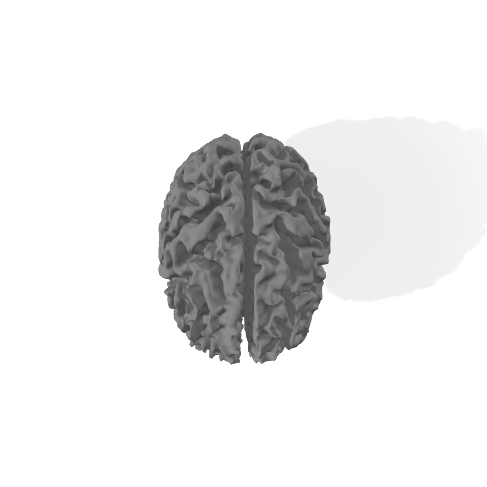 |
| 17 | 90 | 12.91 | 17.3 | 13.45 | 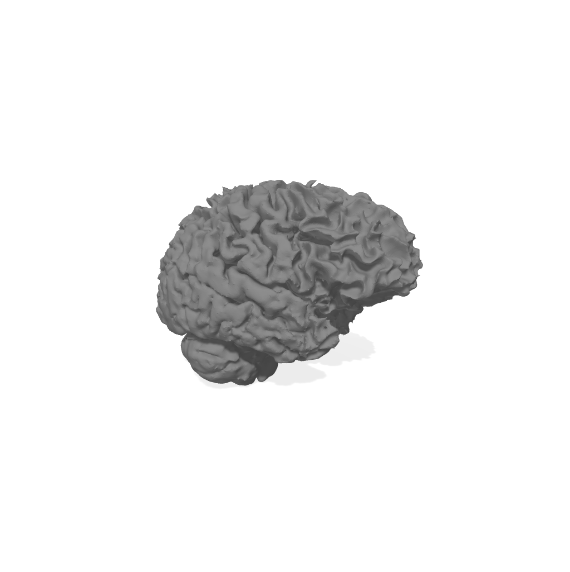 | 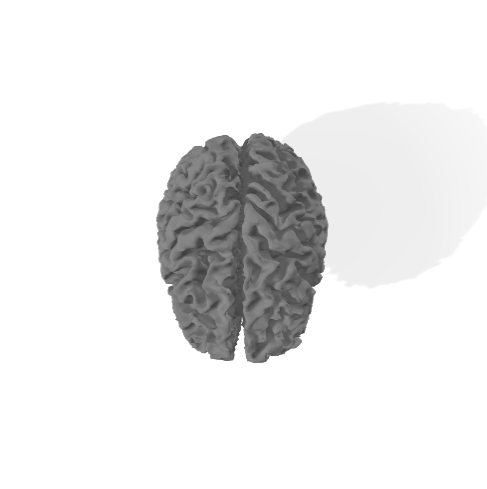 |
| 18 | 91 | 11.83 | 15.45 | 12.41 | 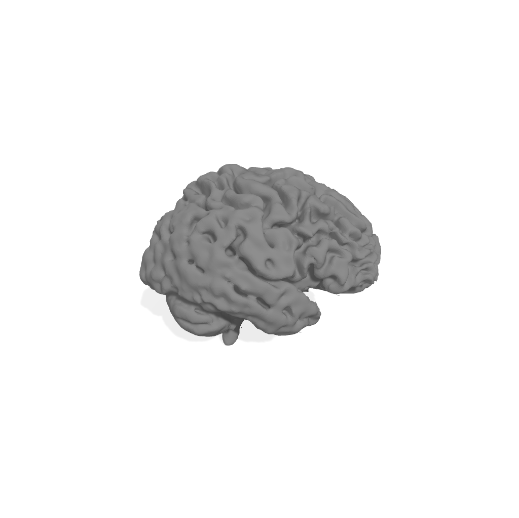 | 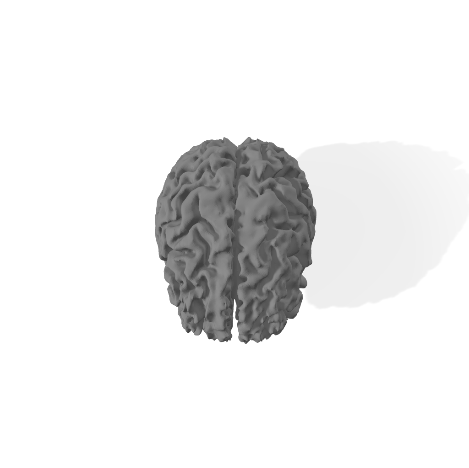 |
| 19 | 360 | 13.39 | 17.71 | 13.53 | 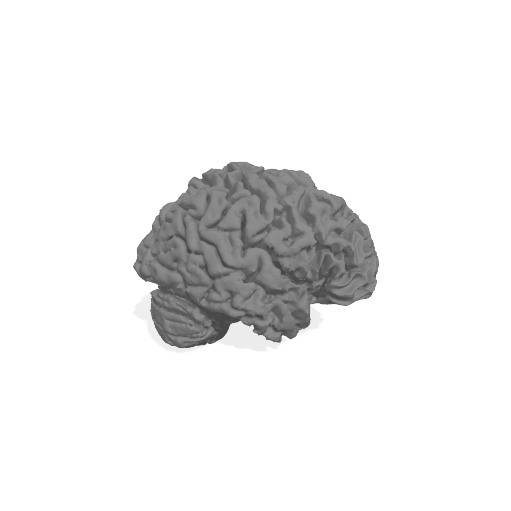 | 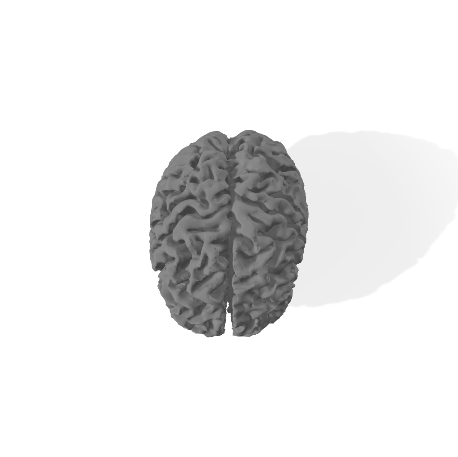 |
| 20 | 352 | 12.09 | 14.97 | 10.89 | 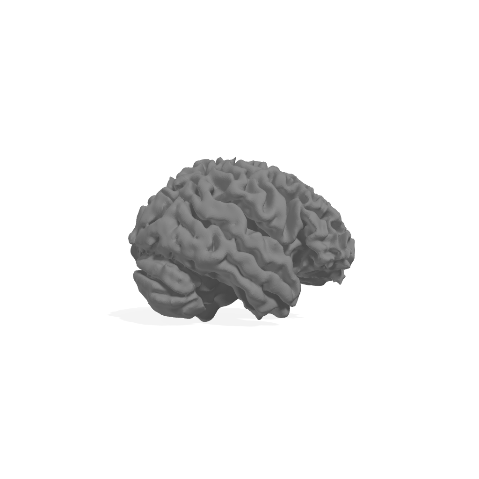 | 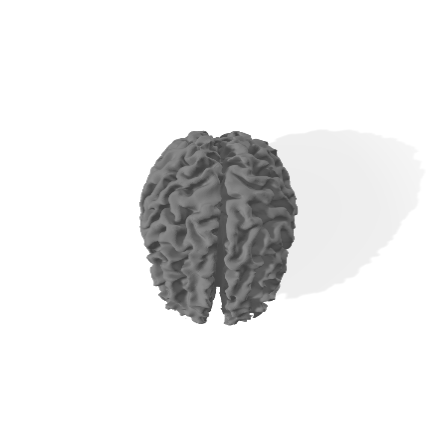 |
| 21 | 228 | 12.61 | 15.83 | 12.42 | 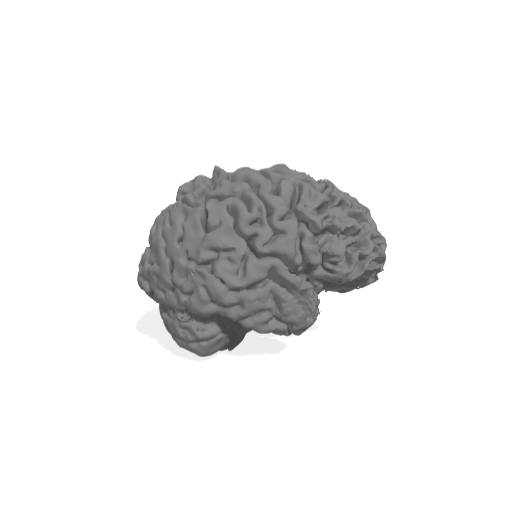 | 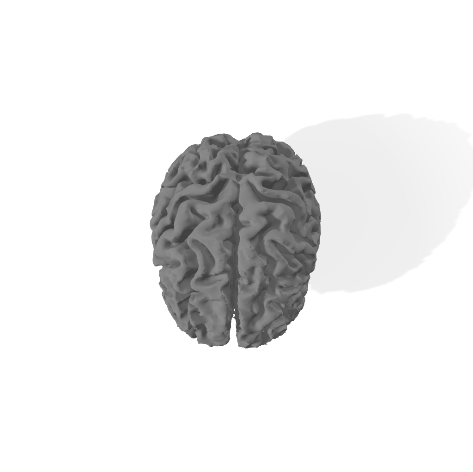 |
